# Normal-Tissue Absence, Rather Than Cross-Patient Prevalence, Limits a Fixed Splice-Junction Panel in Hepatocellular Carcinoma: From a Pre-Specified In Silico Screen to a Genome-Wide Analysis

**DOI:** 10.3390/biomedicines14102396

**Published:** 2026-10-20

**Authors:** Duygu Aygüneş-Jafari

**Affiliations:** Department of Medical Biology, Faculty of Medicine, Ege University, 35100 Izmir, Turkey; duygu.aygunes@ege.edu.tr

**Keywords:** bioinformatics, computational biology, RNA-Seq, transcriptomics, biomarker detection, functional genomics, splice junction, normal tissue reference, hepatocellular carcinoma, statistical power

## Abstract

**Background/Objectives:** A fixed splice-junction panel for hepatocellular carcinoma (HCC) requires junctions recurring across patients and absent from normal tissue; absence is inferred from non-detection in finite references. **Methods:** Across 300 secreted, membrane-protein and HCC-associated genes, 1,263,305 junctions were screened for recurrence (≥30%) in 374 TCGA-LIHC tumours and absence in 50 matched normals and 251 GTEx liver samples. **Results:** No junction met the pre-specified criteria. Widely recurring junctions occurred in normal tissue; of 981 canonical junctions meeting both absence criteria, 740 were seen in one tumour. Post hoc, 8 of the 981 exceeded 11.63%, the prevalence below which non-detection in 50 normals does not demonstrate absence. None reached 30%; they reflect TERT expression, UGT1A first-exon choice, and a UGT2B readthrough of unresolved origin also present in normal breast. Twenty such junctions covered 63.9% of held-out tumours (post hoc), with absence assumed for most. Positive controls from other cancers were recovered. Genome-wide (post hoc), 18 junctions met all criteria, 17 at loci silent in normal liver. **Conclusions:** No panel junction was both recurrent and demonstrably absent from normal liver; genome-wide, such junctions mostly reflected expression of these silent loci, not tumour-specific splicing. Reporting the prevalence above which absence is demonstrable is recommended.

## 1. Introduction

Hepatocellular carcinoma (HCC) is frequently diagnosed at a stage at which curative treatment is no longer possible [1,2]. AASLD guidance recommends semi-annual surveillance of at-risk patients by ultrasound combined with alpha-fetoprotein [1], and the EASL guideline describes personalised surveillance based on individual risk [2]; in a meta-analysis of 32 studies, ultrasound detected early-stage HCC with a sensitivity of 47%, and of 63% when combined with alpha-fetoprotein [3]. A blood test that reads tumour-derived RNA could complement imaging at the surveillance stage, where sensitivity for early-stage disease is limited, and circulating cell-free RNA and circulating mRNA variants are under investigation for this purpose [4,5,6]. Splice junctions are an appealing target class: splicing dysregulation is widespread in cancer [7,8,9,10], a junction is defined by the exact sequence spanning two exon boundaries, and individual splice variants such as AR-V7 [11,12,13] and MET exon 14 skipping [14,15] are already measured in clinical or near-clinical settings. The question addressed in this study is whether splice junctions in HCC can meet a strict criterion of absence from normal tissue while recurring across patients, and the weight of evidence that a normal reference of realistic size provides for such absence.

The design examined is a fixed panel scored as a binary call: junctions are chosen in advance, with a sample counted as positive if any one of them is detected at a fixed read threshold. Three concepts that are often used interchangeably must be separated for such a design. Differential splicing is a change in the inclusion level of an event between tumour and normal tissue; an event can satisfy it while remaining present in normal tissue. Recurrence across patients, referred to below as recurrence or cross-patient prevalence, is the proportion of tumours in which a junction is detected; the term does not refer to clinical recurrence of disease. Tumour-specific absence is the non-detection of the junction in normal tissue. A binary panel requires the second and the third together, because a positive at any target is a positive overall, and a junction present in normal tissue returns the same result there; an assay that quantifies an inclusion ratio against a reference distribution does not need that property. The recurrence of a single junction is also distinct from the coverage of a panel, since a union of individually uncommon junctions can reach patients that no single junction reaches.

Existing studies do not resolve whether junctions meeting both requirements exist in HCC. Splicing events in HCC have been catalogued at scale and tested in peripheral fluid, with selection by differential inclusion [16]. Pan-cancer neojunction catalogues were called against a GTEx-only reference without a recurrence requirement [17], recurrent tumour-wide splice neoantigens have been described at particular loci [18], and a three-study lineage in clear cell renal cell carcinoma (ccRCC) carried the target class from discovery to a plasma assay without a per-patient matched-normal filter [19,20,21]. The reference matters in HCC in particular: in one proteogenomic cohort, 94% of patients had at least one underlying liver disease [22], and tissue adjacent to the tumour is therefore not a neutral comparator; analyses that rely on independently processed references are also subject to batch effects [23]. The genomic landscape of the tumour has been characterised at cohort scale [24,25,26]. To the author’s knowledge, no study in HCC has applied an absence criterion against matched adjacent normal tissue together with a recurrence requirement or quantified the evidence that non-detection in a normal reference provides.

The primary objective was to determine, under thresholds fixed in advance, whether any junction in a 300-gene panel of secreted, membrane-protein and HCC-associated genes is both recurrent in TCGA-LIHC tumours and absent from matched adjacent normal tissue and from GTEx liver, with the two requirements evaluated separately rather than as one combined filter. The secondary objectives, addressed post hoc, were to quantify the evidence that non-detection in each normal reference carries as a function of candidate prevalence, to estimate the coverage attainable by a union of junctions, and to apply the same criteria to every junction in the genome. The primary scope is one panel, one gene class, and one cohort; the genome-wide application tests whether the outcome is specific to the panel, and the expected contribution is a quantified account of the requirement that limits such a panel and of where, across the genome, that requirement can be met. A feasibility check on control junctions in public plasma cell-free RNA was performed separately; it is summarised in Section 3.8 and reported in full in Supplementary Section S3. All thresholds were fixed in an internal repository record before the data they apply to were examined, and every analysis added after those outcomes were known is labelled post hoc where it appears.

## 2. Materials and Methods

### 2.1. Data Sources and Genome Build

Tissue junction counts were obtained from Snaptron [27], which serves the uniformly processed recount3 [28] compilations. The TCGA compilation (tcgav2) and the GTEx compilation (gtexv2) [29] were used, both aligned to the GRCh38 primary assembly and annotated with GENCODE v26 [30]. Using two compilations processed through a single pipeline reduces the systematic differences that arise when tumour and reference-tissue datasets are combined from independently processed sources; differences in sequencing depth and read length between the cohorts are quantified in Section 3.4.

Sample metadata were taken from the metadata tables distributed for each compilation.

### 2.2. Sample Groups

Three groups were defined from the compilation metadata, using the field gdc_cases.project.project_id to identify TCGA-LIHC and the field cgc_sample_sample_type to classify sample type. Tumour samples were TCGA-LIHC samples with a sample type containing “tumor” or “primary” (*n* = 374; 371 primary tumours and 3 tumours from recurrent disease, analysed as one group). Matched normal samples were TCGA-LIHC samples with a sample type containing “normal” (*n* = 50). Healthy tissue samples were GTEx samples annotated as liver in SMTS or SMTSD (*n* = 251).

Groups were defined by sample rather than by patient, and prevalence figures are counts of tumour samples; the sample-to-patient mapping, on the TCGA patient barcode, was used in the paired comparison of Section 3.3.2, in the patient-grouped split-half evaluation (Section 2.11), and in the copy-number comparison (Section 2.10). The 374 tumour samples come from 371 patients: one patient contributes three samples and one contributes two.

### 2.3. Gene Panel

Discovery was restricted to a gene panel defined before any junction was retrieved. Candidate genes were drawn from the Human Protein Atlas (HPA) [31] predicted-secreted and predicted-membrane protein classes (150 and 100 genes, respectively, each ranked by liver nTPM descending with gene symbol ascending as the tie-break), supplemented with 50 hepatocellular carcinoma-associated genes from Open Targets Platform [32] disease associations (MONDO_0007256, ranked by overall association score). The eight sensitivity-control genes (ALB, SERPINA1, APOA1, FGA, FGB, FGG, HP, TF) were all selected by the ranking rule. The panel was capped at 300 entries in total. The selection rule was written to file before it was applied, and the resulting panel with its source columns is provided as gene_panel.tsv.

Of the 300 panel genes, 4 (ATP5MF, ATP5MK, ATP5MC2, ATP5MC3) did not resolve against GENCODE v26 and were not queried; the remaining 296 were queried, together with AR, a specificity-control gene of the plasma check (Supplementary Section S3). Of the 296, 13 are mitochondrially encoded protein-coding genes (MT-ATP6, MT-ATP8, MT-CO1 to MT-CO3, MT-CYB, MT-ND1 to MT-ND6, and MT-ND4L), which are not spliced; the mitochondrial genome nevertheless returns 313,903 of the 1,263,305 junction records and 24 of the 981 eligible junctions (Section 2.6). None of the eight sensitivity-control genes is affected.

The panel defines the genic regions that are queried, not the identity of every gene a returned junction touches. A region query returns every junction that overlaps a panel-gene region, and a returned junction need not have either end in a panel gene: the CFH to CFHR4 junction (Section 3.2) ends in CFHR4, which is not a panel gene, and the UGT2B11 to UGT2B27P junction (Section 3.3.2) has neither end in a panel gene and was returned because it spans the panel gene UGT2B7.

### 2.4. Pre-Specified Thresholds

All analysis thresholds, including those for the Snaptron tissue queries on which the primary outcome rests, were written to thresholds.json inside the analysis repository before the data they apply to were examined; the file was not modified afterwards and is deposited unchanged with the analysis code.

“Pre-specified” refers to this record; no registration was filed with an external registry. Sample criteria were recorded as integer sample counts rather than percentages.

A junction was counted as present in a tumour at five or more reads and was required to be present in at least 112 of 374 tumours (30% of 374 = 112.2, floor applied). A normal sample was counted as carrying signal at two or more reads; single reads were treated as within the noise floor of RNA sequencing, since index hopping, template switching, and mismapping each generate isolated reads, and a one-read observation is not evidence of expression. A candidate was required to carry signal in at most 2 of 251 GTEx liver samples (1% of 251 = 2.51, floor applied) and in none of the 50 matched normals. The two absence criteria therefore differ in their effective stringency, and the stricter of the two is applied to the smaller and deeper cohort. Negative controls were defined as annotated junctions present at five or more reads in at least 30% of both tumours and matched normals, with a tumour-to-normal detection-rate ratio between 0.8 and 1.25 inclusive. Alignment-based counting required an anchor of at least 8 bp on both sides of the junction, and alignment-free counting used k = 31.

The 30% recurrence floor was fixed in advance as the operational definition of a single target that could contribute without a panel. Its position relative to published practice is as follows. Discovery studies that define shared or public tumour-specific junctions and transcripts have used prevalence thresholds of 1% [33], 10% [18,34,35], and more than 15% [36] of a cohort; the present floor is two to thirty times more stringent, because the intended use is diagnostic rather than the nomination of immunotherapy targets. The prevalence of a target caps the sensitivity of a one-target binary assay. A floor of 30% lies at the frequency of the TP53 and CTNNB1 alterations in HCC (25% to 30%) and below that of TERT promoter mutation (44% to 60%) [24,25,26]. It also lies below the early-stage sensitivity of 39% to 64% reported for alpha-fetoprotein [37]; a one-target assay below the floor could therefore not approach the sensitivity of the single marker already in use. The GTEx absence criterion of at most 1% of samples follows the convention of the pan-cancer neojunction catalogues [17,18]. A panel does not require the floor of each of its members, and the pre-specified criterion is therefore a single-target endpoint. Its influence on the outcome is examined by the sweep of Section 3.6, and the union of targets, which is the clinically relevant quantity for a panel, is examined post hoc in Section 3.5.

A canonical splice motif was defined as a donor-acceptor dinucleotide pair of GT..AG on the plus strand or CT..AC on the minus strand, as reported by Snaptron. Snaptron returns these dinucleotides in the letter case of the underlying reference sequence; a motif lying inside a soft-masked (RepeatMasker) interval is therefore reported in lower case. The filter applied in this study matched upper case only, and therefore also excluded junctions whose splice sites fall within soft-masked repeats. Junctions with splice sites in repeat-masked sequence are not practical targets for a primer-based or probe-based assay. The effect of the upper-case rule was quantified post hoc and is reported in Supplementary Section S2: relaxing the match to be case-insensitive enlarges the eligible set (Section 2.6) from 981 to 1292 junctions and leaves the zero outcome unchanged and the other quantities listed there unchanged or within one percentage point; eligible-set counts in the main text refer to the set of 981.

Two decision thresholds were fixed in the same file: detection of at least six of the eight sensitivity-control genes and detection of at least 20% of surviving positive candidates, with a minimum of three in absolute terms.

### 2.5. Junction Retrieval

Junctions were retrieved through the Snaptron region interface, one request per panel gene against each compilation, with no samples_count or coverage pre-filter, because a junction removed before counting is indistinguishable from an absent junction.

The interface applied no default samples_count filter: an unfiltered query of the ALB region in tcgav2 and a query with samples_count>:1 returned the identical set of 3924 rows, whereas samples_count>:100 reduced the same query to 80 rows. The per-sample counts themselves carry a minimum that depends on intron length, which is examined in Section 3.6.

Requests were issued sequentially, and the per-junction records returned by all queries were consolidated into one table per compilation (junctions_tcgav2.tsv.gz and junctions_gtexv2.tsv.gz). These tables and the cached per-gene responses underlie the pre-specified cascade and the panel-level analyses; the post hoc analyses of Section 2.8, Section 2.9 and Section 2.10 used the further queries and sources listed in Supplementary Section S4. Reanalysis therefore issues no further panel requests, and the analysis is reproducible against a fixed snapshot of a live service. Detection frequencies were computed within the defined sample groups by parsing the per-junction sample list, never across the whole compilation.

### 2.6. Candidate, Eligible, and Control Sets

Positive candidates were junctions meeting all three pre-specified criteria of Section 2.4. Normal-absent junctions are those meeting both normal-absence criteria. The eligible set comprises canonical-motif (Section 2.4), normal-absent junctions detected in at least one tumour at the pre-specified five-read signal threshold. The evidence floor, used to interpret the raw normal-absent fraction (Section 3.1), comprises junctions with a GT..AG or CT..AC motif in the tcgav2 compilation, matched without regard to letter case, and at least five reads in at least one sample. An eligible junction is termed absence-supported when its tumour prevalence exceeds the informative prevalence of the matched-normal criterion (post hoc; Section 2.10 and Section 3.4). The control sets used by the plasma feasibility check (negative, sensitivity, and specificity controls) are defined in Supplementary Section S3.

### 2.7. Plasma Cell-Free RNA Feasibility Check

A feasibility check on control junctions was performed on public plasma cell-free RNA libraries from the European Nucleotide Archive, study SRP239389 (Gene Expression Omnibus series GSE142987) [38]. One liver-cancer library, designated in advance, defined the pre-specified sensitivity criterion (detection of at least six of eight liver-abundant control genes; Section 2.4); three further cancer libraries and one healthy-donor library were processed post hoc. Reads were trimmed with fastp 1.3.6 [39] and aligned with HISAT2 2.2.3 [40,41], alignments were processed with SAMtools 1.24 [42,43], and junction-spanning reads were counted by two methods, alignment-based extraction with regtools [44] and, for the four cancer libraries, alignment-free exact 31-mer matching against junction-spanning reference sequences. Library selection, control sets, sequence construction, the genome-wide uniqueness screen, quality control, and counting are described in full in Supplementary Section S3.

### 2.8. Independent Splice-Site Annotation Check (Post Hoc)

For the UGT2B11 to UGT2B27P junction (Section 3.3.2), each of the two splice sites was queried independently against the Ensembl REST annotation service [45] (rest.ensembl.org,/overlap/region) to test whether each coordinate alone, irrespective of its pairing with the other, coincides with an annotated exon boundary in any transcript within a 200 bp window (verify_junction_splice_sites.py; junction_splice_site_verification.tsv). Because that service serves the current Ensembl annotation rather than GENCODE v26, each splice site was also verified against GENCODE v26 itself, from its transcript models, and cross-checked against Ensembl release 88 and against the per-site annotation fields of the tcgav2 and gtexv2 compilations (70_gencode_v26_splice_sites.py; junction_splice_site_gencode_v26.tsv). The GENCODE v26 result is reported in Section 3.3.2, and the current-annotation query is recorded as supporting evidence.

### 2.9. Positive-Control Junctions (Post Hoc)

Two established oncogenic junctions were queried to test whether the retrieval and the thresholds recover a known event: EGFRvIII (EGFR exon 1 joined to exon 8), the most common EGFR variant in glioblastoma [46], and MET exon 14 skipping (exon 13 joined to exon 15) in lung adenocarcinoma [14,15]. Intron coordinates were derived from the Ensembl canonical (MANE Select) transcripts ENST00000275493 and ENST00000397752 (Ensembl release 116, GRCh38) and checked against the known sizes of the deleted segments (801 bp for EGFR exons 2 to 7; 141 bp for MET exon 14). Each junction and its two flanking canonical junctions were retrieved by exact-coordinate query from tcgav2 and gtexv2 and counted with the read floors of Section 2.4 (five reads in tumour samples, two reads in normal samples) in the corresponding TCGA project, in its solid-tissue normal samples and in the corresponding GTEx tissue. Tumour samples were primary tumours or tumours from recurrent disease; 53 TCGA samples without a resolvable sample type were excluded. For GNAS and RPL22, whose published neojunctions carry no coordinates, the retrieved junctions were searched for one sharing its donor with a canonical junction present in at least 112 of 374 tumours and with an acceptor displaced by exactly 2 nt (GNAS) or 6 nt (RPL22), as the source study describes.

### 2.10. Additional Reference Analyses (Post Hoc)

Normal-side read floor. The eligible set and the pre-specified cascade were recomputed with the matched-normal floor and the GTEx floor each set to one, two, three, or five reads (16 combinations), the tumour floor being held at five reads. Per-sample counts were taken from the recount3 project-level junction matrices of TCGA-LIHC and GTEx liver [28], from which the tcgav2 and gtexv2 compilations are built; carrier counts recomputed from these matrices were identical to the Snaptron-derived counts for every panel junction (776,855 junctions in the consolidated tcgav2 table and 770,232 in the gtexv2 table; 117,448 and 112,161 of them are present in the two project matrices, and the remainder have no carrier in either). For the 25 eligible junctions reaching the joint informative prevalence, carriers were also counted at one, two, three, and five reads by exact-coordinate query.

Reference power. For an absence criterion that tolerates at most k carriers among n normal samples, the minimum informative tumour prevalence was obtained by solving P(*X* ≤ *k* | *n*, *f*·*p*) = alpha for a binomial variable X, with k = 0 and *n* = 50 for matched normal tissue and k = 2 and *n* = 251 for GTEx liver. For the two references jointly, the product of the two probabilities was used; this treats the two cohorts as samples of one normal population, which their differences in read length and depth do not guarantee, and the joint value is therefore reported as a bound rather than as a pooled estimate.

Reference concordance. Detection of each retrieved junction, defined as at least two reads in at least one sample, was compared between the 50 matched normal and the 251 GTEx liver samples by cross-tabulation and by Spearman correlation of detection fractions, over all retrieved junctions and over those passing the evidence floor (Section 2.6). The comparison was performed over the panel junctions and repeated genome-wide, over all junctions on chromosomes 1 to 22, X, Y, and M in the recount3 matrices, with the same read criterion and the motif taken from the TCGA-LIHC project matrix.

Tissue distribution and locus annotation. The eight absence-supported junctions were retrieved by exact-coordinate query from gtexv2, and their carriers were tabulated by tissue across all 19,214 GTEx samples, including 852 whole-blood samples. For the UGT2B junction, carriers were also tabulated by project and sample type across tcgav2 (10,606 tumour and 742 normal samples) and in the srav3h compilation (316,447 samples), whose free-text metadata were searched by keyword because no curated tissue annotation is available. The two splice sites of the UGT2B junction were compared with segmental duplications, with structural variants of the Database of Genomic Variants [47] and with alternate-haplotype sequences annotated on GRCh38, retrieved from the UCSC Genome Browser [48] (tracks genomicSuperDups, dgvMerged, and altLocations), and with the positions of the UGT2B genes from Ensembl release 116 [45]. Copy number of the junction interval was examined in open-access Affymetrix SNP 6.0 copy-number segments for TCGA-LIHC from the Genomic Data Commons (GRCh38; 379 tumour, 306 blood-derived normal and 83 solid-tissue normal DNA samples) and, for tumour DNA, in the hg19 segments of the UCSC Xena hub that retain germline copy-number probes. The length-weighted mean log2 ratio of the interval between the two splice sites was compared between patients whose tumour carries the junction at three or more reads and the remaining patients, in tumour DNA and in normal DNA, and the UGT2B17 germline deletion served as a positive check that the data resolve a deletion at this locus. The same measure was computed over UGT2B28 and UGT2B17 and compared by carrier status (75_ugt2b28_copy_number.py).

Read floor by intron length. The minimum per-sample read count recorded by the compilations was determined by exact-coordinate query for thirteen junctions with introns of 0.3 to 272 kb, and the junctions of the retrieved, recurrent and eligible sets were counted by intron-length class (Supplementary Table S12).

Genome-wide cascade. The pre-specified cascade of Section 2.4 was applied, with unchanged thresholds and without a motif or annotation filter, to every junction on chromosomes 1 to 22, X, Y, and M in the recount3 matrices of TCGA-LIHC and GTEx liver. Applied to the panel junctions, the same computation reproduces the pre-specified counts (3314, 1 and 0) and the identical set of 3314 junctions meeting the recurrence criterion. For each junction meeting all three criteria, tumour carriers were recounted from the per-sample matrix, carriers were tabulated by project and sample type across tcgav2 and by tissue across gtexv2, and the GENCODE v26 genes overlapping the junction were identified. Expression of the surrounding locus was measured as the mean number of reads per sample over all other junctions on the same strand within the span of the overlapping genes, or within 10 kb of the junction where no gene overlaps, in tumours, matched normal samples, and GTEx liver (76_genome_wide_cascade.py).

### 2.11. Pre-Specified and Post Hoc Analyses

The pre-specified analyses are the cascade of Section 2.4 and the plasma feasibility criterion of Section 2.4 and Section 2.7; Section 2.8, Section 2.9 and Section 2.10 are post hoc in their entirety. Elsewhere, the following analyses were performed after the pre-specified result was obtained: the tumour-side read-threshold sweep, the paired matched-normal comparison, the descriptive TERT locus comparison, the artefact tests at the CFH and UGT2B loci, and the cohort-level locus comparison of Table 1. Also post hoc are the motif composition of the normal-absent set (Supplementary Figure S1); the motif case-sensitivity check of Section 2.4; the union (set-cover) analysis of Section 3.5 and its normal-side criterion grid; the recurrence-floor, gene-selection, and matched-normal allowance recomputations of Section 3.6; and, for the plasma check, the processing of three further cancer libraries, the summation of counts across the four, and the cancer-versus-healthy-donor contrast. None of these modified thresholds.json, and none altered the pre-specified outcome. Each is labelled post hoc where it appears.

In the split-half evaluation of Section 3.5, the candidate pool was defined on all 374 tumours. A junction not detected in the training half has zero marginal gain and is never selected by the greedy rule; restricting the pool to junctions detected within the training half therefore leaves the selected panels and the held-out coverage unchanged. The evaluation was also repeated with the eight-junction subset re-derived within each training half (at least 22 of 187 tumours), with splits drawn by patient, and with both (74_set_cover_nested.py; Supplementary Table S16).

### 2.12. Statistics

Group comparisons used the Mann–Whitney U test, proportions Fisher’s exact test, and method agreement the Spearman rank correlation. Group comparisons are exploratory and were not corrected for multiple testing. Locus coverage is expressed as reads per 10^6^ panel junction reads, the denominator being the per-sample sum of reads over all junctions returned by the panel query. The comparison of TERT locus reads between junction carriers and non-carriers is reported descriptively, without a test, because the grouping variable is derived from the same reads. The null probabilities accompanying the paired matched-normal counts (Section 3.3.2) are illustrative of relative evidential weight and are not formal hypothesis tests; because the junctions were selected for zero matched-normal carriers, the paired counts are not independent of that selection. The absence-power criterion of Section 3.4 is a deterministic function of prevalence, applied to each eligible junction without multiplicity adjustment. Binomial confidence bounds on zero-carrier observations are exact (Clopper–Pearson) [49], and one-sided at 95%. Power, as used here, denotes the minimum candidate prevalence at which the observed number of normal carriers rejects the stated null at level alpha, not power against a specified alternative. Multi-target panels (Section 3.5) were built by the greedy approximation to the maximum-coverage problem, which at each step adds the junction covering the largest number of not-yet-covered tumours; for any panel size, the greedy panel covers at least (1 − 1/e) of the coverage attained by the optimal panel of that size [50,51].

### 2.13. Computing Environment

Software versions were Python 3.12.14, pandas 3.0.5, numpy 2.5.2, scipy 1.18.0, pysam 0.24.0, HISAT2 2.2.3, SAMtools 1.24, htslib 1.24, fastp 1.3.6, and seqkit 2.13.0.

### 2.14. Data and Code Availability

All source data are public and require no registration. Scripts, the frozen threshold record, the consolidated junction tables, all intermediate tables, figure-generating scripts, and measured runtimes are deposited on Zenodo. A per-item mapping of each table and figure to its underlying data file is given in Supplementary Section S4.

## 3. Results

### 3.1. Study Overview and Primary Screening Outcome

The discovery arm queried 1,263,305 splice junctions across the 296 queried panel genes and AR (Section 2.3) in three cohorts drawn from recount3/Snaptron (GRCh38, GENCODE v26): 374 TCGA-LIHC tumours, 50 matched adjacent normals, and 251 GTEx liver samples.

**Figure 1:**
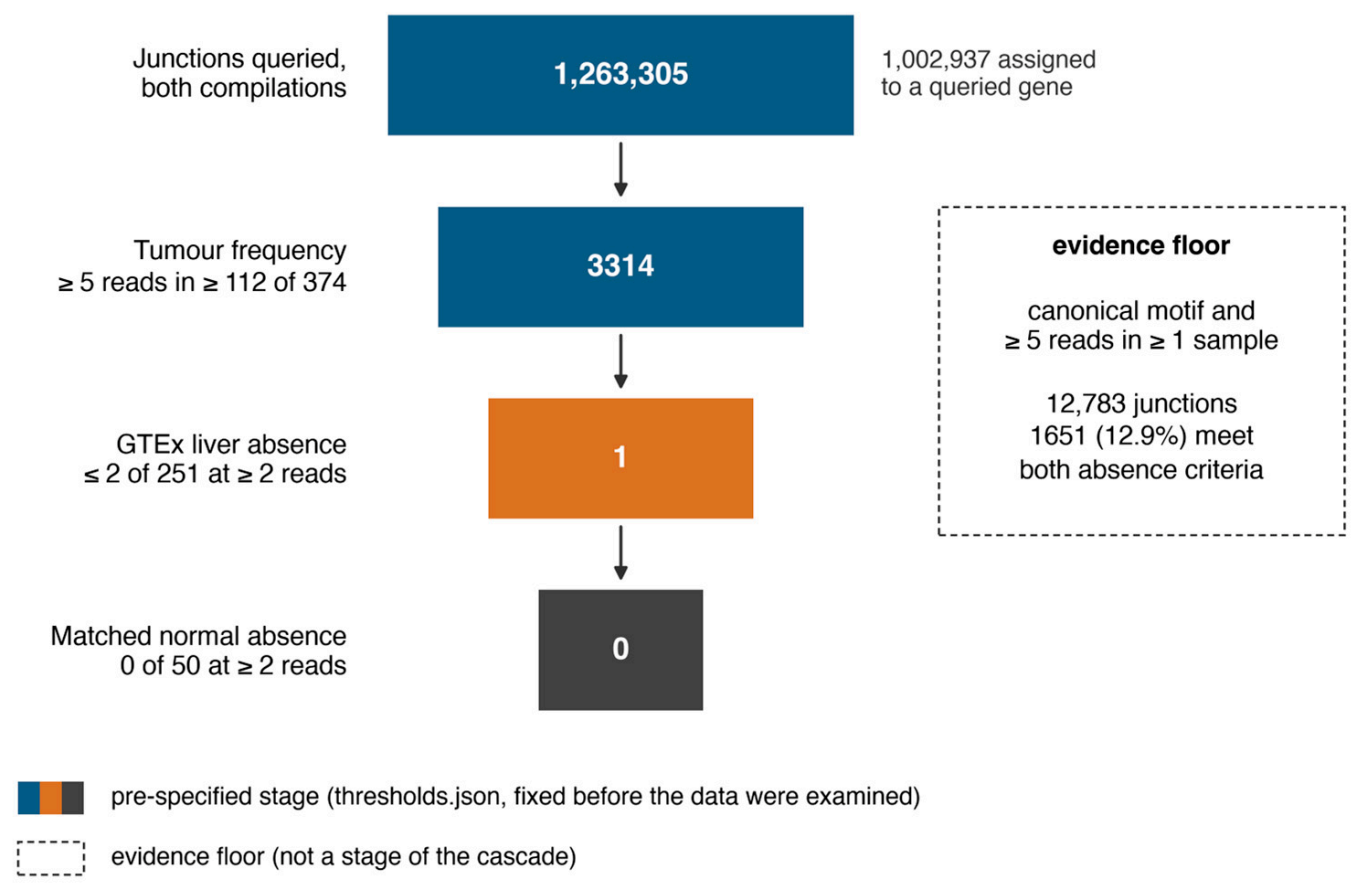
A pre-specified screening cascade in which every junction is removed, and the axis that removes them is absence from normal tissue rather than recurrence across patients. Discovery-arm cascade, from filter_attrition.tsv. The recurrence criterion is applied to all 1,263,305 junctions, and later stages are cumulative; the gene-assignment count (1,002,937) is not a stage. Thresholds were fixed in thresholds.json before the data they apply to were examined. Of the 1,263,305 junctions, 3314 meet the recurrence criterion, one of those also meets GTEx-liver absence, and none also meets matched-normal absence. At a three-read tumour floor (post hoc), one junction, the UGT2B11 to UGT2B27P readthrough (Section 3.3.2), meets all three criteria. The dashed box is the evidence floor applied separately to interpret the 98.36% raw normal-absent fraction (Section 3.1, Supplementary Figure S1) and is not a stage of the cascade.

The primary outcome is the pre-specified filtering cascade of Figure 1. Of the 1,263,305 junctions queried, 1,002,937 were assigned to a queried gene, and 3314 met the recurrence criterion alone (at least five reads in at least 112 of 374 tumours). Of those 3314, one also met GTEx-liver absence, and none also met matched-normal absence; consequently, no junction satisfied all three pre-specified criteria simultaneously.

The quantity eliminated by this cascade is not recurrence. Section 3.5 shows directly that recurrence, in isolation and without any normal-absence requirement, is readily achieved at the panel level: a single canonical, annotated junction in A2M (chr12:9,067,840–9,068,182, minus strand) reaches all 374 of 374 tumours once the normal-absence criteria are removed, because the junctions with the highest tumour prevalence are themselves expressed in normal liver. Recurrence alone is therefore not limiting; the recurrent junctions fail on normal-tissue absence. The two criteria are not independent: the junctions with the highest cross-patient prevalence arise from genes abundantly expressed in liver and are therefore present in normal tissue, whereas the junctions meeting the absence criteria are predominantly rare (Section 3.4 and Section 3.6).

Before any evidence floor is applied, 1,242,649 of 1,263,305 junctions (98.36%) already meet both normal-absence criteria. That figure reflects the denominator rather than specificity: at more than 4200 returned junctions per queried gene, the raw set is dominated by records with no carrier at two or more reads in any of the three cohorts (1,211,474 of 1,263,305; 95.9%), which meet the absence criteria by default. Restricting to the evidence floor (Section 2.6) leaves 12,783 junctions (1.01% of the raw total), of which 1651 (12.92%) meet both normal-absence criteria. Under the upper-case rule of Section 2.4, with the motif taken from either compilation, the evidence floor comprises 11,069 junctions (Supplementary Section S2 and Supplementary Table S7).

Splice-motif composition (post hoc) is consistent with that interpretation. In the normal-absent set (1,242,649 junctions), 83.5% of junctions carry canonical motifs, 1.6% minor-spliceosome motifs (AT..AC or GT..AT), and 14.9% non-canonical motifs, against 97.0%, 0.2%, and 2.8% in the annotated reference set of the same genes (7918 junctions; motifs matched without regard to letter case; Supplementary Figure S1). Recurrence figures for the eligible set reported below are therefore restricted to canonical-motif junctions (Section 2.4); the cascade counts of Figure 1 and Section 3.7 carry no motif filter.

### 3.2. The Choice of Normal Reference Changes Candidate Eligibility

Depth-normalised ALB coverage, used as a liver expression control (post hoc), is higher in TCGA-LIHC matched normal (median 314,890.1 reads per 10^6^ panel junction reads, *n* = 50) than in GTEx liver (median 185,611.2, *n* = 251; ratio of medians 1.70, Mann–Whitney *p* = 1.57 × 10^−9^). The two normal-tissue cohorts are therefore not equivalent as a reference for liver gene expression (Table 1, Supplementary Figure S2).

One unannotated junction, chr1:196,690,240–196,905,107 (plus strand, 214.9 kb), with its donor end in CFH and acceptor end in CFHR4, spanning CFHR3 and CFHR1, was present in 47 of 50 (94.0%) TCGA-LIHC matched normals and met the pre-specified GTEx-liver absence criterion, with no carrier among 251 samples. It was present in 214 of 374 (57.2%) tumours, 204 of them at the pre-specified five-read floor, and is the one junction of Section 3.1 that met both the recurrence and the GTEx-liver criteria. Because the compilations record this 214.9 kb junction only in samples carrying at least four reads (Section 3.6), the counts at the two-read definition are counts of carriers at four or more reads.

Four candidate explanations for the difference between the two normal cohorts were tested post hoc, and none accounted for it (Supplementary Section S1). Detectability does not account for it: GTEx reads are longer, and a 20% lower junction coverage than in the matched normals (Supplementary Section S1) does not account for 47 of 50 carriers against 0 of 251. Paralogue mismapping was not supported by flank alignment. A CFHR3-CFHR1 deletion haplotype was excluded because carriers of the junction (tumour and matched-normal samples pooled) have higher rather than lower coverage across the deleted interval (Mann–Whitney *p* = 9.11 × 10^−21^). Donor-side locus state does not account for the difference, since coverage of the CFH, CFHR1, CFHR2, and CFHR3 cluster does not differ significantly between the two cohorts (median 8485.2 versus 7949.4 reads per 10^6^, ratio 1.07, *p* = 0.107), in contrast to the offset observed for ALB (Table 1). The mechanism is undetermined. The junction bears directly on the design question: a discovery pipeline using GTEx alone as its normal reference would have carried it forward, and its removal depends on the patient’s own adjacent tissue.

The two references were also compared across the whole panel (post hoc; Supplementary Figure S6, Supplementary Table S11). Of the 12,783 junctions passing the evidence floor, 9011 were detected at two or more reads in at least one sample of both references, 2496 in GTEx liver only, 315 in matched normal tissue only, and 961 in neither. Detection fractions were correlated (Spearman rho = 0.839 over the 11,822 junctions detected in at least one reference; 0.916 for annotated and 0.585 for unannotated junctions). Discordance at appreciable frequency was uncommon but present in both directions: 73 junctions were detected in at least 10% of matched normal samples with no GTEx carrier, and 514 in at least 10% of GTEx samples with no matched-normal carrier; at 50%, the counts were 10 and 60. Neither direction is explained by cohort size. A junction carried by 10% of a population is missed in all of 50 samples with a probability of 0.5% (0.9^50^) and in all of 251 samples with a probability of 3.3 × 10^−12^. If each of the 7246 junctions detected in at least 10% of GTEx samples were carried by at least 10% of matched normal tissue, chance non-detection in all 50 matched normals would account for at most 37.3 of them, against 514 observed, and the corresponding expectation in the converse direction is below 10^−7^. The longer reads of GTEx libraries are a candidate contributor to the GTEx-only direction. The CFH to CFHR4 junction is one of the 10 junctions carried by at least half of the matched normal samples and by no GTEx sample. Genome-wide, 335,637 junctions passed the evidence floor (Supplementary Table S15, Supplementary Figure S7)—238,915 were detected in both references, 64,062 in GTEx liver only, 2590 in matched normal tissue only, and 30,070 in neither—and detection fractions were correlated with Spearman rho = 0.898 (0.919 for annotated and 0.466 for unannotated junctions; 0.847 for the panel under the same definition). Discordance at appreciable frequency was present in both directions genome-wide as well: 245 junctions were detected in at least 10% of matched normal samples with no GTEx carrier and 12,154 in at least 10% of GTEx samples with no matched-normal carrier, and 21 and 1043 at 50%. Chance non-detection would account for at most 1074.5 of the latter (208,489 junctions detected in at least 10% of GTEx samples, multiplied by 0.9^50^) and for fewer than 10^−6^ of the former. The panel-wide pattern therefore holds across the genome.

### 3.3. Recurrent Eligible Junctions Reflect Expression Differences and Readthrough Events

**Figure 2:**
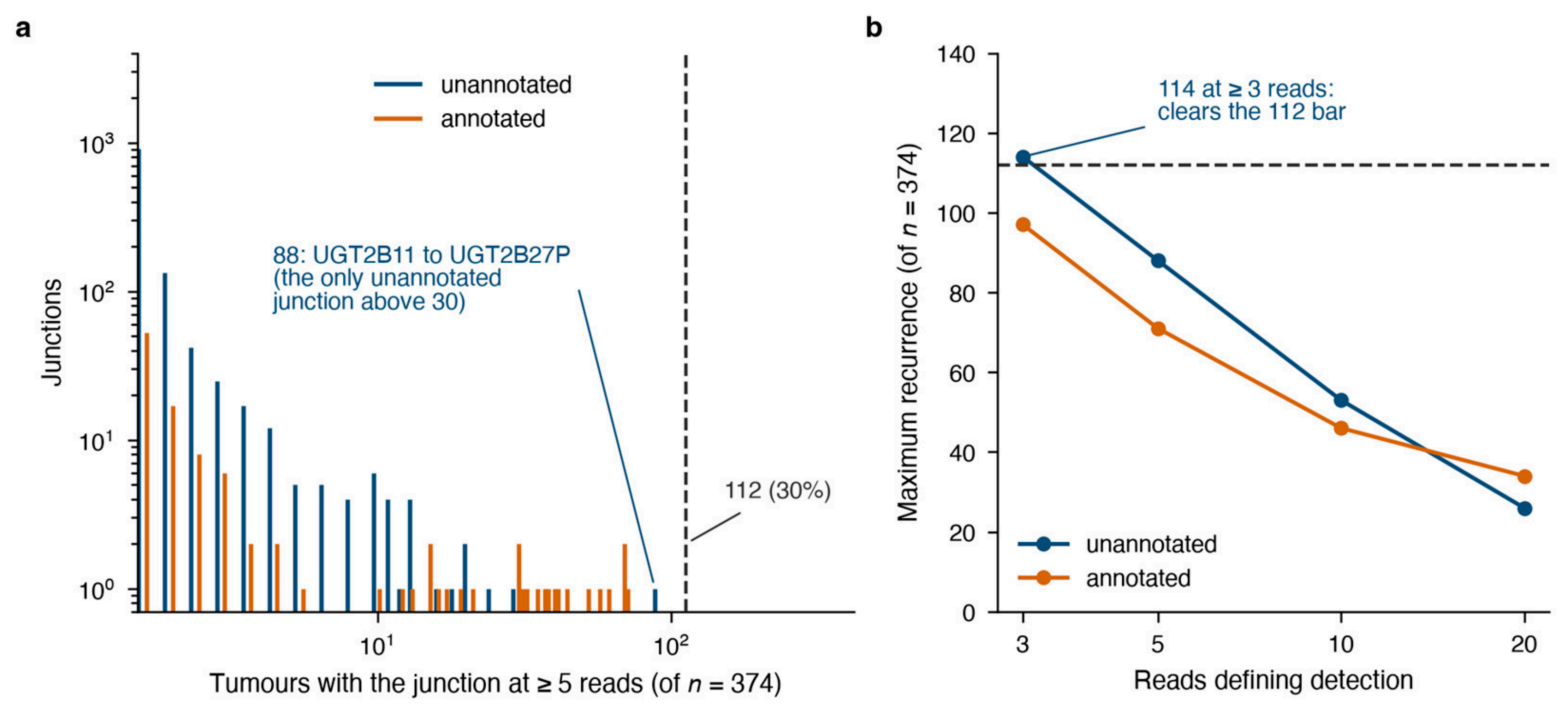
Recurrence of normal-absent junctions depends on the read threshold, and unannotated normal-absent junctions are mostly private to single patients. (**a**) Recurrence spectrum of canonical, normal-absent junctions from recurrence_spectrum_by_annotation.tsv, split by GENCODE v26 annotation status and computed on the case-insensitive canonical set of 1292 junctions (1178 unannotated, 114 annotated; Supplementary Section S2), as is panel (**b**). Each vertical line is the number of junctions detected in a given number of tumours (*n* = 374). Every count in this panel is conditioned on the pre-specified detection threshold of five reads, which is stated on the x axis because the entire spectrum shifts with it (panel (**b**)). Both axes are logarithmic, and the x axis is linear below 10; the dashed line is the pre-specified criterion of 112 of 374. The single unannotated junction above 30 tumours is marked: the UGT2B11 to UGT2B27P readthrough characterised in Section 3.3.2. (**b**) Maximum recurrence against the read threshold defining detection, from threshold_sweep.tsv, computed separately for annotated and unannotated junctions (post hoc analysis). The dashed line is the pre-specified criterion of 112 of 374, as in (**a**). The marked point is the UGT2B11 to UGT2B27P junction, which exceeds the 112-tumour floor at three reads (Section 3.3.2). Counts are raw Snaptron read counts.

The eligible set (Section 2.6) comprises 981 junctions: 894 unannotated and 87 annotated in GENCODE v26 (Figure 2a shows the corresponding case-insensitive set of 1292; Supplementary Section S2).

The two subsets behave differently. Unannotated junctions have a median recurrence of 1 of 374 tumours and a maximum of 88 of 374 (23.5%). That maximum rests on a single junction, addressed in Section 3.3.2; the next most recurrent unannotated junction reaches 29 of 374 (7.8%), and the unannotated ceiling is therefore 88 of 374 with that junction included and 29 of 374 without it. Annotated junctions have a median recurrence of 2 of 374 and a maximum of 71 of 374. Neither subset reaches the pre-specified recurrence floor of at least 112 of 374.

Above 30 of 374 tumours, the recurrence spectrum holds 15 junctions, and 14 of them are annotated. Twelve map to the TERT locus and are addressed in Section 3.3.1. Two map to the UGT1A locus on chromosome 2 (UGT1A10 to UGT1A1, 61 of 374; UGT1A8 to UGT1A1, 44 of 374). Both are annotated junctions that run from a first exon of one UGT1A entry onto the shared downstream exons of the locus: the nine UGT1A protein-coding entries share a single 3′ end and differ only in which alternative first exon is used. A junction of this form therefore reports which first exon is active rather than a splicing event of a class absent from normal tissue. UGT1A8 and UGT1A10 are extrahepatic first exons, and their presence in tumours and absence from matched normal liver are consistent with a shift in first-exon usage. Across GTEx, both junctions are carried predominantly by small intestine (145 of 193 samples for the UGT1A10 junction and 127 of 193 for the UGT1A8 junction), colon, and stomach samples, and by no liver or whole-blood sample (post hoc; Supplementary Table S9); this tissue pattern matches extrahepatic first-exon use. The fifteenth junction is the only unannotated one and is addressed separately in Section 3.3.2. Every junction above this threshold therefore lies at one of three loci, and none is a novel tumour-specific splice event.

As a post hoc check, the same unfiltered method and the same criteria were applied to GNAS and RPL22, two genes for which recurrent tumour-wide neojunctions have been reported [18]. GNAS is a member of the panel, as a predicted membrane protein, and its junctions were therefore also screened in the pre-specified cascade; RPL22 is not secreted or membrane-bound and lies outside the panel. The query returned 12,431 junctions, of which 18 met the recurrence criterion, all of them annotated; none met either absence criterion, and the smallest matched-normal burden among the 18 was 43 of 50 samples. The source study gives no coordinates for its neojunctions and describes them as alternative 3′ splice-site events that remove two nucleotides in GNAS and six in RPL22 [18]. Junctions matching those descriptions are present in the retrieved set (Supplementary Section S5.1): one in GNAS (chr20:58,910,402–58,910,684), carried at two or more reads by 69 of 374 tumours, 3 of 50 matched normal samples, and 27 of 251 GTEx liver samples, and two in RPL22, carried by 15 and 8 tumours and by 1 matched normal sample each; at the five-read tumour floor, the three are carried by 3, 1, and 0 tumours. They are identified by description rather than by published coordinates, and none meets the absence criteria; the query is not a positive control.

As a positive control (post hoc; Section 2.9, Supplementary Table S8), two established oncogenic junctions were retrieved from the same compilations by exact-coordinate query and counted at the same read floors, in the cohorts in which they occur. The EGFRvIII junction (EGFR exon 1 to exon 8; chr7:55,019,366–55,155,829) was detected at five or more reads in 30 of 170 TCGA glioblastoma tumours (17.6%), in 0 of 5 glioblastoma solid-tissue normal samples, and in 0 of 2931 GTEx brain samples, while both flanking junctions were detected in normal brain (exon 7 to exon 8 in 1604 of 2931 GTEx brain samples). The EGFRvIII intron is 136 kb, for which only samples with at least three reads are recorded (Section 3.6), and the junction was not returned by the GTEx compilation for any tissue. MET exon 14 skipping (exon 13 to exon 15; chr7:116,771,655–116,774,880) was detected in 15 of 542 TCGA lung adenocarcinoma tumours (2.8%), in 0 of 59 solid-tissue normal samples, and in 1 of 655 GTEx lung samples at two reads, with both flanking junctions present in all 59 normal samples. The retrieval and the thresholds therefore recover known oncogenic junctions and return them as absent from the corresponding normal references under the criteria of this study (no matched-normal carrier, and GTEx carriers within the 1% tolerance). Both junctions (17.6% and 2.8% of tumours) lie below the 30% recurrence floor. For EGFRvIII, the five matched normal samples carry almost no evidential weight, whereas the 2931 GTEx samples do, which illustrates the dependence on reference size examined in Section 3.4.

#### 3.3.1. Recurrent Annotated Junctions at TERT Reflect Expression, Not Splicing

Fifteen canonical-motif (CT..AC), normal-absent junctions in the TERT window (chr5:1,253,147–1,295,086, minus strand) reach at least 10 of 374 tumours at the pre-specified five-read threshold, up to a maximum of 71 of 374. All 15 are annotated in GENCODE v26. Thirteen join consecutive exons of the principal TERT transcript and are therefore the ordinary junction ladder of full-length TERT rather than a distinct splicing event. Of the remaining two, one (chr5:1,268,634–1,278,640, 30 of 374) skips exons 7–8, which is consistent with the TERT beta-deletion variant quantified in earlier work [52]; the other (chr5:1,254,665–1,255,286, 21 of 374) has a boundary that does not coincide with a principal-transcript exon edge, although it is itself annotated in GENCODE v26.

A sixteenth junction in the same window, chr5:1,253,832–1,254,367 (exon 16 to exon 15, 79 of 374 tumours, 0 of 50 matched normals, 1 of 251 GTEx liver), meets both normal-absence criteria and is annotated, but its donor-acceptor motif is CT..GC, which on the minus strand corresponds to a GC-AG donor rather than the canonical GT-AG: a real but non-canonical splice site. Under the motif-based definition of canonical applied throughout this report (Section 2.4), it is therefore reported here as a separate observation and is not counted toward the maximum recurrence, carrier count or depth statistics of the 15-junction set.

**Figure 3:**
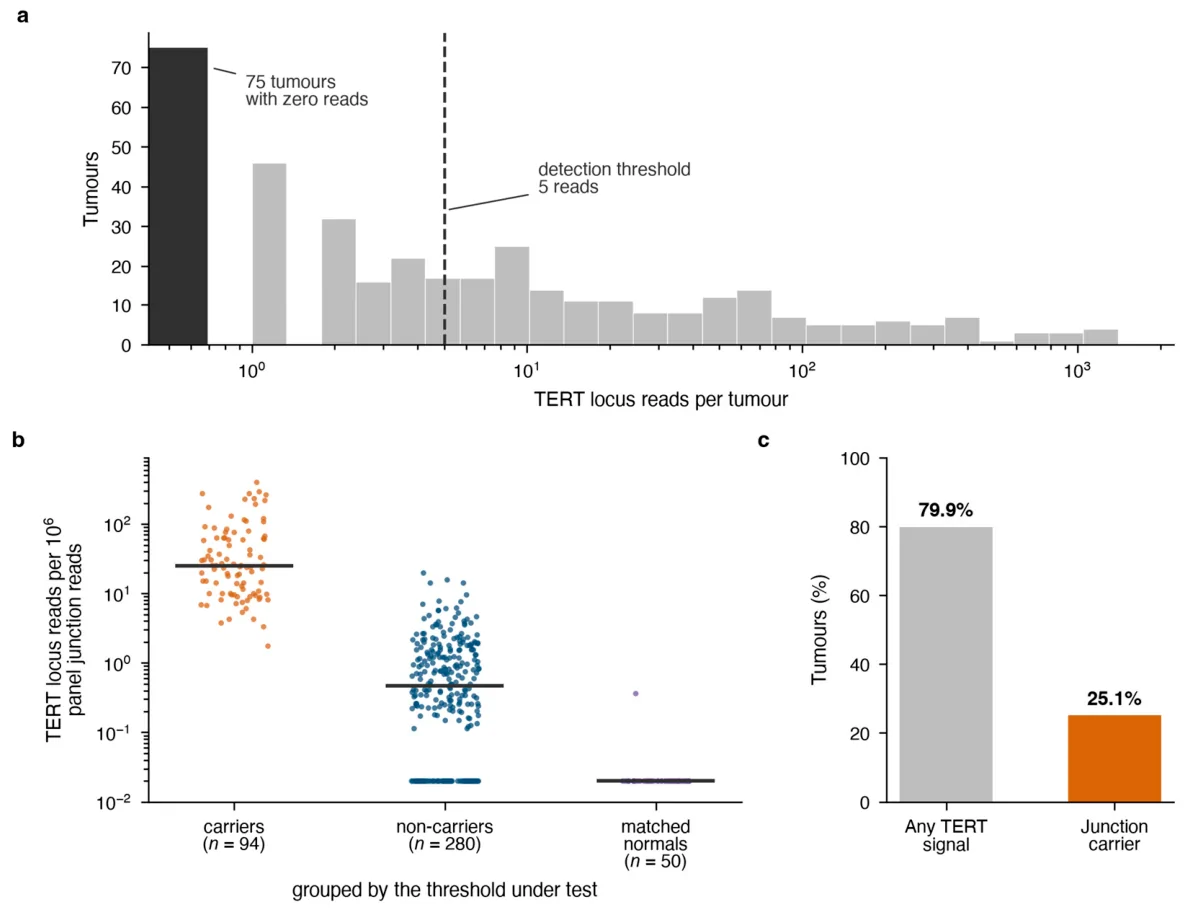
TERT restriction to tumours is a gene-expression difference read through a fixed threshold, not tumour-specific splicing. (**a**) Distribution of raw TERT locus junction reads across all 374 tumours, ungrouped, from tert_expression.tsv. Bins are logarithmic; the 75 tumours with zero reads are drawn as the separate leftmost bar in dark grey. The dashed line is the pre-specified five-read threshold, across which the distribution declines smoothly with no gap. (**b**) The same data grouped as carriers against non-carriers, with matched normals for comparison; points are jittered, and bars are group medians. Panel (**a**) is in raw reads, the unit in which the threshold is defined, and panel (**b**) is depth-normalised. Panel (**b**) is descriptive only, because the grouping variable is derived from reads at the same locus; samples with zero TERT reads are plotted at 0.02 to remain visible on the logarithmic axis. (**c**) Proportion of tumours with any TERT locus signal against the proportion meeting the junction-level carrier definition; the two thresholds are distinct, and both are quantified in the text. Post hoc analysis.

Carriers of these 15 junctions have more TERT locus junction reads than non-carriers (medians 24.9 versus 0.5 reads per 10^6^ panel junction reads; Figure 3b), and the 50 matched normals have a median coverage of 0.0. Because carrier status is defined from junction reads at the same locus, the comparison is descriptive and is not independent evidence (Section 2.12). The underlying distribution is continuous rather than bimodal: raw TERT locus read counts fall smoothly across the pre-specified threshold, with 16, 22, 17, 11, and 6 of the 374 tumours carrying exactly three, four, five, six, and seven reads. The cohort median is four reads, one read below the pre-specified five-read threshold (Figure 3a). In all, 299 of 374 tumours (79.9%) carry some detectable TERT locus signal, while only 94 of 374 (25.1%) cross the five-read junction-carrier threshold used to define a carrier, that is, at least five reads on at least one of the 15 recurrent TERT junctions (Figure 3c). A related but distinct quantity, the number of tumours reaching five reads summed across the whole TERT locus rather than on any single junction, is 183 of 374 (48.9%). Of the 280 non-carriers, 62 exceed 1.76 reads per 10^6^ panel junction reads, the lowest depth-normalised value among the 94 carriers; the carrier/non-carrier boundary therefore lies within a continuous tail rather than at a natural gap. This is not a splicing difference between two discrete states; it is a read threshold imposed on a continuous, low-abundance expression tail of a locus that is largely silent in normal liver. The locus is not silent elsewhere: across GTEx, the five absence-supported TERT junctions are carried by 263 to 392 of 410 testis samples and are detected at two or more reads in 11 to 45 of 852 whole-blood samples (post hoc; Supplementary Table S9).

#### 3.3.2. A Single Long-Range Readthrough Junction at the UGT2B Locus

**Figure 4:**
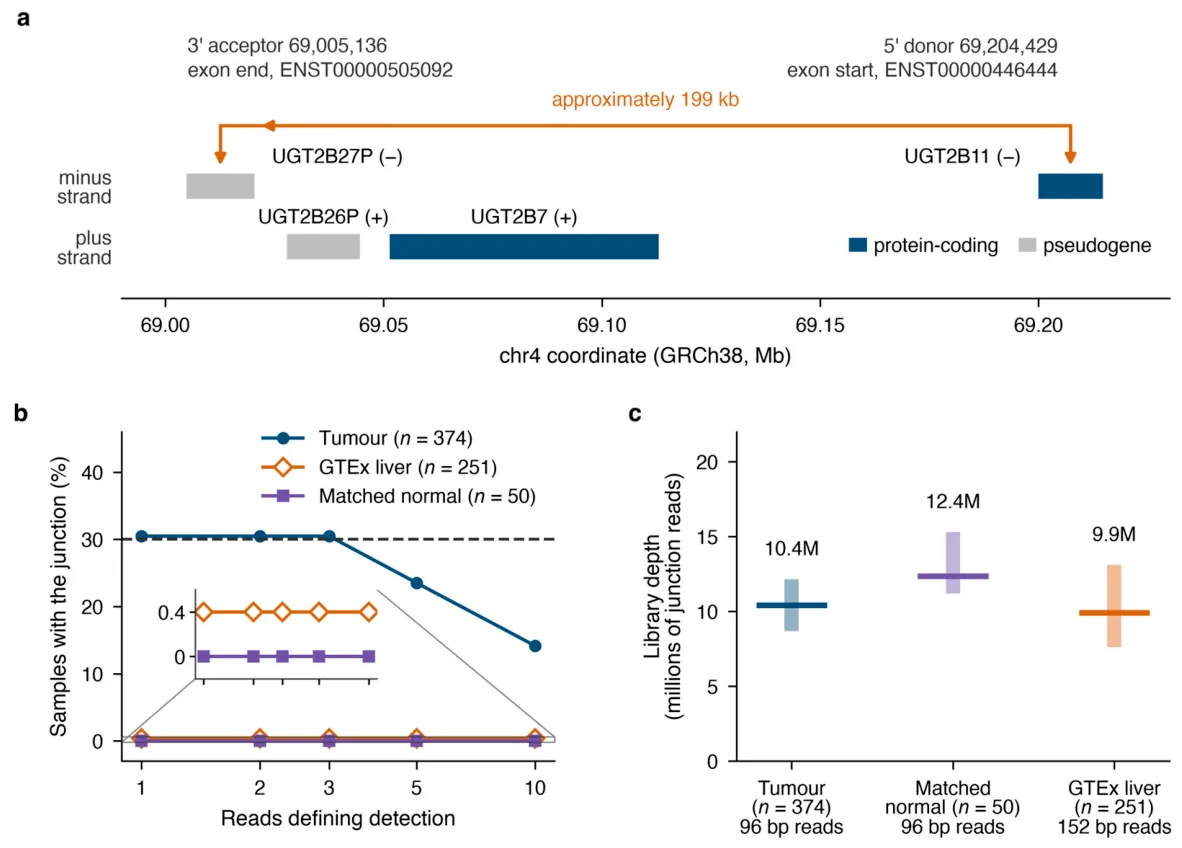
The one junction that would meet the criteria at a three-read tumour floor joins UGT2B11 to UGT2B27P, and its absence from normal liver is not explained by library depth. (**a**) The chr4 UGT2B locus on GRCh38, drawn from GENCODE v26; fill is dark for protein-coding genes and light for pseudogenes, and genes are placed on the row matching their strand. The junction runs on the minus strand; its 5′ partner is therefore the gene at the higher coordinate. Both intron ends coincide exactly with annotated exon boundaries and are therefore marked; transcript identifiers are given in the text. The junction itself is unannotated, because no GENCODE v26 transcript spans both genes. (**b**) Detection frequency by cohort and read threshold, from chr4_junction_counts.tsv; the dashed line is the 30% recurrence criterion, and the inset magnifies the range from 0 to 0.6%, which separates the GTEx liver and matched-normal series. Counts below three reads are not recorded for an intron of this length, and the values at one and two reads therefore equal those at three reads. (**c**) Library depth by cohort from cohort_depth.tsv, as junction_coverage from the Snaptron bulk metadata; bars are medians, shading the interquartile range, and median read length is on the axis. The inference this panel supports is that no cohort lacking the junction is technically disadvantaged: the matched normals, in which the junction never appears, are the deepest of the three cohorts. Post hoc analysis.

One unannotated junction departs from the pattern described in Section 3.3. Its coordinates are chr4:69,005,136–69,204,429, minus strand, an intron of 199.3 kb. Its 5′ partner is therefore the gene at the higher coordinate: the transcriptional label is UGT2B11 to UGT2B27P, a readthrough from a protein-coding gene into a downstream pseudogene within the chr4 UGT2B tandem array. The junction itself is unannotated in GENCODE v26, but its two individual splice sites are not cryptic: each is independently an annotated exon boundary in that same annotation. The donor at position 69,204,429 coincides with the exon boundary of the UGT2B11 transcript ENST00000446444, and the acceptor at position 69,005,136 with the exon boundary of the UGT2B27P transcript ENST00000505092, giving two exact boundary matches, one at each end (Section 2.8, Figure 4a).

At the pre-specified five-read tumour threshold, this junction reaches 88 of 374 tumours (23.5%), below the required 112 (Figure 4b). At a three-read threshold, it reaches 114 of 374 (30.5%), at or above the 112 required, while both normal-absence criteria remain met at every threshold from 3 to 10 reads: 0 of 50 matched normals and 1 of 251 GTEx liver samples. Counts below three reads are not recorded for an intron of this length (Section 3.6), and three reads is therefore the lowest threshold that can be evaluated. This is the only junction in the panel for which the recorded pre-specified result changes between the three-read and five-read tumour signal minimum.

Two alignment-artefact explanations were tested directly, post hoc. First, both 100 bp flanks of the junction align to exactly one genomic location at zero mismatches, with no other alignment within five mismatches, which is inconsistent with paralogue mismapping. Second, depth-normalised UGT2B locus coverage (chr4:68.5–69.7 Mb) does not differ significantly between tumour (median 1374.9 reads per 10^6^ panel junction reads, *n* = 374) and matched normal (median 1383.7, *n* = 50; ratio of medians, normal to tumour, 1.01, Mann–Whitney *p* = 0.787). A mismapping artefact driven by locus expression would instead be expected to produce higher coverage in normal liver; UGT protein levels are reported to be lower in HCC than in matched non-tumour liver [53]. An independent query of the Snaptron srav3h compilation (heterogeneous human SRA) returns the identical junction coordinates, with a canonical CT..AC minus-strand motif, in 1023 of 316,447 samples (0.32%) from 215 studies, indicating that the junction recurs outside this cohort. Because the same primary assembly underlies all of these alignments, this does not distinguish a genuine transcript from a systematic alignment product. A keyword search of the free-text metadata of that compilation (Section 2.10) assigns 321 of the 1023 carriers to liver-related samples and 1 to blood-related samples and leaves 701 unassigned, and the two studies contributing most carriers are of prostate cancer (203 and 75 carriers).

In a post hoc comparison within the tumour group, carriers of this junction at five or more reads (*n* = 88) have modestly higher sequencing depth than non-carriers (median library junction coverage 11.22 million versus 10.16 million reads, Mann–Whitney *p* = 1.01 × 10^−3^). If matched normal tissue carried the junction at its tumour prevalence of 114 of 374 (30.5%) at the three-read threshold, the probability of observing zero carriers among 50 matched normals would be 1.27 × 10^−8^. The 10.4% depth difference between five-read carriers and non-carriers within tumours is too small to produce that contrast, and the matched normals are the deeper cohort.

Of the 12 junctions in the paired comparison set out below (Table 2), this one has the largest number of pairs: 15 patients whose tumour carries the junction have a matched normal.

Four further post hoc observations bear on the origin of this junction (Supplementary Tables S10 and S14). First, it is specific neither to HCC nor to tumour tissue. Across TCGA, it is carried at three or more reads by 161 of 1142 breast tumours and by 33 of 114 breast solid-tissue normal samples, and outside liver and breast by at most 8 samples in any project. Across GTEx it is carried by 11 of 19,214 samples in five tissues (5 of 482 breast, 2 of 706 thyroid, and 1 of 251 liver among them) and by none of the 852 whole-blood samples. The junction is therefore also present in normal breast and is not confined to tumours. Second, the two splice sites lie within a pair of segmental duplications that are each other’s partners (chr4:69,004,421–69,023,443 and chr4:69,200,001–69,217,694; 92.3% sequence identity), as expected for two members of a tandem gene array. The uniqueness of the 100 bp flanks within five mismatches (above) argues against simple mismapping between the two copies; a junction arising from a haplotype absent from the primary assembly remains possible: an alternate-haplotype sequence (chr4_GL000257v2_alt) covers the acceptor side, and 105 structural variants recorded in the Database of Genomic Variants overlap the junction interval. Third, UGT2B17 carries a well-documented deletion polymorphism [54,55], a 117 kb deletion mediated by flanking segmental duplications [56]; the gene lies 378 kb below the junction interval, and that deletion therefore does not span either splice site. UGT2B28 lies 76 kb above the interval and overlaps neither splice site; its common deletion [57] is visible in the blood-derived normal DNA of the same patients as a focal loss of median 104 kb in 77 of 306 samples (25.2%), ends a median 57.5 kb from the junction interval, and does not differ significantly in frequency between carriers and non-carriers (27.8% of carriers against 23.8% of non-carriers among 304 patients, *p* = 0.47; Supplementary Table S14). Fourth, the junction is not associated with a copy-number change in the interval between its splice sites (Supplementary Table S14). In SNP-array copy-number segments, the log2 ratio of the interval did not differ significantly between carriers and non-carriers in tumour DNA (median −0.042 against −0.053, *p* = 0.63; values below −0.3 in 23.0% against 23.8%), in blood-derived normal DNA (0.007 against 0.005, *p* = 0.19; values below −0.3 in 0 of 90 against 2 of 214), or in solid-tissue normal DNA (0.015 against 0.008, *p* = 0.20). Tumour segment breakpoints within the interval did not differ significantly in frequency (9.7% against 10.5%, *p* = 1.00). The same data show the UGT2B17 germline deletion in 107 of 306 blood-derived normal samples (35.0%); a deletion of comparable size at the junction interval would therefore have been detected. A germline or somatic deletion joining the two genes is consequently not supported.

No protein product is expected from a readthrough transcript into a pseudogene, and the junction is present in normal breast tissue; it is therefore not a tumour-specific splice variant in the sense used in this study, and its origin is unresolved.

For the 12 most recurrent canonical, normal-absent junctions in the panel, each tumour carrier was also paired post hoc with the same patient’s matched normal. The pairing records, for each junction, the number of tumour carriers with their own normal available, and therefore the weight that the zero carries; because the junctions were selected for zero matched-normal carriers, it is not independent of that selection. The set is defined by rank, and its lowest member reaches 37 of 374 tumours. All 50 matched normals in the cohort pair to a tumour sample, and each of the 12 junctions has at least three paired carriers (Table 2, Supplementary Figure S3).

The weight of the zero differs by junction: 4 of 12 rest on eight or more paired carriers (null probability at most 0.4%), while 4 of 12 rest on four or fewer paired carriers (null probability at least 6.3%).

### 3.4. Evidence for Normal-Tissue Absence Is Limited for Rare Junctions

The 981 eligible junctions are defined in part by absence from normal tissue: 0 of 50 matched normals and at most 2 of 251 GTEx liver samples at two reads. The evidential weight of those observations was examined post hoc. Two regimes must be distinguished. Junctions carried by few tumours fail the single-target recurrence criterion by definition, irrespective of their status in normal tissue, and the power of the absence filter is not the reason the primary criterion fails for them; it is the reason their inclusion in a union panel (Section 3.5) rests on assumed rather than demonstrated absence. Junctions prevalent enough for absence to be demonstrable are few; the eight supported by the matched-normal criterion are examined in Section 3.3.

**Figure 5:**
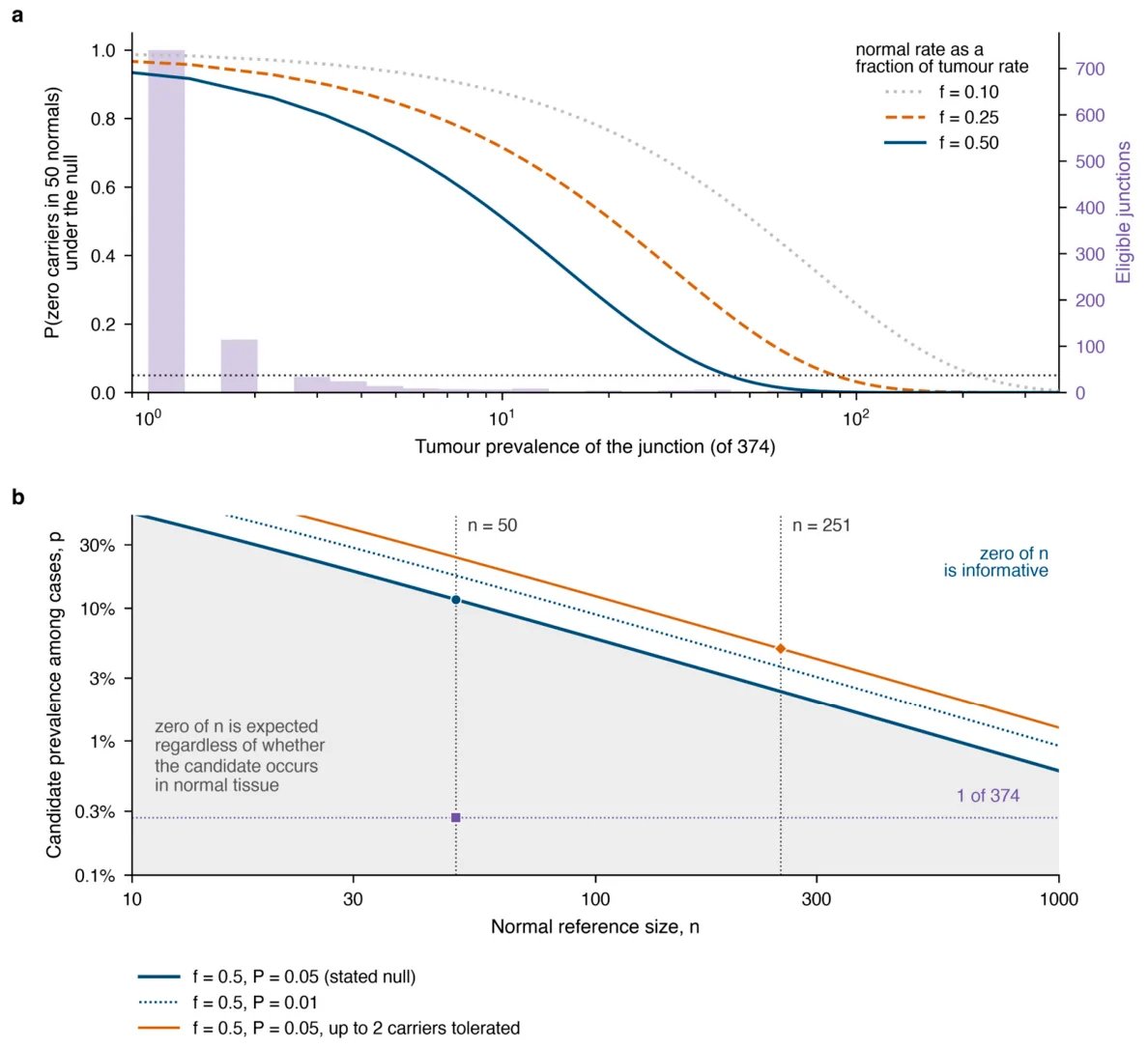
The absence filters are informative only above a candidate prevalence that is set by the size of the normal reference. (**a**) Probability of observing zero carriers among the 50 matched normals under the null that a junction also occurs in normal liver at a fraction *f* of its tumour prevalence, (1 − *f*·*p*)^50^, for *f* = 0.10, 0.25, and 0.50; the dotted line is *p* = 0.05. The purple histogram on the right axis is the tumour-prevalence distribution of the 981 eligible junctions, from eligible_absence_evidence.tsv. The two are plotted together because the informative feature is their position relative to each other: the eligible set is concentrated at the low-prevalence end, where all three curves approach 1 and zero-of-50 is expected regardless of whether the junction occurs in normal liver. (**b**) Minimum candidate prevalence at which an absence criterion on n normals is informative under the stated null *f* = 0.5. Navy curves are for a zero-observation criterion, *p_min_* = (1 − alpha^1/n^)/*f*, at *p* = 0.05 (solid) and *p* = 0.01 (dotted); the orange curve is for a criterion tolerating up to two carriers at *p* = 0.05, computed from the binomial distribution. The shaded region is where zero of n is expected irrespective of whether the candidate occurs in normal tissue. Vertical lines mark the two references used in this study. The circle is the operating point for matched normal tissue (*n* = 50, zero carriers, *p* = 11.63%, at least 44 of 374 tumours); the diamond is the GTEx criterion (*n* = 251, up to two carriers, *p* = 4.97%, at least 19 of 374); the square is the median eligible junction (1 of 374; 740 of the 981 eligible junctions are at this prevalence). The panel is closed-form and reads no data file. Post hoc analysis.

The eligible set is dominated by rare junctions: median tumour prevalence is 1 of 374 (IQR 1–1), and 740 of 981 (75.4%) are carried by a single tumour. Under a null model in which a junction also occurs in normal liver at a fraction *f* of its tumour prevalence *p*, the probability of observing zero carriers among 50 matched normals is (1 − *f*·*p*)^50^. At *f* = 0.10, 951.5 of 981 junctions (97.0%) would be expected to pass the zero-carrier filter by chance; at *f* = 0.25, 916.0 (93.4%); at *f* = 0.50, the most detectable null tested, 867.7 (88.5%) would still pass by chance, and only 8 of 981 junctions have a P(zero) below 0.05 under this null (Table 3, Figure 5a).

Two measurement asymmetries mean these figures are bounds rather than point estimates, and both run in the same direction. Tumour prevalence *p* is measured at a five-read threshold, but a junction counts as present in a matched normal at two reads, a lower and therefore less stringent threshold. The matched normals are 18.5% deeper than the tumours in library junction coverage (median 12.36 versus 10.43 million junction reads), while the GTEx liver libraries carry a median read length of 152 bp against 96 bp in the TCGA cohorts. Both make a junction easier to detect on the normal side than on the tumour side. The percentages above are therefore an upper bound on the number of junctions that could pass the matched-normal filter by chance, and the count of junctions with statistically demonstrable absence is a lower bound: the true number is at least eight and may be higher.

The rule for calling absence supported was set before the per-junction values were computed: (1 − 0.5*p*)^50^ < 0.05, which implies a tumour-prevalence threshold of *p* > 11.63% (at least 44 of 374). Of the 981 eligible junctions, 8 meet it, 7 annotated and 1 unannotated (Table 3). The label is conditional on the stated null: these eight junctions permit rejection of a normal-to-tumour prevalence ratio of 0.5, but they are not a demonstration of absence across a normal population. Model-free, the evidence a zero observation carries is bounded by the sample size alone: 0 carriers in 50 normals is consistent with a normal-tissue frequency of up to 5.8% at 95% confidence, and 0 in the 15 paired normals of Table 2 with up to 18.1%. Figure 5b places this threshold on the general curve: for a normal reference of size *n* and an assumed normal-to-tumour prevalence ratio *f*, a zero observation is informative at level alpha only above *p*_min_ = (1 − alpha^1/*n*^)/*f*. Under the stated null, the 11.63% figure is the *n* = 50 point on that curve; the threshold falls to 3% at 199 normals and to 1% at 598, and a zero observation becomes informative for a junction at the median eligible prevalence of 1 of 374 at 2240 normals.

The same calculation was applied to the GTEx criterion and to the two references jointly (Section 2.10, Table 3, Figure 5b). The GTEx criterion tolerates up to 2 carriers of 251 and is not a zero-observation test; under the same null, it is informative above a tumour prevalence of 4.97% (at least 19 of 374), which 22 of the 981 eligible junctions reach. With the two references treated as samples of one normal population, the joint criterion is informative above 3.88% (at least 15 of 374), which 25 of 981 reach. The 14 junctions added by the GTEx criterion comprise nine further members of the TERT junction set, three at the IGF2 locus (one of them a long-range junction from LSP1), one within TF, and one joining MTA1 to IGHG1; the joint criterion adds one further TERT junction, one at CDKN2A and one in the HLA-F region. Under every criterion, more than 97% of the eligible set (956 to 973 of 981) lies below the informative prevalence.

These eight supported junctions are not novel tumour-specific splice events in their own right. Seven are annotated in GENCODE v26; the eighth, the UGT2B11 to UGT2B27P readthrough, is unannotated as a junction, although each of its two splice sites is individually an annotated exon boundary (Section 3.3.2). All eight fall in three loci already characterised elsewhere in this report: five TERT junctions (Section 3.3.1, an expression-threshold effect rather than tumour-specific splicing), two UGT1A first-exon junctions (Section 3.3), and the UGT2B11 to UGT2B27P readthrough (Section 3.3.2). The junctions whose absence is supported by the matched-normal criterion are therefore those already accounted for by expression differences or by transcripts present in other normal tissues, not by a novel, tumour-specific splicing event.

Two-cohort agreement provides a partial check independent of the power calculation: 726 of the 981 eligible junctions (74.0%) are absent from both matched normals and GTEx liver at the two-read criterion. Of those, 533 have no recorded GTEx liver read, and 193 are recorded there only in samples carrying one read; for introns longer than 50 kb, counts below two to four reads are not recorded (Section 3.6). The remaining 255 junctions (26.0%) have one or two GTEx liver carriers at two reads and rest on matched-normal absence alone.

### 3.5. Multi-Target Coverage Depends on Panel Size and Normal-Reference Criteria

**Figure 6:**
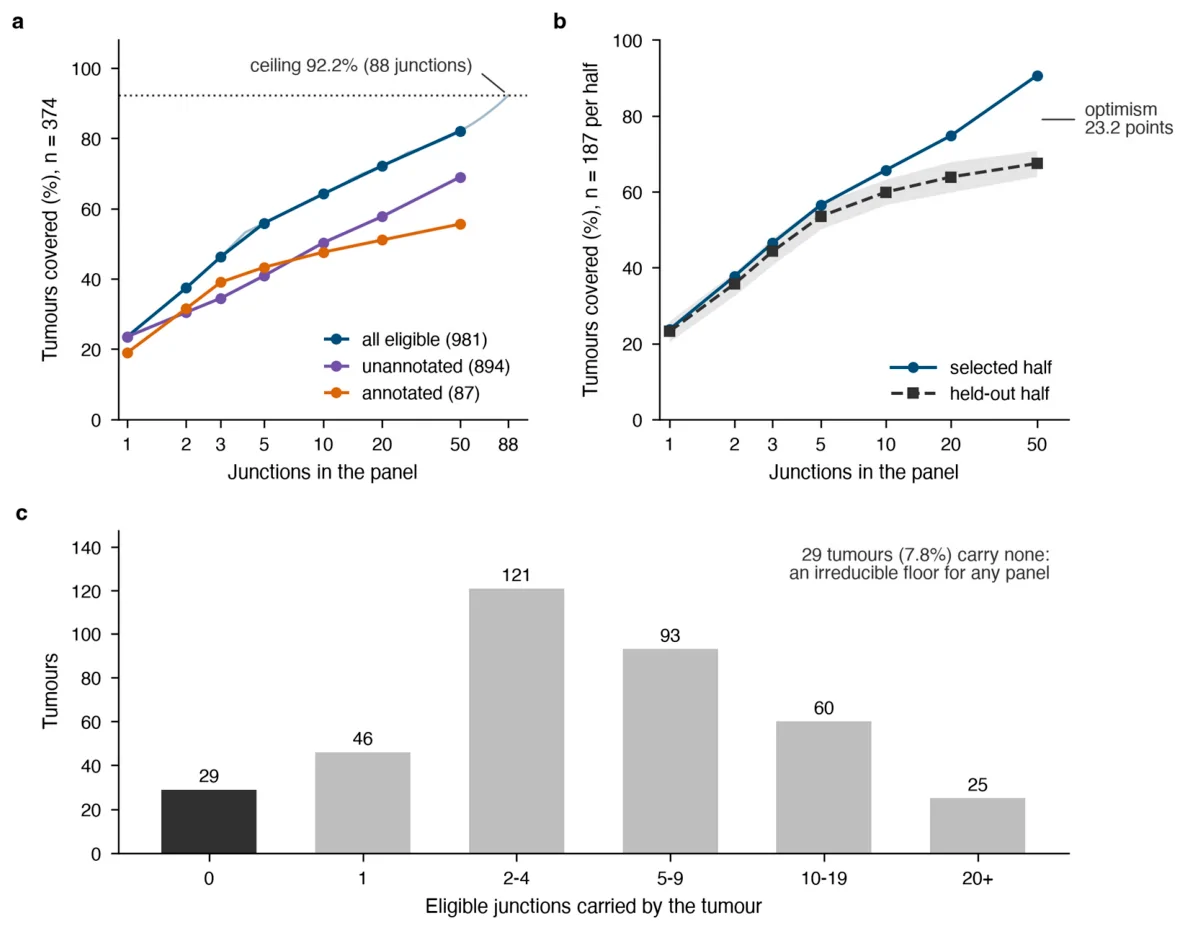
A single-target panel is excluded, but a multi-target panel is not: held-out coverage reaches 63.9% with 20 targets and 67.5% with 50, with 7.8% of tumours carrying no eligible target. (**a**) Greedy maximum-coverage curves on the full cohort of 374 TCGA-LIHC tumours, from set_cover_curves.tsv and set_cover_saturation.tsv. A tumour counts as covered when a junction reaches the pre-specified floor of five reads in it, and panel coverage is the union of its targets’ tumour sets. The three curves show the eligible set and its two annotation subsets. The dotted line is the saturation ceiling. Without the normal-tissue filter, a single canonical junction covers all 374 tumours; recurrence alone is therefore uninformative without a normal-tissue requirement. (**b**) Repeated split-half validation, from set_cover_validation.tsv: 50 random splits (seed 20260830), selected greedily on one half and scored on the other; both curves are therefore on 187 tumours and directly comparable. Points are means, and the band is one standard deviation of the held-out fraction. (**c**) Eligible junctions carried per tumour, from set_cover_per_patient.tsv. The leftmost bar is the tumours carrying no eligible junction, which no panel drawn from this set can cover. Post hoc analysis.

The pre-specified criterion was evaluated one junction at a time. A diagnostic panel, however, functions as a union of several targets: a tumour is detected if it carries any one of the panel’s junctions at the pre-specified five-read floor. The union is the clinically relevant quantity for a panel; it was not pre-specified and was examined post hoc by greedy maximum-coverage set cover. Greedy selection carries a (1 − 1/e) guarantee [50,51]; each curve is a lower bound on the coverage an optimal panel of the same size could reach within this cohort, and the guarantee bounds how far below that optimum it can lie (Figure 6a). The curves below are achievable coverage within TCGA-LIHC, not an estimate of clinical sensitivity.

Without any requirement of absence from normal tissue, the union question is readily resolved: among 8624 canonical junctions detected in at least one tumour at the pre-specified signal floor, a single junction covers 374 of 374 tumours (100%), and adding further junctions adds no coverage. That junction is chr12:9,067,840–9,068,182 on the minus strand, annotated in GENCODE v26 and carrying a canonical CT..AC motif. It falls within A2M, a secreted-protein gene ranked 65th of 150 by liver expression in the panel selection (Section 2.3). This illustrates the reason that recurrence alone is uninformative in this cohort: at the five-read threshold, it is present in 374 of 374 tumours, but also in 50 of 50 matched adjacent normals and 251 of 251 GTEx liver samples, and it therefore fails both absence criteria as completely as it satisfies the recurrence criterion.

Restricting to the eligible set of 981 junctions, coverage rises with panel size but considerably more slowly (Table 4, Figure 6a). Run to exhaustion rather than stopped at 50 targets, the eligible set’s coverage ceiling is 345 of 374 tumours (92.2%), reached at 88 junctions; 95% coverage is not reachable from this set at any panel size. Restricted to the 87 annotated eligible junctions alone, coverage plateaus at 208 of 374 (55.6%) using 37 junctions; restricted to the 894 unannotated eligible junctions alone, it reaches 258 of 374 (69.0%) at the 50-target cap, the panel size at which the sweep stops rather than where coverage stops increasing.

Because a panel selected and scored on the same cohort will appear to perform better than it does on new patients, the eligible-set curve was re-evaluated by repeated split-half validation: the cohort was split in half 50 times (seed 20260830), a panel greedily selected on one half, and its coverage scored on the held-out half. In-sample and held-out coverage are close for small panels and diverge as panel size grows: at 1 target, 23.7% in-sample versus 23.2% held-out; at 20 targets, 74.8% versus 63.9% (10.9 points); and at 50 targets, 90.6% versus 67.5%, an optimism gap of 23.2 points (Figure 6b; held-out values in Table 4). The full-cohort curve (*n* = 374) and the split-half curves (selected on *n* = 187, scored on the other *n* = 187) are different calculations and are not directly comparable row for row; the optimism gap is the difference between the split-half in-sample and held-out values (Figure 6b).

A union statistic can conceal the unevenness with which the evidence is distributed across patients. Of the 374 tumours, the median carries four eligible junctions (IQR 2–9, range 0–57), but 29 of 374 (7.8%) carry none, and a further 46 (12.3%) carry exactly one (Figure 6c). These 7.8% set the 92.2% ceiling reported above.

The coverage above is computed over the whole eligible set, most of which has assumed rather than demonstrated absence from normal tissue (Section 3.4). Restricting the pool to the eight junctions supported by the matched-normal criterion changes the curve: coverage tracks the full eligible set for the first three greedy selections and then diverges, saturating at 185 of 374 tumours (49.5%) using seven junctions. Held-out coverage with a budget of 20 targets is 48.5% for this subset (Supplementary Table S16) against 63.9% for the full eligible set (Table 4, Supplementary Figure S4). Re-deriving the subset within each training half, splitting by patient rather than by sample, or both, changes held-out coverage by less than 1 percentage point at any panel size: with a budget of 20 targets, it is 63.4% to 63.9% for the full eligible set and 48.4% to 48.5% for the subset (Supplementary Table S16). Relaxing the normal-side criteria confirms that the eligible-set coverage figure is a function of the amount of normal-tissue signal tolerated, not solely a property of the tumour cohort. Under the same split-half procedure, held-out coverage of a 20-target panel rises from 51.8% at the strictest cell tested (0 of 50 matched normals, 0 of 251 GTEx liver; 726 eligible junctions) to 63.9% at the pre-specified pair of criteria (981 junctions) and to 74.2% at the most permissive cell tested (at most 2 of 50, at most 5 of 251; 1545 junctions) (Supplementary Figure S4).

### 3.6. Sensitivity to Read Thresholds, Recurrence Requirements and Gene Selection

The apparent recurrence ceiling of the panel depends on the tumour-side read threshold used to call a sample positive. This dependency was examined post hoc, as a threshold sweep across values not specified in advance (Supplementary Table S1). At 3 reads, one unannotated junction (Section 3.3.2) reaches the pre-specified 30% recurrence floor, and the unannotated ceiling is 114 of 374; at the pre-specified 5 reads, the ceiling is 88; at 10 reads, it is 53; and at 20 reads, 26. The median recurrence of unannotated junctions remains 1 of 374 at every threshold examined.

The normal-absence axis was examined for the same sensitivity, also post hoc, using the set of 3314 junctions meeting the recurrence criterion alone. Among these, the minimum number of matched normals carrying signal at two reads is 7 of 50; the qualifying count under the matched-normal criterion is therefore zero whether the allowance is set at one, two, or five normals. Of the same 3314 junctions, 2907 (87.7%) are annotated, and every one of the 2907 is present in at least 7 of 50 matched normals.

Two features of the design were tested post hoc as non-biological explanations of the result.

The first is the panel selection rule. Of the 300 genes, 250 were chosen by ranking the HPA secreted and membrane classes on liver nTPM in descending order (Section 2.3), and selecting the genes most abundantly expressed in liver and then finding their junctions present in normal liver would be close to circular. The remaining 50, selected by Open Targets disease association rather than by expression, serve as a control on the rule itself. The pre-specified cascade was recomputed in three groups: the top and bottom halves of the nTPM-ranked 250, and the 50 disease-selected genes (Table 5). The outcome is zero in all three. It is zero in the bottom half of the expression ranking, where the median liver nTPM is 452, against 2016 in the top half, and zero in the disease-selected group, which was never ranked on expression. The three eligible sets also share the property the argument rests on: the median eligible junction appears in one tumour in every group.

Expression-based selection therefore does not account for the zero outcome. It does, however, shape the yield of candidates: the 50 disease-selected genes contribute five of the eight absence-supported junctions against three from the 250 expression-ranked genes, a yield per gene 8.3 times higher (0.100 against 0.012), and the junction covering every tumour lies in A2M, an expression-ranked gene. The gene class from which a panel is drawn is therefore material to the number of demonstrable candidates it can yield, even where it does not change the binary outcome; this bears directly on the scope of the conclusion (Section 3.7 and Section 4.5).

The second feature is the recurrence floor, fixed at 30% in advance; a zero at that floor alone does not separate the absence criteria from the stringency of the recurrence criterion. The cascade was rerun at 5, 10, 15, 20, and 30% (Table 6). No candidate passes at the 30% floor; lower floors recover candidates. The sweep further shows that, at the lowest floor, most of the recovered candidates (15 of 23 at 5%) are those whose absence is not supported by the matched-normal criterion. That no lower floor can add a candidate supported by that criterion follows analytically: the criterion itself requires a tumour prevalence of at least 44 of 374 (11.63%; Section 3.4). The two criteria are coupled through the same variable, tumour prevalence, rather than being independent axes, and no choice of recurrence floor yields more than eight candidates whose absence from matched normal tissue is demonstrated rather than assumed; under the GTEx and joint criteria of Section 3.4, the corresponding numbers are 22 and 25.

The normal-side read floor was examined in the same way (post hoc; Section 2.10, Table 7). With the matched-normal floor, the GTEx floor, or both raised from two to five reads, the eligible set grows from 981 to 1295, 1296, and 2618 junctions, while its character is unchanged: the median eligible junction is carried by 1 of 374 tumours in every cell, and the maximum prevalence remains 88 of 374. The number of eligible junctions reaching 44 of 374 tumours rises from 8 to 9 with the matched-normal floor at five reads and to 15 with both floors at five reads. No cell yields a junction meeting the recurrence floor together with both absence criteria: with the matched-normal floor at five reads, one recurrent junction (chr17:28,368,674–28,369,288; 122 of 374 tumours) has no matched-normal carrier at five reads but is carried by 156 of 251 GTEx liver samples at two reads and by 40 of 251 at five reads. The primary outcome is therefore insensitive to the normal-side read floor. For the 25 junctions reaching the joint informative prevalence, per-sample counts were retrieved individually (Supplementary Table S13): all 25 retain both absence criteria at floors of two, three, and five reads, and 15 retain them at a floor of one read, including 6 of the 8 supported by the matched-normal criterion. For the six with introns longer than 100 kb, three of them among the eight, floors below the recording minimum cannot be evaluated.

Per-sample counts allowed the same analysis at floors of one and three reads (Table 7). Lowering either floor to one read shrinks the eligible set, because junctions with a single normal read are then excluded: with both floors at one read, it comprises 649 junctions, 492 (75.8%) of them carried by one tumour. For 412 of those 649, which have introns longer than 50 kb, a one-read floor cannot be evaluated, since counts below two reads are not recorded for them. With both floors at three reads, the eligible set comprises 1551 junctions. Across all 16 combinations of floors of one, two, three, and five reads, the median eligible junction is carried by 1 of 374 tumours, the maximum prevalence is 88 of 374, and no combination yields a junction meeting the recurrence floor together with both absence criteria.

For junctions with long introns, the effective normal-side floor is higher than two reads. Per-sample counts were retrieved for 13 junctions with introns from 343 bp to 272 kb (Supplementary Table S12a). Those with introns of up to 24 kb were recorded from a single read, whereas no sample carried fewer than two reads on the two junctions with introns of 64 and 69 kb, fewer than three reads on the three of 130 to 199 kb, or fewer than four reads on the four longer than 200 kb. The minimum held for annotated junctions (four, of 64 to 148 kb) as well as for unannotated ones. The recount3 matrices from which the compilations are built confirm it for every junction: among the 45,755 TCGA-LIHC and 40,317 GTEx liver junctions with introns longer than 50 kb, no per-sample count below two, three, or four reads, according to length class, is recorded, whereas junctions with shorter introns carry 19.1 million and 14.8 million single-read entries. This pattern matches the default junction filter documented for the STAR aligner [58], which the recount3 workflow uses for alignment and junction calling [28]. It affects 320 of the 981 eligible junctions (151 with introns of 100 to 200 kb and 169 above 200 kb) and three of the eight absence-supported junctions (the two UGT1A junctions and the UGT2B readthrough), for which absence at two reads is in practice absence at three reads. The tumour floor of five reads is unaffected, and the effective normal-side floor remains below it; the direction of the asymmetry described in Section 3.4 is therefore unchanged.

### 3.7. The Pre-Specified Cascade Applied Genome-Wide (Post Hoc)

The cascade of Section 3.1 was applied, with unchanged thresholds, to every junction in the recount3 matrices of the two projects (Section 2.10). Of 3,011,999 junctions on chromosomes 1 to 22, X, Y, and M, 108,205 met the recurrence criterion, 55 also met the GTEx criterion, and 18 also met the matched-normal criterion (Table 8, Supplementary Section S5.8, and Supplementary Table S17). The zero of Section 3.1 is therefore specific to the panel. The 18 junctions lie at nine loci, none of them in the panel; 15 are annotated, all carry a GT..AG or CT..AC dinucleotide pair, 7 of them within soft-masked sequence (Supplementary Section S5.8), and each is carried by 113 to 171 of 374 tumours, above the informative prevalence of each reference (Section 3.4). Together, they reach 236 of 374 tumours (63.1%, in-sample) at five or more reads. Four have introns of 106 to 128 kb, for which the matched-normal criterion is in effect applied at three reads (Section 3.6), and none of the four has a recorded count in any matched normal sample.

At 17 of the 18 junctions, the remaining junctions of the locus carry less than 3% of the tumour read level per sample in matched normal tissue and less than 5% in GTEx liver (Table 8). The locus as a whole is therefore silent in normal liver, and the junction is absent from normal liver because its gene is not expressed there, not through tumour-specific splicing of a liver-expressed gene. These loci are the cancer-testis genes SSX1 and SSX4 (8 junctions); the testis-expressed gene COX7B2 (2); the long non-coding RNAs LINC01980 (2), LINC01419, LVCAT5, KCNMB2-AS1, and ST8SIA6-AS1; and a region of chromosome 2 that contains no GENCODE v26 gene. The exception is a junction in the pseudogene FRG1GP, whose locus carries 36.3% of the tumour read level in matched normal tissue and 5.5% in GTEx liver. Every junction is carried in GTEx testis, 17 of them by at least 68 of 410 samples, and in the K-562 leukaemia cell line (the GTEx samples labelled bone marrow), and each is carried by tumours of other TCGA projects (median 650 tumours outside TCGA-LIHC). Expression of SSX genes in HCC, with little or none in adjacent tissue, has been reported by RT-PCR [59], and SSX1 mRNA has been detected in peripheral blood mononuclear cells of patients with HCC [60]. In GTEx whole blood, 13 of the 18 junctions have at least one carrier at two or more reads, and none more than 7 of 852 samples.

### 3.8. Plasma Cell-Free RNA Feasibility Check

A separate feasibility check asked whether junction-spanning reads from eight liver-abundant control genes can be recovered from public plasma cell-free RNA; it tested no target from the discovery arm, because that arm produced none. The pre-specified criterion, detection of at least six of the eight control genes in a single designated library, was not met: that library detected three of eight. Three further cancer libraries processed post hoc detected eight, eight, and five of eight, and one healthy-donor library detected one of eight. The designated library was selected by file size and has the lowest depth, alignment rate, exonic fraction, and spliced-alignment fraction of the four, and the set is too small for inference; the check therefore supports no conclusion about recoverability in either direction. Methods and full results are given in Supplementary Section S3, Supplementary Figure S5 and Supplementary Tables S2–S6.

## 4. Discussion

### 4.1. Principal Findings and Their Scope

A fixed-panel splice-junction assay for plasma needs targets that are recurrent across patients and absent from normal tissue. Testing those two requirements separately rather than as one combined filter shows that they are not of equal difficulty in this cohort. Recurrence is readily satisfied: one canonical junction covers every tumour in the cohort once the normal-tissue requirement is removed, because the junctions with the highest tumour prevalence are themselves expressed in normal liver. Absence from normal tissue is the criterion that removes the candidates, and none of the junctions meeting the pre-specified recurrence floor also met both absence criteria.

The negative result has two components that should be kept distinct. Junctions that are prevalent across patients and free of matched-normal carriers do exist, but the eight for which absence is supported at the stated rule lie at three loci and reflect expression differences or a junction also present in other normal tissue, not tumour-specific splicing (below). For these, the limitation is not the size of the reference, and none reaches the 30% floor at the pre-specified five-read threshold. The remaining eligible junctions are rare. They fail the single-target recurrence criterion by definition, irrespective of their status in normal tissue, and the size of the normal reference is not the reason for that failure; it becomes material for the union panel, whose coverage rests on them. For the large majority of eligible junctions, the normal reference is too small to resolve the question in either direction: they are carried by 1 tumour in 374, and at that prevalence, a zero observation in 50 matched normals is expected regardless of whether the junction also occurs in normal liver (Section 3.4). The two criteria are also coupled rather than independent, through tumour prevalence and through expression of the parent gene in normal liver (Section 3.6). Three quantities that are readily conflated should be distinguished. Observing no carrier among 50 normals is a measurement; rejecting a specified normal-to-tumour prevalence ratio is a model-conditional inference; and clinical specificity is neither. The eight junctions identified in Section 3.4 satisfy the second under the null stated there, and are not evidence of absence across a normal population.

The identity of the recurrent junctions is consistent with the same interpretation. The junctions reaching substantial recurrence lie at three loci, none of which shows tumour-specific splicing: the consecutive-exon ladder of full-length TERT, which reports the quantity of transcript present rather than a difference in which exons are joined; alternative-first-exon junctions at UGT1A, where the first exons involved are extrahepatic; and one long-range readthrough into a UGT2B pseudogene, for which no protein product is expected. Readthrough transcription producing chimeric RNAs without genomic rearrangement is established in other tumour types [61,62,63], and cryptic donor-site use has been reported within the UGT1A cluster itself [64]; the UGT2B junction is also carried by normal breast tissue, lies within paired segmental duplications in a region of documented copy-number polymorphism [54,55,56], and remains of unresolved origin (Section 3.3.2). Telomerase is inactive in terminally differentiated cells [65], and TERT promoter mutation [65,66], which occurs in HCC [65], and hepatitis B virus (HBV) integration at the TERT locus [67] are established routes to its reactivation. In this cohort, TERT locus reads form a continuous distribution (Section 3.3.1), and a junction-counting assay at TERT imposes a threshold on a graded expression variable rather than making a binary splicing call. Not being a novel splice event does not by itself disqualify a locus as an assay target; it changes the type of readout the target would require.

The zero is specific to the panel. Applied genome-wide (post hoc), the same criteria are met by 18 junctions (Section 3.7). These junctions extend the first component from the panel to the genome: all but one lie at loci silent in normal liver, at cancer-testis and testis-expressed genes and long non-coding RNAs, and reach tumours through expression of the gene rather than through tumour-specific splicing, as the TERT junctions of the panel do (Section 3.3.1). In this cohort, recurrence together with absence from both liver references is therefore attained where the gene itself, not the choice of splice site, is silent in normal liver.

A further finding bounds the result rather than extending it. The two normal-tissue cohorts are not interchangeable as a specificity reference: tissue adjacent to a tumour is a distinct transcriptional state rather than neutral background [68], and in HCC, it is in addition chronically diseased [22]; the two references also disagree on individual junctions, in both directions, panel-wide and genome-wide, most sharply at the CFH to CFHR4 junction (Section 3.2).

### 4.2. Relation to HCC Splicing and Liquid-Biopsy Studies

The splicing landscape of this tumour type has been characterised well beyond the panel used in this study, and tested in peripheral fluid: 3416 events were reported as commonly mis-spliced in a meta-analysis of 787 samples from 16 studies, and 10 of them measured in circulating epithelial cells separated HCC from non-HCC with 66% sensitivity and 81% specificity [16]. The difference between that result and the present one lies in the selection criterion. Events there were selected by differential inclusion, a criterion a junction can satisfy while remaining present in normal liver at a lower inclusion level. The criterion applied in the present study is absence, the property required by a binary detected-or-not readout at a fixed threshold. The two designs answer different questions, and the present evidence indicates that the stricter of the two criteria is the criterion this panel fails.

Circulating mRNA variants, including splicing aberrations, have also been pursued directly in plasma. Targeted RNA sequencing of 288 candidate variants detected 75 of them in plasma from 50 patients with HCC and 35 patients with cirrhosis [5], and assays for circulating variants have since been developed and tested in tumour tissue and plasma [6]. Those studies select candidates from tumour sequencing and test them in plasma; the present study addresses an earlier step, namely whether absence from normal tissue can be demonstrated for the members of a fixed panel. Junction-level queries of TCGA and GTEx through Snaptron have likewise been used to evaluate candidate tumour-enriched junctions against matched normal tissue [69].

The same distinction separates this work from the pan-cancer neojunction catalogue, which called approximately 930 neojunctions per tumour against a GTEx-only reference, with TCGA normal samples used only as a pooled 1% filter at a later ranking step [17]. Making absence from matched normal tissue a primary criterion, and quantifying the evidence such a test can carry, is the contribution of this study. The question of which normal reference a discovery pipeline uses is not new [23]; the findings added here are which of the two requirements is binding, and that the junctions whose absence is supported against matched normal tissue are those already accounted for by other mechanisms. Recurrent tumour-wide splice neoantigens have been reported at specific loci such as GNAS and RPL22 [18]; both loci were queried post hoc, and neither yielded a junction meeting the criteria used in this study (Section 3.3). Neoantigen discovery pipelines such as SNAF pursue peptide presentation rather than nucleic-acid target recurrence and are not directly comparable [36].

The three-study ccRCC lineage introduced earlier [19,20,21] shows that this target class can be carried from discovery to a plasma-compatible assay, at frequencies above any reached in this study. In that lineage, candidates were filtered against GTEx normal tissues [20], adjacent normal kidney having been examined only descriptively in the first study [19]. Two explanations for the contrast are available and are not mutually exclusive: a difference in the normal reference used at discovery, and a difference in tissue context, since HCC arises against chronic organ-wide parenchymal disease in a manner that adjacent kidney tissue need not. The present data do not separate the two. Independently, a method built to recognise known neoepitopes, fusions, transposable elements, and circular RNAs in cell-free RNA separates HCC patients from healthy controls using neoepitope, transposable-element, and circular-RNA features [70], which establishes that HCC plasma cfRNA carries transcript-level cancer signal without testing either of the properties on which the discovery arm depended.

### 4.3. Implications for Future Assay Design

Four routes remain available; they are set out in the order in which they would have to be resolved.

The first is recovery. Untargeted shotgun sequencing of plasma cfRNA is a poor match for a specific low-abundance junction, and a tumour-specific junction would be expected to lie well below the abundant liver transcripts that such sequencing recovers. Targeted amplification or capture prior to detection is therefore a precondition for testing any candidate, and ultra-sensitive methods for low-abundance extracellular RNA illustrate the scale of enrichment involved [71]. This route addresses recovery, not specificity: an enriched assay for a target that is present in normal liver is still positive in normal liver.

The second is to change the readout rather than the target. A quantitative measurement calibrated against a reference distribution can use a shift in abundance, which is the property offered by the recurrent loci in this panel. TERT is the clearest case: it is absent from normal liver at the threshold used, reaches a quarter of tumours, and accounts for five of the eight junctions whose absence is supported at the criterion of Section 3.4; it is, however, detected in whole blood (Section 3.3.1), and a plasma assay would have to be calibrated against that background. Droplet digital PCR has been used to quantify another splice variant, AR-V7, in blood as absolute copy numbers [13]; for a quantitative readout of this kind, the operative quantities are a limit of blank established in an appropriate reference population and a cut-off fixed to a target specificity, not the absence of any signal. This route replaces a threshold with a distribution, and it requires that the baseline be measured in the population the assay is intended for rather than in histologically normal liver.

The third is to make the target set tumour-informed. Identifying a patient-specific junction from resected tissue and then designing a matched plasma assay circumvents the recurrence requirement, in the manner of circulating-tumour-DNA assays for minimal residual disease that first sequence the tumour; a plasma-only alternative to that design has also been evaluated [72]. It does not remove the need for normal-tissue absence or analytical specificity, and in HCC it restricts the application sharply, because HCC is routinely diagnosed radiologically without biopsy and only a minority of patients undergo resection [1,2]. Standardisation is demanding: in a multinational external quality assessment of MET exon 14 skipping, 49 of 50 institutes (98%) passed the tissue scheme, against 3 of 8 (37.5%) and 10 of 16 (63%) in the two liquid-biopsy rounds [73].

The fourth is to change the target class or to stop treating the panel as a standalone test. A junction-count query of the kind used in this study is blind to intron retention and does not include back-splice junctions or virus-to-human chimeric junctions, neither of which has a counterpart in a normal linear transcript or a normal genome. The coverage ceiling reported in Section 3.5, together with the 7.8% of tumours carrying no eligible target, indicates that a panel of this class is better positioned as one component of a multi-analyte score than as a standalone screening test, and any such positioning has to be argued against the performance of the surveillance standard it would supplement [3].

In this gene class and cohort, the discovery arm excludes a fixed panel of tumour-specific splice events whose absence from normal tissue is demonstrated. It does not exclude every fixed panel: a union of 20 individually uncommon junctions reaches 63.9% of tumours on held-out evaluation within this cohort (Section 3.5), on targets whose absence from normal tissue is assumed rather than demonstrated. Genome-wide (post hoc), 18 junctions meet the pre-specified criteria and together reach 63.1% of tumours in-sample (Section 3.7); a panel drawn from them would read expression of genes silent in normal liver, and its specificity in plasma would depend on the carriers observed in testis, in other tumour types, and in whole blood.

### 4.4. Methodological Implication for Absence-Based Filters

The specific result reported in this study concerns splice junctions in one gene panel. The evidential limit on rare candidates follows from the sampling model rather than from the target class, and it is stated here as a methodological implication that was not tested on other RNA classes. A pipeline that nominates an RNA species as disease-specific because it was not detected in a set of normal reference samples makes a claim whose strength is set by two quantities: the prevalence of the candidate among cases, and the size of the normal reference. The relation is the one that governs any zero numerator: with no event among n observations, the upper 95% confidence limit of the event rate is approximately 3/n [74,75]. Where candidates are common, a modest reference resolves the question; where candidates are rare, a zero observation is the expected outcome irrespective of whether the candidate occurs in normal tissue. Because the arithmetic does not depend on the class of RNA counted, the same reasoning would be expected to apply to miRNA, lncRNA, and circRNA candidate sets that contain low-prevalence species and are filtered against a small normal reference. Where both quantities are reported, the calculation requires no access to the underlying data. In the present screen, the calculation moved the number of candidates with supported absence from 981 to 8 under the matched-normal criterion and to 25 under the joint criterion, and none of the 8 showed the tumour-specific splicing the screen was designed to detect.

### 4.5. Limitations

The pre-specified result is scoped to one panel, one gene class, one cohort, and one tissue context, and the design was unfavourable to a positive result in three respects. The panel was drawn mainly from secreted and membrane protein classes chosen for plasma recoverability rather than for splicing novelty, and 250 of its 300 slots were filled by ranking those classes on liver abundance. The readout was a binary call at a fixed raw-read threshold. Absence was required against adjacent tissue of a chronically diseased organ. The gene selection does not produce the binary result, but it shapes the yield of demonstrable candidates and excludes loci reported elsewhere as recurrent neojunction sources, such as RPL22 [18] (Section 3.3 and Section 3.6, Table 5). The conclusion is therefore that demonstrable absence was not obtained from these liver references at these thresholds for this gene class.

The specificity axis tested in this study is absence from liver tissue. A plasma assay is read against the cell-free transcriptome, whose dominant contributors are haematopoietic rather than hepatic [76]; a junction absent from normal liver is consequently not established as absent from the background a plasma test would encounter. Of the eight absence-supported junctions, the five at TERT were detected in GTEx whole blood (post hoc; 11 to 45 of 852 samples, Supplementary Table S9); no leukocyte-specific or plasma reference was examined, and the remaining 973 eligible junctions were not queried outside liver. The matched normals also derive from surgical resection specimens, from patients with relatively preserved liver function [1,2], and need not represent the parenchymal background of the cirrhotic population in which such a test would be used.

The thresholds and the null model both shape the numbers. The 30% recurrence floor was fixed in advance as a single-target endpoint (Section 2.4); lower floors recover candidates, but no more than 25 whose absence is supported under any criterion (Section 3.6, Table 6). The normal-side read floor was examined at one, two, three, and five reads, and for long introns the compilations do not record low counts, which makes absence easier to satisfy for 320 of the 981 eligible junctions (Section 3.6, Table 7). The count of eight absence-supported junctions is conditional on the null of Section 3.4, is uncorrected for multiple comparisons across the eligible set, and is a lower bound, because normal-side presence was scored at a lower read threshold than tumour prevalence. Carrier calls use raw read counts without depth normalisation (depth differences in Section 3.4), and tumour purity and possible tumour or field-effect content in the adjacent normal samples were not assessed.

The coverage figures rest on split-half evaluation within the same 374 tumours, with the eligible set defined on that cohort. The genome-wide analysis is post hoc: its union coverage of 63.1% is in-sample, its 18 junctions were not evaluated in an independent HCC cohort or in plasma, and carriers of them were observed in testis, in other tumour types and in whole blood (Section 3.7). The unannotated recurrence ceiling rests on a single junction of unresolved origin: a copy-number origin is not supported, and realignment to alternate haplotypes was not performed (Section 3.3.2). The positive control of Section 3.3 shows that the query recovers two established oncogenic junctions in other tumour types; it does not establish the sensitivity of the screen to an unknown recurrent junction in liver.

The plasma feasibility check is small and control-based (Section 3.8).

## 5. Conclusions

Under pre-specified criteria, no junction in a 300-gene panel of secreted, membrane-protein and HCC-associated genes was both recurrent across patients and absent from normal liver in hepatocellular carcinoma. The binding requirement was demonstrable absence from normal tissue, not recurrence.

Absence becomes demonstrable only above a tumour prevalence set by the size of the normal reference, 11.63% for 50 matched normals. Most eligible junctions lie far below it, and the eight above it, at TERT, UGT1A, and UGT2B, are accounted for by mechanisms other than tumour-specific splicing.

Applied genome-wide (post hoc), the same criteria are met by 18 junctions, 17 of them at loci silent in normal liver; where recurrence and absence coincide, the gene, not the splice choice, is silent in normal liver.

Three implications extend beyond this panel. First, as a methodological implication of the sampling model, non-detection in a normal reference is evidence of absence only above a candidate prevalence fixed by the size of that reference, *p_min_* = (1 − alpha^1/n^)/f for a zero-observation criterion (Figure 5b): 11.63% for 50 normals, and 2240 normals would be needed for a junction carried by 1 tumour in 374. Catalogues of tumour-specific junctions, transcripts, or neoantigens filtered by absence from normal tissue should report this prevalence for the reference employed and should describe candidates below it as having assumed, not demonstrated, absence. Second, the normal reference is a design decision rather than a technical detail: matched adjacent tissue and GTEx liver disagree on individual junctions in both directions, panel-wide and genome-wide, most sharply at a CFH to CFHR4 junction carried by 47 of 50 matched normals and by none of 251 GTEx liver samples. Third, in this cohort, recurrent junctions absent from normal liver arise from expression of genes silent in normal liver, such as cancer-testis genes and long non-coding RNAs, rather than from tumour-specific splicing of liver-expressed genes. A fixed panel built on such junctions would draw its specificity from gene silence and would inherit the expression of those genes in testis, in other tumour types, and in blood.

The design foreclosed is narrow: a fixed binary panel of tumour-specific splice events with demonstrated absence from normal liver, in this gene class and cohort. Enrichment, a quantitative readout against a reference distribution, a tumour-informed per-patient design, and a change in target class or multi-analyte use remain open (Section 4.3).

## Figures and Tables

**Table 1:** Cohort-level comparison of TCGA-LIHC matched normal and GTEx liver (post hoc).

Measure	TCGA-LIHC Matched Normal	GTEx Liver	Ratio (TCGA/GTEx)	*p*
ALB coverage, reads/10^6^ (median)	314,890.1 (*n* = 50)	185,611.2 (*n* = 251)	1.70	1.57 × 10^−9^
CFH-cluster coverage, reads/10^6^ (median)	8485.2 (*n* = 50)	7949.4 (*n* = 251)	1.07	0.107

**Table 2:** Paired tumour and matched-normal counts (post hoc, descriptive). The junctions were selected for zero carriers among the 50 matched normals, and the zero in the fourth column follows from that selection. The final column, 0.5 raised to the number of paired carriers, is the probability of that zero under an illustrative null in which the junction is present in half of normal samples; it indicates relative evidential weight and is not a hypothesis test.

Junction	Gene(s)	Paired Tumour Carriers	Carriers with Signal in Own Normal	Null Probability
chr4:69005136–69204429: −	UGT2B11 to UGT2B27P	15	0	3.1 × 10^−5^
chr5:1258660–1260473: −	TERT	8	0	3.9 × 10^−3^
chr2:233637378–233767033: +	UGT1A10 to UGT1A1	9	0	2.0 × 10^−3^
chr5:1260601–1264403: −	TERT	6	0	1.6 × 10^−2^
chr2:233618563–233767033: +	UGT1A8 to UGT1A1	8	0	3.9 × 10^−3^
chr5:1266536–1268519: −	TERT	5	0	3.1 × 10^−2^
chr5:1255412–1258597: −	TERT	5	0	3.1 × 10^−2^
chr5:1254506–1255286: −	TERT	5	0	3.1 × 10^−2^
chr5:1268634–1271118: −	TERT	4	0	6.3 × 10^−2^
chr5:1278797–1279290: −	TERT	4	0	6.3 × 10^−2^
chr5:1264593–1266463: −	TERT	3	0	1.3 × 10^−1^
chr5:1280339–1282428: −	TERT	3	0	1.3 × 10^−1^

**Table 3:** Power of the absence filters by normal reference, for an assumed normal-to-tumour prevalence ratio f (post hoc). Expected numbers are upper bounds, and counts of supported junctions are lower bounds (see text).

Reference Criterion and *f*	Expected to Pass by Chance, Upper Bound	Junctions with *p* < 0.05, Lower Bound
Matched normal (0 of 50), *f* = 0.10	97.0% (951.5 of 981)	0
Matched normal (0 of 50), *f* = 0.25	93.4% (916.0 of 981)	1
Matched normal (0 of 50), *f* = 0.50	88.5% (867.7 of 981)	8
GTEx liver (at most 2 of 251), *f* = 0.10	98.7% (968.7 of 981)	0
GTEx liver (at most 2 of 251), *f* = 0.25	96.9% (950.6 of 981)	11
GTEx liver (at most 2 of 251), *f* = 0.50	94.1% (922.9 of 981)	22
Both references jointly, *f* = 0.10	96.3% (944.7 of 981)	1
Both references jointly, *f* = 0.25	92.0% (903.0 of 981)	17
Both references jointly, *f* = 0.50	85.8% (842.0 of 981)	25

**Table 4:** Greedy maximum-coverage panels: full cohort, split-half held-out evaluation, and the absence-supported subset (post hoc).

Panel Size	1	2	3	5	10	20	50
All eligible, full cohort	23.5%	37.4%	46.3%	55.9%	64.2%	72.2%	82.1%
Held-out (split-half, mean ± SD)	23.2 ± 2.8%	35.7 ± 3.1%	44.3 ± 3.5%	53.5 ± 3.4%	59.9 ± 3.3%	63.9 ± 4.0%	67.5 ± 3.4%
Absence-supported only (*n* = 8 junctions)	23.5%	37.4%	46.3%	48.7%	49.5%	49.5%	saturated

**Table 5:** Pre-specified cascade by gene selection route (post hoc). Gene counts are panel members; 4 of the 300 did not resolve against GENCODE v26 and were not queried (Section 2.3). Counts in the recurrence and later columns are of canonical-motif junctions. Each of the 776,855 tcgav2 junctions is assigned to the panel gene that contains it or, failing that, overlaps it (775,808 assigned); Section 3.1 assigns by the gene containing the junction start (1,002,937), a count that also includes junctions returned only by the gtexv2 compilation and junctions of AR. The recurrence column recovers 3155 of the 3163 canonical junctions meeting the 30% floor (99.7%); the assignment loss therefore falls almost entirely on junctions that do not approach the recurrence criterion.

Gene Group	Genes	Junctions Assigned	Meets 30% Recurrence	+GTEx-Absent	+Matched-Normal Absent	Eligible Set	Median Prevalence	In One Tumour	Absence-Supported	Ceiling
nTPM-ranked, top half	125	342,801	1418	1	0	400	1 of 374	325 (81.3%)	1	88
nTPM-ranked, bottom half	125	320,223	1101	0	0	471	1 of 374	343 (72.8%)	2	61
Disease-selected (not ranked on expression)	50	112,784	636	0	0	110	1 of 374	72 (65.5%)	5	71

**Table 6:** Recurrence-floor sweep (post hoc). Counts are restricted to canonical-motif junctions; the 30% row therefore reads 3163 where Section 3.1 reads 3314; Section 3.1 reports the pre-specified cascade before the motif restriction is applied. The final column applies the matched-normal absence-power criterion of Section 3.4, which requires a tumour prevalence of at least 44 of 374.

Recurrence Floor	Meets Recurrence	+GTEx-Absent	+Matched-Normal Absent	Of Those, Absence-Supported
5% (at least 18 of 374)	4502	64	23	8
10% (at least 37 of 374)	4029	30	12	8
15% (at least 56 of 374)	3731	13	6	6
20% (at least 74 of 374)	3520	6	1	1
30% (at least 112 of 374, pre-specified)	3163	1	0	0

**Table 7:** Sensitivity to the normal-side read floor (post hoc). The tumour floor is fixed at five reads; the matched-normal criterion is 0 carriers of 50, and the GTEx criterion at most 2 carriers of 251, each at the stated floor. The fourth column gives the eligible junctions reaching the informative prevalence of the matched-normal, GTEx, and joint criteria. The first row is the pre-specified pair of floors. The last column gives the eligible junctions whose introns are long enough that counts below the stated floor are not recorded (matched normal/GTEx); for these, the floor acts as the recording minimum of two, three, or four reads.

Read Floor (Matched Normal, GTEx)	Eligible Junctions	Carried by One Tumour	Reaching 44/19/15 of 374	Meeting Recurrence and Both Absence Criteria	Floor Not Evaluable (Matched Normal/GTEx)
2 reads, 2 reads (pre-specified)	981	740 (75.4%)	8/22/25	0	320/320
1 read, 1 read	649	492 (75.8%)	6/13/15	0	412/412
1 read, 2 reads	749	567 (75.7%)	8/17/19	0	412/320
1 read, 3 reads	776	586 (75.5%)	8/17/19	0	419/169
1 read, 5 reads	805	604 (75.0%)	8/17/19	0	429/0
2 reads, 1 read	712	542 (76.1%)	6/15/17	0	320/412
2 reads, 3 reads	1164	855 (73.5%)	8/23/27	0	320/169
2 reads, 5 reads	1296	940 (72.5%)	8/23/27	0	328/0
3 reads, 1 read	774	575 (74.3%)	6/16/19	0	169/446
3 reads, 2 reads	1166	851 (73.0%)	8/24/28	0	169/320
3 reads, 3 reads	1551	1084 (69.9%)	8/27/33	0	169/169
3 reads, 5 reads	1924	1258 (65.4%)	8/34/42	0	171/0
5 reads, 1 read	841	605 (71.9%)	7/19/24	0	0/492
5 reads, 2 reads	1295	912 (70.4%)	9/28/35	0	0/351
5 reads, 3 reads	1843	1204 (65.3%)	9/38/50	0	0/180
5 reads, 5 reads	2618	1508 (57.6%)	15/80/104	0	0/0

**Table 8:** Junctions meeting the three pre-specified criteria genome-wide, by locus (post hoc; recount3 project-level junction matrices). Carriers are counted at two or more reads in GTEx testis and whole blood. Locus expression is the mean number of reads per sample over the other same-strand junctions of the locus, in matched normal tissue and in GTEx liver, as a percentage of the tumour value. Per-junction values are given in Supplementary Table S17.

Locus (GENCODE v26)	Junctions (Annotated)	Tumours, ≥5 Reads (of 374)	GTEx Testis Carriers (of 410)	GTEx Whole-Blood Carriers (of 852)	Locus Reads, Matched Normal/GTEx Liver (% of Tumour)
SSX1 and SSX4, chrX	8 (5)	113–125	363–398	0–4	0.1–0.2/0.0–0.1
COX7B2, chr4	2 (2)	133–138	396–398	1	0.0/0.8
LINC01980, chr3	2 (2)	115–133	68–193	0–1	0.0/1.3
LINC01419, chr8	1 (1)	131	120	0	0.0/0.0
LVCAT5, chr7	1 (1)	116	142	1	0.0/0.2
KCNMB2-AS1, chr3	1 (1)	124	7	0	2.0/4.5
ST8SIA6-AS1, chr10	1 (1)	171	390	7	0.9/0.7
No GENCODE v26 gene, chr2	1 (1)	154	288	0	2.6/1.0
FRG1GP, chr22	1 (1)	116	106	2	36.3/5.5

## Data Availability

All primary data analysed in this study are public. Splice junction counts were obtained from the Snaptron/recount3 tcgav2 and gtexv2 compilations (GRCh38, GENCODE v26). Plasma cell-free RNA libraries are available from the Sequence Read Archive under study SRP239389 (Gene Expression Omnibus series GSE142987) [38]. The analysis code, the frozen threshold record (thresholds.json), the consolidated junction tables retrieved from Snaptron, all intermediate tables, and all figure-generating scripts are deposited at Zenodo (https://doi.org/10.5281/zenodo.22814921). Supplementary Section S4 maps each table and figure in this manuscript to the specific data file it is derived from.

## Supplementary Materials

The following supporting information can be downloaded at https://www.mdpi.com/article/10.3390/biomedicines14102396/s1. Supplementary Section S1: Normal-reference comparison at the CFH locus; Supplementary Section S2: Motif case-sensitivity check and count definitions; Supplementary Section S3: Plasma cell-free RNA feasibility check; Supplementary Section S4: Data provenance by table and section; Supplementary Section S5: Additional reference and control analyses; Figure S1: Of the normal-absent junctions, 14.9% carry non-canonical splice motifs, against 2.8% of annotated junctions. Splice-motif composition of the normal-absent set against the annotated GENCODE v26 junctions of the same genes, from motif_composition.tsv. Motifs are the donor and acceptor dinucleotides reported by Snaptron read on the plus strand; canonical means GT-AG or its minus-strand representation CT-AC. Percentages are of junctions within each set. The annotated set serves as an internal reference for genuine splicing in these data. Post-hoc analysis. The normal-absent set is 83.5% canonical and 1.6% minor-spliceosome, against 97.0% canonical for the annotated reference (n = 7918). Motifs (matched without regard to letter case) and annotation are taken from either compilation; the excess of non-canonical motifs is consistent with a non-splicing component; Figure S2: TCGA adjacent normal and GTEx liver are not interchangeable as a normal reference. a, Depth-normalised ALB locus junction coverage in TCGA-LIHC matched normal against GTEx liver, from cfh_tissue_state.tsv, one point per sample with group medians as horizontal bars. b, The same comparison for the CFH gene cluster (CFH, CFHR1, CFHR2, CFHR3), which did not differ significantly between the cohorts. Post-hoc analysis. ALB is 1.70-fold higher in TCGA-LIHC normal than in GTEx liver (Mann–Whitney *p* = 1.57 × 10^−9^) whereas the CFH cluster differs by 1.07-fold (*p* = 0.107). A junction at the CFH locus that passed the GTEx criterion was carried by none of 251 GTEx liver samples and by 47 of 50 matched normals; the mechanism is undetermined (Supplementary Section S1); Figure S3: Paired tumour carriers of the 12 most recurrent canonical, normal-absent junctions among the 50 patients with a matched normal. Paired matched-normal comparison from paired_normal_test.tsv. For each of the 12 canonical, normal-absent junctions with the highest recurrence, bars give the number of patients whose own tumour carries the junction among the 50 with a matched normal; the annotation gives the number of those same patients carrying it in their own normal and the value 0.5^m^, where m is the number of paired tumour carriers, which is the probability of zero carriers among m normals under an illustrative null in which the junction is present in half of normals. Samples are paired on the TCGA patient barcode. Gene pairs are named in transcriptional order. Post-hoc analysis. The 12 junctions were selected for having no carrier at 2 or more reads among these same 50 matched normals; the count of 0 own-normal carriers is therefore fixed by the selection and is not an independent observation, and the 0.5^m^ values are illustrative arithmetic, not tests. The junctions have 3 to 15 paired tumour carriers; four have at least 8 (0.5^m^ at most 0.0039) and four have at most 4 (0.5^m^ at least 0.0625). Carrier counts are per patient; the junction carried by 88 tumour samples is carried by 87 patients; Figure S4: The coverage a multi-target panel reaches depends on which junctions are allowed into it, and on the amount of normal-tissue signal the eligibility criterion tolerates. a, Greedy coverage on the full cohort before and after removing junctions whose absence from normal tissue is not statistically supported, from false_absence_supported_coverage.tsv, under the rule of main text Section 3.4. The two curves are identical for the first three targets and diverge thereafter; the supported subset saturates while the full eligible set continues to rise. b, Held-out coverage of a 20-target panel across the normal-side criterion grid, from normal_side_sweep.tsv, using the split-half procedure of main text Figure 6b. Coverage rises monotonically as the criterion is relaxed. Post-hoc analysis; thresholds.json is unchanged; Figure S5: Control junctions are recovered from all four cancer libraries, two of which detect all 8 control genes while the designated library does not, and detection tracks the spliced fraction of the library more closely than its read count. a, Read attrition from sequenced reads to sensitivity-control junctions across the four plasma cell-free RNA libraries, from detection_funnel.tsv. Bars are four-library medians and points are individual libraries. Every stage is expressed in reads rather than read pairs; the funnel is therefore monotonic, and stages from primary alignments onward count primary alignments only. b, Method 1 reads at the best junction of each of the eight pre-specified sensitivity-control genes, from per_library_sensitivity.tsv. The axis is logarithmic; genes with no reads are therefore drawn below the axis floor and labelled 0; the dashed line is one junction-spanning read. Colours are the same libraries as in a. c, Sensitivity-control genes detected against the spliced fraction of primary alignments, one point per library, with the pre-specified criterion of 6 of 8 drawn. With n = 4, the rank correlations are descriptive; no trend was fitted and no *p*-value was computed. Post-hoc analysis, except for the primary library, on which the pre-specified criterion is defined; Figure S6: The two normal references agree on common junctions and disagree on a minority. Detection fraction of each junction passing the evidence floor (n = 12,783) in the 50 matched normal samples against the 251 GTEx liver samples, at 2 or more reads, binned in two dimensions with a logarithmic colour scale; the dashed line is equality. Spearman rho = 0.839 over the 11,822 junctions detected in at least one reference. Junctions along the axes are detected in one reference only: 73 are carried by at least 10% of matched normal samples and by no GTEx sample, and 514 show the converse. From reference_concordance_bins.tsv. Post-hoc analysis; Figure S7: The pattern of agreement between the two normal references holds genome-wide. Detection fraction of each junction passing the evidence floor genome-wide (n = 335,637; recount3 project-level junction matrices) in the 50 matched normal samples against the 251 GTEx liver samples, at 2 or more reads, binned in two dimensions with a logarithmic colour scale; the dashed line is equality. Spearman rho = 0.898 over the 305,567 junctions detected in at least one reference. Post-hoc analysis; data from reference_concordance_genome_bins.tsv; Table S1: Threshold sweep of junctions detected in at least 1 tumour (post hoc). Counts are computed on the case-insensitive canonical set (Supplementary Section S2); Table S2: Library QC, four cancer libraries; Table S3: Per-gene Method 1 maxima, four cancer libraries; Table S4: Negative and specificity controls, per library; Table S5: Sensitivity controls, per library; pre-specified criterion 6 of 8; Table S6: Cancer libraries versus the healthy-donor library; Table S7: Junction and gene counts used in this report, and the filter each is computed under; Table S8: Positive-control junctions and their flanking junctions (post hoc). Carriers are samples with at least 1, 2 or 5 reads; the main-screen floors are 5 reads for tumour samples and 2 reads for normal samples; Table S9: Tissue distribution of the eight absence-supported junctions in GTEx (post hoc). The GTEx samples labelled Bone Marrow are the K-562 chronic myeloid leukaemia cell line. GTEx carriers are samples with at least 2 reads among 19,214; liver is of 251 samples and whole blood of 852; Table S10: a. Carriers of the UGT2B11 to UGT2B27P junction by TCGA project and sample type (post hoc). Tumour samples are those with a sample-type code below 10 and normal samples those with a code of 10 to 19 in the TCGA barcode. b. Segmental duplications (UCSC Genome Browser [48], track genomicSuperDups, GRCh38) containing each splice site of the UGT2B11 to UGT2B27P junction; Table S11: Junction detection in matched normal tissue (n = 50) and GTEx liver (n = 251) (post hoc). Spearman rho is the correlation of detection fractions over junctions detected in at least one reference. The last four count columns give junctions detected in at least 10% or 50% of one reference with no carrier in the other; Table S12: a. Minimum per-sample read count recorded for junctions of increasing intron length (exact-coordinate queries; post hoc). b. Junctions by intron-length class and the minimum read count recorded in that class; Table S13: Normal-side carriers of the 25 eligible junctions reaching 15 of 374 tumours, at read floors of 1, 2, 3 and 5 (exact-coordinate queries; post hoc). For introns longer than 100 kb and 200 kb, samples with fewer than 3 and 4 reads are not recorded; Table S14: Copy number of the interval between the splice sites of the UGT2B11 to UGT2B27P junction, by junction-carrier status of the tumour at 3 or more reads (post hoc; exploratory, uncorrected). Log2 ratios are length-weighted means of SNP-array segments; tests are Mann–Whitney U for medians and Fisher exact for proportions. Comparisons use one DNA sample per patient, preferring the sample matching the RNA tumour sample (GDC tumour DNA: 379 samples from 375 patients; Xena tumour DNA: 372 samples from 370 patients; blood-derived and solid-tissue normal DNA: one sample per patient), and are restricted to patients with an RNA tumour sample, from which carrier status is defined; this excludes 6, 6, 2 and 3 patients respectively and gives denominators of 369 (GDC tumour), 364 (Xena tumour), 304 (blood-derived normal) and 80 (solid-tissue normal) patients. The last three rows of each DNA type give the same comparison at UGT2B28 and UGT2B17 (75_ugt2b28_copy_number.py); Table S15: Junction detection in matched normal tissue (n = 50) and GTEx liver (n = 251), genome-wide (post hoc; recount3 project-level junction matrices). All rows are junctions passing the evidence floor; columns as in Supplementary Table S11. The evidence floor takes the motif from the TCGA-LIHC project; the panel rows apply the same definition to the panel junctions for comparison. The last row also admits junctions present only in GTEx liver, with their GTEx motif; Table S16: Held-out tumour coverage, mean (SD) in %, of greedy panels by panel size, over 50 split-half partitions with the seed of main text Section 3.5 (post hoc). a, splits by sample, pool and subset defined on all 374 tumours (the evaluation of main text Table 4); b, splits by patient; c, nested: pool and eight-junction subset re-derived within each training half; d, nested and split by patient. Subset (8): the eight-junction subset of main text Section 3.4; Table S17: Junctions meeting the three pre-specified criteria genome-wide (post hoc; recount3 project-level junction matrices and exact-coordinate queries of tcgav2 and gtexv2). Gene: GENCODE v26 genes on the junction strand, pseudogenes listed only where no other gene overlaps. Motif as returned by Snaptron, lower case marking soft-masked sequence. Matched normal carriers at 1 or more reads and GTEx liver carriers at 2 or more reads. Other TCGA tumours: tumour samples of TCGA projects other than TCGA-LIHC carrying 5 or more reads. Locus reads: mean reads per sample over the other same-strand junctions of the locus, in matched normal tissue and GTEx liver, as a percentage of the tumour value.

## References

[1] Singal A.G., Llovet J.M., Yarchoan M., Mehta N., Heimbach J.K., Dawson L.A., Jou J.H., Kulik L.M., Agopian V.G., Marrero J.A. (2023). AASLD Practice Guidance on prevention, diagnosis, and treatment of hepatocellular carcinoma. Hepatology.

[2] European Association for the Study of the Liver (2025). EASL Clinical Practice Guidelines on the management of hepatocellular carcinoma. J. Hepatol..

[3] Tzartzeva K., Obi J., Rich N.E., Parikh N.D., Marrero J.A., Yopp A., Waljee A.K., Singal A.G. (2018). Surveillance Imaging and Alpha Fetoprotein for Early Detection of Hepatocellular Carcinoma in Patients with Cirrhosis: A Meta-analysis. Gastroenterology.

[4] Shetti D., Mallela V.R., Ye W., Sharif M., Ambrozkiewicz F., Trailin A., Liška V., Hemminki K. (2024). Emerging role of circulating cell-free RNA as a non-invasive biomarker for hepatocellular carcinoma. Crit. Rev. Oncol. Hematol..

[5] Zezulinski D., Hoteit M.A., Kaplan D.E., Simeone A., Zhan T., Doria C., Ahmed F.Y., Roberts L.R., Block T.M., Sayeed A. (2025). Detection of Circulating mRNA Variants in Hepatocellular Carcinoma Patients Using Targeted RNAseq. Liver Cancer.

[6] Zezulinski D., Hoteit M.A., Kaplan D.E., Zhan T., Doria C., Block T.M., Sayeed A. (2026). Validation of next-generation sequencing-derived circulating mRNA variants as potential diagnostic tools in hepatocellular carcinoma and cholangiocarcinoma. Oncol. Rep..

[7] Bradley R.K., Anczuków O. (2023). RNA splicing dysregulation and the hallmarks of cancer. Nat. Rev. Cancer.

[8] Wang E., Aifantis I. (2020). RNA Splicing and Cancer. Trends Cancer.

[9] Sveen A., Kilpinen S., Ruusulehto A., Lothe R.A., Skotheim R.I. (2016). Aberrant RNA splicing in cancer; expression changes and driver mutations of splicing factor genes. Oncogene.

[10] Anczukow O., Allain F.H., Angarola B.L., Black D.L., Brooks A.N., Cheng C., Conesa A., Crosse E.I., Eyras E., Guccione E. (2024). Steering research on mRNA splicing in cancer towards clinical translation. Nat. Rev. Cancer.

[11] Antonarakis E.S., Lu C., Wang H., Luber B., Nakazawa M., Roeser J.C., Chen Y., Mohammad T.A., Chen Y., Fedor H.L. (2014). AR-V7 and resistance to enzalutamide and abiraterone in prostate cancer. N. Engl. J. Med..

[12] Scher H.I., Lu D., Schreiber N.A., Louw J., Graf R.P., Vargas H.A., Johnson A., Jendrisak A., Bambury R., Danila D. (2016). Association of AR-V7 on Circulating Tumor Cells as a Treatment-Specific Biomarker with Outcomes and Survival in Castration-Resistant Prostate Cancer. JAMA Oncol..

[13] Ma Y., Luk A., Young F.P., Lynch D., Chua W., Balakrishnar B., de Souza P., Becker T.M. (2016). Droplet Digital PCR Based Androgen Receptor Variant 7 (AR-V7) Detection from Prostate Cancer Patient Blood Biopsies. Int. J. Mol. Sci..

[14] Frampton G.M., Ali S.M., Rosenzweig M., Chmielecki J., Lu X., Bauer T.M., Akimov M., Bufill J.A., Lee C., Jentz D. (2015). Activation of MET via diverse exon 14 splicing alterations occurs in multiple tumor types and confers clinical sensitivity to MET inhibitors. Cancer Discov..

[15] Paik P.K., Felip E., Veillon R., Sakai H., Cortot A.B., Garassino M.C., Mazieres J., Viteri S., Senellart H., Van Meerbeeck J. (2020). Tepotinib in Non-Small-Cell Lung Cancer with MET Exon 14 Skipping Mutations. N. Engl. J. Med..

[16] Hershberger C.E., Daniels N.J., Patterson W.M., Rotroff D.M. (2026). The alternative splicing landscape of hepatocellular carcinoma and its potential for HCC detection. Hepatol. Commun..

[17] Kahles A., Lehmann K.V., Toussaint N.C., Hüser M., Stark S.G., Sachsenberg T., Stegle O., Kohlbacher O., Sander C., Cancer Genome Atlas Research Network (2018). Comprehensive Analysis of Alternative Splicing Across Tumors from 8,705 Patients. Cancer Cell.

[18] Kwok D.W., Stevers N.O., Etxeberria I., Nejo T., Colton Cove M., Chen L.H., Jung J., Okada K., Lakshmanachetty S., Gallus M. (2025). Tumour-wide RNA splicing aberrations generate actionable public neoantigens. Nature.

[19] Zaman S., Hajiran A., Coba G.A., Robinson T., Madanayake T.W., Segarra D.T., Chobrutskiy B.I., Boyle T.A., Zhou J.M., Kim Y. (2021). Aberrant Epidermal Growth Factor Receptor RNA Splice Products Are Among the Most Frequent Somatic Alterations in Clear Cell Renal Cell Carcinoma and Are Associated with a Poor Response to Immunotherapy. Eur. Urol. Focus.

[20] Chang A., Chakiryan N.H., Du D., Stewart P.A., Zhang Y., Tian Y., Soupir A.C., Bowers K., Fang B., Morganti A. (2022). Proteogenomic, Epigenetic, and Clinical Implications of Recurrent Aberrant Splice Variants in Clear Cell Renal Cell Carcinoma. Eur. Urol..

[21] Avasthi K.K., Yim K.H., Kim Y., Echevarria P.M.R., Darst L.M., Abreu N., Zea K.P., Hayes M.T., Vosoughi A., Manley B.J. (2026). Multiplex RT-PCR-Based Sequencing Assay to Detect Kidney Cancer-Specific Splice Variants in Tumor Tissues and Plasma Cell-Free RNAs. Mol. Diagn. Ther..

[22] Ng C.K.Y., Dazert E., Boldanova T., Coto-Llerena M., Nuciforo S., Ercan C., Suslov A., Meier M.A., Bock T., Schmidt A. (2022). Integrative proteogenomic characterization of hepatocellular carcinoma across etiologies and stages. Nat. Commun..

[23] Palmer T., Kessler M.D., Shao X.M., Balan A., Yarchoan M., Zaidi N., Lopez-Vidal T.Y., Saeed A.M., Gore J., Azad N.S. (2024). SpliceMutr Enables Pan-Cancer Analysis of Splicing-Derived Neoantigen Burden in Tumors. Cancer Res. Commun..

[24] Zucman-Rossi J., Villanueva A., Nault J.C., Llovet J.M. (2015). Genetic Landscape and Biomarkers of Hepatocellular Carcinoma. Gastroenterology.

[25] Cancer Genome Atlas Research Network (2017). Comprehensive and Integrative Genomic Characterization of Hepatocellular Carcinoma. Cell.

[26] Schulze K., Imbeaud S., Letouzé E., Alexandrov L.B., Calderaro J., Rebouissou S., Couchy G., Meiller C., Shinde J., Soysouvanh F. (2015). Exome sequencing of hepatocellular carcinomas identifies new mutational signatures and potential therapeutic targets. Nat. Genet..

[27] Wilks C., Gaddipati P., Nellore A., Langmead B. (2018). Snaptron: Querying splicing patterns across tens of thousands of RNA-seq samples. Bioinformatics.

[28] Wilks C., Zheng S.C., Chen F.Y., Charles R., Solomon B., Ling J.P., Imada E.L., Zhang D., Joseph L., Leek J.T. (2021). recount3: Summaries and queries for large-scale RNA-seq expression and splicing. Genome Biol..

[29] GTEx Consortium (2020). The GTEx Consortium atlas of genetic regulatory effects across human tissues. Science.

[30] Frankish A., Diekhans M., Ferreira A.M., Johnson R., Jungreis I., Loveland J., Mudge J.M., Sisu C., Wright J., Armstrong J. (2019). GENCODE reference annotation for the human and mouse genomes. Nucleic Acids Res..

[31] Uhlén M., Fagerberg L., Hallström B.M., Lindskog C., Oksvold P., Mardinoglu A., Sivertsson Å., Kampf C., Sjöstedt E., Asplund A. (2015). Tissue-based map of the human proteome. Science.

[32] Ochoa D., Hercules A., Carmona M., Suveges D., Gonzalez-Uriarte A., Malangone C., Miranda A., Fumis L., Carvalho-Silva D., Spitzer M. (2021). Open Targets Platform: Supporting systematic drug-target identification and prioritisation. Nucleic Acids Res..

[33] David J.K., Maden S.K., Weeder B.R., Thompson R.F., Nellore A. (2020). Putatively cancer-specific exon-exon junctions are shared across patients and present in developmental and other non-cancer cells. NAR Cancer.

[34] Nejo T., Wang L., Leung K.K., Wang A., Lakshmanachetty S., Gallus M., Kwok D.W., Hong C., Chen L.H., Carrera D.A. (2024). Challenges in the discovery of tumor-specific alternative splicing-derived cell-surface antigens in glioma. Sci. Rep..

[35] Camarena M.E., Theunissen P., Ruiz M., Ruiz-Orera J., Calvo-Serra B., Castelo R., Castro C., Sarobe P., Fortes P., Perera-Bel J. (2024). Microproteins encoded by noncanonical ORFs are a major source of tumor-specific antigens in a liver cancer patient meta-cohort. Sci. Adv..

[36] Li G., Mahajan S., Ma S., Jeffery E.D., Zhang X., Bhattacharjee A., Venkatasubramanian M., Weirauch M.T., Miraldi E.R., Grimes H.L. (2024). Splicing neoantigen discovery with SNAF reveals shared targets for cancer immunotherapy. Sci. Transl. Med..

[37] Parikh N.D., Mehta A.S., Singal A.G., Block T., Marrero J.A., Lok A.S. (2020). Biomarkers for the Early Detection of Hepatocellular Carcinoma. Cancer Epidemiol. Biomark. Prev..

[38] Zhu Y., Wang S., Xi X., Zhang M., Liu X., Tang W., Cai P., Xing S., Bao P., Jin Y. (2021). Integrative Analysis of Long Extracellular RNAs Reveals a Detection Panel of Noncoding RNAs for Liver Cancer. Theranostics.

[39] Chen S., Zhou Y., Chen Y., Gu J. (2018). fastp: An ultra-fast all-in-one FASTQ preprocessor. Bioinformatics.

[40] Kim D., Paggi J.M., Park C., Bennett C., Salzberg S.L. (2019). Graph-based genome alignment and genotyping with HISAT2 and HISAT-genotype. Nat. Biotechnol..

[41] Kim D., Langmead B., Salzberg S.L. (2015). HISAT: A fast spliced aligner with low memory requirements. Nat. Methods.

[42] Danecek P., Bonfield J.K., Liddle J., Marshall J., Ohan V., Pollard M.O., Whitwham A., Keane T., McCarthy S.A., Davies R.M. (2021). Twelve years of SAMtools and BCFtools. GigaScience.

[43] Li H., Handsaker B., Wysoker A., Fennell T., Ruan J., Homer N., Marth G., Abecasis G., Durbin R., 1000 Genome Project Data Processing Subgroup (2009). The Sequence Alignment/Map format and SAMtools. Bioinformatics.

[44] Cotto K.C., Feng Y.Y., Ramu A., Richters M., Freshour S.L., Skidmore Z.L., Xia H., McMichael J.F., Kunisaki J., Campbell K.M. (2023). Integrated analysis of genomic and transcriptomic data for the discovery of splice-associated variants in cancer. Nat. Commun..

[45] Yates A.D., Austine-Orimoloye O., Azov A.G., Barba M., Barnes I., Barrera-Enriquez V.P., Becker A., Bennett R., Berry A., Bhai J. (2026). Ensembl 2026. Nucleic Acids Res..

[46] Padfield E., Ellis H.P., Kurian K.M. (2015). Current Therapeutic Advances Targeting EGFR and EGFRvIII in Glioblastoma. Front. Oncol..

[47] MacDonald J.R., Ziman R., Yuen R.K., Feuk L., Scherer S.W. (2014). The Database of Genomic Variants: A curated collection of structural variation in the human genome. Nucleic Acids Res..

[48] Casper J., Speir M.L., Raney B.J., Perez G., Nassar L.R., Lee C.M., Hinrichs A.S., Gonzalez J.N., Fischer C., Diekhans M. (2026). The UCSC Genome Browser database: 2026 update. Nucleic Acids Res..

[49] Clopper C.J., Pearson E.S. (1934). The use of confidence or fiducial limits illustrated in the case of the binomial. Biometrika.

[50] Nemhauser G.L., Wolsey L.A., Fisher M.L. (1978). An analysis of approximations for maximizing submodular set functions—I. Math. Program..

[51] Hochbaum D.S., Hochbaum D.S. (1997). Approximating covering and packing problems: Set cover, vertex cover, independent set, and related problems. Approximation Algorithms for NP-Hard Problems.

[52] Mavrogiannou E., Strati A., Stathopoulou A., Tsaroucha E.G., Kaklamanis L., Lianidou E.S. (2007). Real-time RT-PCR quantification of human telomerase reverse transcriptase splice variants in tumor cell lines and non-small cell lung cancer. Clin. Chem..

[53] Yan T., Gao S., Peng X., Shi J., Xie C., Li Q., Lu L., Wang Y., Zhou F., Liu Z. (2015). Significantly decreased and more variable expression of major CYPs and UGTs in liver microsomes prepared from HBV-positive human hepatocellular carcinoma and matched pericarcinomatous tissues determined using an isotope label-free UPLC-MS/MS method. Pharm. Res..

[54] Jakobsson J., Ekström L., Inotsume N., Garle M., Lorentzon M., Ohlsson C., Roh H.K., Carlström K., Rane A. (2006). Large differences in testosterone excretion in Korean and Swedish men are strongly associated with a UDP-glucuronosyl transferase 2B17 polymorphism. J. Clin. Endocrinol. Metab..

[55] Yang T.L., Chen X.D., Guo Y., Lei S.F., Wang J.T., Zhou Q., Pan F., Chen Y., Zhang Z.X., Dong S.S. (2008). Genome-wide copy-number-variation study identified a susceptibility gene, UGT2B17, for osteoporosis. Am. J. Hum. Genet..

[56] Xue Y., Sun D., Daly A., Yang F., Zhou X., Zhao M., Huang N., Zerjal T., Lee C., Carter N.P. (2008). Adaptive evolution of UGT2B17 copy-number variation. Am. J. Hum. Genet..

[57] Ménard V., Eap O., Harvey M., Guillemette C., Lévesque E. (2009). Copy-number variations (CNVs) of the human sex steroid metabolizing genes UGT2B17 and UGT2B28 and their associations with a UGT2B15 functional polymorphism. Hum. Mutat..

[58] Dobin A., Davis C.A., Schlesinger F., Drenkow J., Zaleski C., Jha S., Batut P., Chaisson M., Gingeras T.R. (2013). STAR: Ultrafast universal RNA-seq aligner. Bioinformatics.

[59] Peng J.R., Chen H.S., Mou D.C., Cao J., Cong X., Qin L.L., Wei L., Leng X.S., Wang Y., Chen W.F. (2005). Expression of cancer/testis (CT) antigens in Chinese hepatocellular carcinoma and its correlation with clinical parameters. Cancer Lett..

[60] Zhao L., Mou D.C., Peng J.R., Huang L., Wu Z.A., Leng X.S. (2010). Diagnostic value of cancer-testis antigen mRNA in peripheral blood from hepatocellular carcinoma patients. World J. Gastroenterol..

[61] Grosso A.R., Leite A.P., Carvalho S., Matos M.R., Martins F.B., Vítor A.C., Desterro J.M., Carmo-Fonseca M., de Almeida S.F. (2015). Pervasive transcription read-through promotes aberrant expression of oncogenes and RNA chimeras in renal carcinoma. eLife.

[62] Varley K.E., Gertz J., Roberts B.S., Davis N.S., Bowling K.M., Kirby M.K., Nesmith A.S., Oliver P.G., Grizzle W.E., Forero A. (2014). Recurrent read-through fusion transcripts in breast cancer. Breast Cancer Res. Treat..

[63] Maher C.A., Palanisamy N., Brenner J.C., Cao X., Kalyana-Sundaram S., Luo S., Khrebtukova I., Barrette T.R., Grasso C., Yu J. (2009). Chimeric transcript discovery by paired-end transcriptome sequencing. Proc. Natl. Acad. Sci. USA.

[64] Hu D.G., Marri S., Hulin J.A., Ansaar R., Mackenzie P.I., McKinnon R.A., Meech R. (2024). Activation of Cryptic Donor Splice Sites within the UDP-Glucuronosyltransferase (UGT)1A First-Exon Region Generates Variant Transcripts That Encode UGT1A Proteins with Truncated Aglycone-Binding Domains. Drug Metab. Dispos..

[65] Killela P.J., Reitman Z.J., Jiao Y., Bettegowda C., Agrawal N., Diaz L.A., Friedman A.H., Friedman H., Gallia G.L., Giovanella B.C. (2013). TERT promoter mutations occur frequently in gliomas and a subset of tumors derived from cells with low rates of self-renewal. Proc. Natl. Acad. Sci. USA.

[66] Huang F.W., Hodis E., Xu M.J., Kryukov G.V., Chin L., Garraway L.A. (2013). Highly recurrent TERT promoter mutations in human melanoma. Science.

[67] Sung W.K., Zheng H., Li S., Chen R., Liu X., Li Y., Lee N.P., Lee W.H., Ariyaratne P.N., Tennakoon C. (2012). Genome-wide survey of recurrent HBV integration in hepatocellular carcinoma. Nat. Genet..

[68] Aran D., Camarda R., Odegaard J., Paik H., Oskotsky B., Krings G., Goga A., Sirota M., Butte A.J. (2017). Comprehensive analysis of normal adjacent to tumor transcriptomes. Nat. Commun..

[69] Jhuang H.Y., Wang L.T. (2026). Long-read discovery in cancer cell lines defines a cis-spliced fusion RNA panel and its clinical associations across TCGA. Cancer Genet..

[70] Mahajan M., Hemberg M. (2025). Detecting known neoepitopes, gene fusions, transposable elements, and circular RNAs in cell-free RNA. Bioinformatics.

[71] Nesselbush M.C., Luca B.A., Jeon Y.J., Jabara I., Meador C.B., Garofalo A., Binkley M.S., Hui A.B., van ’t Erve I., Xu N. (2025). An ultrasensitive method for detection of *cell*-free RNA. Nature.

[72] Parikh A.R., Van Seventer E.E., Siravegna G., Hartwig A.V., Jaimovich A., He Y., Kanter K., Fish M.G., Fosbenner K.D., Miao B. (2021). Minimal Residual Disease Detection using a Plasma-only Circulating Tumor DNA Assay in Patients with Colorectal Cancer. Clin. Cancer Res..

[73] Heydt C., Ihle M.A., Ilm K., Rehker J., Poon P., Siemanowski-Hrach J., Pappesch R., Wagener-Ryczek S., Jonas C., Fassunke J. (2026). Pitfalls in Detecting MET Exon 14 Skipping Variants by DNA- and RNA-Based Next-Generation Sequencing Technologies in a Large Real-World Cohort and Results of the First Multinational External Quality Assessment Schemes. J. Mol. Diagn..

[74] Hanley J.A., Lippman-Hand A. (1983). If nothing goes wrong, is everything all right? Interpreting zero numerators. JAMA.

[75] Eypasch E., Lefering R., Kum C.K., Troidl H. (1995). Probability of adverse events that have not yet occurred: A statistical reminder. BMJ.

[76] Vorperian S.K., Moufarrej M.N., Tabula Sapiens Consortium, Quake S.R. (2022). Cell types of origin of the cell-free transcriptome. Nat. Biotechnol..

