# Supplementary material for: Normal-Tissue Absence, Rather Than Cross-Patient Prevalence, Limits a Fixed Splice-Junction Panel in Hepatocellular Carcinoma: From a Pre-Specified In Silico Screen to a Genome-Wide Analysis
Source: Biomedicines. 2026 Oct 20;14(10):2396. doi: 10.3390/biomedicines14102396 (PMC13702348; doi:10.3390/biomedicines14102396)
Supplement: Supplementary file 1 [file biomedicines-14-02396-s001.zip › biomedicines-4603374-supplementary.pdf]

Supplementary Material

Normal-Tissue Absence, Rather Than Cross-Patient Prevalence, Limits a Fixed Splice-Junction Panel in Hepatocellular Carcinoma: From a Pre-Specified In Silico Screen to a Genome-Wide Analysis

Duygu Aygüneş-Jafari

Supplementary Section S1. Normal-reference comparison at the CFH locus

Four candidate explanations for the discrepancy between TCGA-LIHC matched normal and GTEx liver at the CFH to CFHR4 junction (chr1:196,690,240–196,905,107, plus strand; 47 of 50 matched normals, 0 of 251 GTEx liver, 214 of 374 tumours at the 2-read carrier definition) were tested. None accounted for the difference.

Detectability. GTEx liver reads are longer (median 152 bp against 96 bp in TCGA-LIHC), and GTEx junction coverage is 20% lower than that of the matched normals (medians 9.92 million GTEx, 10.43 million TCGA tumour, 12.36 million TCGA matched normal); a difference of this size does not account for 47 of 50 carriers against 0 of 251.

Paralogue mismapping. Both 100 bp junction flanks align to exactly one genomic location at zero mismatches, with no other alignment within five mismatches.

A CFHR3-CFHR1 deletion haplotype. Carriers of the junction have higher, not lower, CFHR3 and CFHR1 coverage than non-carriers (depth-normalised medians 3251 against 1753 reads per 10^6 panel junction reads, Mann–Whitney $p = 9.11 \times 10^{-21}$), the opposite of the direction expected under a deletion haplotype.

Locus-level tissue state. CFH-cluster coverage did not differ significantly between TCGA-LIHC matched normal (median 8485.2 reads per 10^6) and GTEx liver (median 7949.4; ratio 1.07, $p = 0.107$), in contrast to the genuine cohort-level offset observed for ALB in the same comparison (main text Table 1, Supplementary Figure S2). This test queried the CFH, CFHR1, CFHR2 and CFHR3 cluster; CFHR4, at the acceptor end of the junction, lies outside the panel (main text Section 2.3); the test therefore bears on the donor-side locus only.

The compilations record this junction, whose intron is 214.9 kb, only in samples carrying at least 4 reads (main text Section 3.6; Supplementary Table S12). The matched-normal count of 47 of 50 and the GTEx count of 0 of 251 are therefore counts at 4 or more reads, and a GTEx sample carrying 1 to 3 reads would not be recorded. A common read floor does not account for a junction carried at 4 or more reads by 47 of 50 matched normal samples and by none of 251 GTEx liver samples.

Supplementary Section S2. Motif case-sensitivity check and count definitions

The canonical-motif filter applied throughout matched upper case only, and therefore also excluded junctions whose splice sites fall within soft-masked (RepeatMasker) intervals, since Snaptron returns motif dinucleotides in the case of the underlying reference sequence.

The effect of the upper-case rule was quantified post hoc. Relaxing the match to be case-insensitive enlarges the panel-wide canonical set detected in at least one tumour from 8624 to 9936 junctions, and the eligible set from 981 to 1292 junctions (1178 unannotated, 114 annotated, against 894 and 87 under the uppercase-only rule). Under the case-insensitive rule, the following quantities are unchanged or change by less than one percentage point: the median tumour prevalence of the eligible set remains 1 of 374; the proportion detected in exactly one tumour is 75.4% against 74.8%; the number of eligible junctions exceeding 30 of 374 tumours remains 15; the highest prevalence in the eligible set remains 88 of 374; and the number for which absence from matched normal tissue can be established at the criterion of main text Section 3.4 remains 8. The main text is reported on the uppercase-only set

of 981, except for the recurrence spectrum and threshold sweep of Figure 2 and Section 3.6 (Supplementary Table S1), which are computed on the case-insensitive set of 1292.

The evidence floor of main text Section 3.1 (12,783 junctions) was computed with a case-insensitive motif match on the tcgav2 motifs. Under the upper-case rule, with motifs taken from either compilation, it comprises 11,069 junctions. The reference-concordance analysis of Supplementary Section S5 reports both.

The junction and gene counts used in this report are defined in Supplementary Table S7.

Supplementary Table S7. Junction and gene counts used in this report, and the filter each is computed under.

Count	Definition	Where used
1,263,305	All junctions returned by the per-gene Snaptron queries, before any filter	Section 3.1
1,002,937	Of all queried junctions, those assigned to a queried gene by start coordinate	Section 3.1
3314	Meeting the recurrence floor, before the canonical-motif restriction	Sections 3.1 and 3.6
3163	The canonical-motif subset of the 3314	Main Table 6
3155	Of the 3,163, those assigned by coordinate containment, otherwise overlap (99.7%)	Main Table 5
981	Canonical, normal-absent junctions detected in at least one tumour under the uppercase-only motif rule; the eligible set used throughout the main text	Sections 3.3 to 3.6
1292	The same set under a case-insensitive motif match	Supplementary Section S2, Supplementary Table S1, Figure 2
8	Of the 981, those whose prevalence permits absence from matched normal tissue to be supported at the criterion of main text Section 3.4	Sections 3.4 to 3.6
300	Panel slots, all distinct gene symbols	Section 2.3
296	Of the 300, those queried: 300 less 4 symbols unresolved against GENCODE v26; 13 of the 296 are mitochondrially encoded and not spliced	Section 2.3
12,783	Canonical junctions (case-insensitive match on tcgav2 motifs) with at least 5 reads in at least one sample: the evidence floor	Sections 3.1 and 3.2
11,069	The evidence floor under the upper-case motif rule, motif from either compilation	Section 3.1, Supplementary Section S5
22	Of the 981, those reaching the informative prevalence of the GTEx criterion (at least 19 of 374)	Section 3.4, Main Table 3
25	Of the 981, those reaching the informative prevalence of the two references jointly (at least 15 of 374)	Section 3.4, Main Table 3
320	Of the 981, those with an intron longer than 100 kb, for which samples with fewer than 3 or 4 reads are not recorded	Section 3.6, Supplementary Table S12

Supplementary Section S3. Plasma cell-free RNA feasibility check

S3.0 Methods

Numbers in square brackets in this document refer to the reference list of the main text: fastp [39], HISAT2 [40,41], SAMtools [42,43] and regtools [44].

Data. Plasma cell-free RNA sequencing data were obtained from the European Nucleotide Archive, study SRP239389 (Gene Expression Omnibus series GSE142987), a publicly consented paired-end cfRNA sequencing resource that includes libraries from patients with liver cancer and from healthy donors. Run-level metadata were retrieved from the ENA file report interface. The study comprises 65 sequencing runs; the ENA sample_title field was used to classify each run as liver cancer or healthy donor, assigning 35 runs to liver cancer patients and 30 to healthy donors (SRP239389_titles.tsv).

Library selection. Four cancer libraries were selected for per-library processing by combined FASTQ file size, smallest first: SRR10822542 (2.31 GB, liver_cancer_3), SRR10822548 (3.66 GB, liver_cancer_9), SRR10822572 (3.77 GB, liver_cancer_33) and SRR10822570 (3.89 GB, liver_cancer_31). The selection rule was fixed before any quality metric was computed. SRR10822542, the smallest, was designated in advance as the primary library on which the sensitivity criterion of main text Section 2.4 is defined; the other three cancer libraries form a post-hoc extension. One healthy-donor library (SRR10822600, 20,146,526 read pairs after trimming) was processed as a same-chemistry, opposite-condition contrast.

Quality control. Read-level quality metrics were produced with fastp 1.3.6 for each processed library. Metrics fastp does not provide were computed separately for each library: strandedness was inferred from a one-million-pair subsample aligned against the annotation, and rRNA and globin fractions were obtained by counting subsample reads matching rRNA and HBA/HBB sequences. No numeric QC cut-off was pre-specified; QC is reported descriptively, and no library was excluded on QC grounds.

Control sets. Negative controls were annotated junctions present in both tumours and normals at comparable rates, as defined in main text Section 2.4. Sensitivity controls were canonical junctions in eight liver-abundant, plasma-plausible genes (ALB, SERPINA1, APOA1, FGA, FGB, FGG, HP, TF), which serve as high-abundance process controls. Specificity controls were three splice junctions characteristic of other tissues, expected to be absent from liver: a MET exon 13-to-15 skip corresponding to the clinically recognised exon 14 skipping event, a BRAF exon 3-to-9 skip, and an AR-V7-like unannotated junction at the GENCODE v26 AR exon 3 donor (not a curated AR-V7 coordinate, since the AR-V7 cryptic exon 3 is not annotated in GENCODE v26). Their absence is reported as expected behaviour and is not treated as evidence that the method functions.

Junction sequences and genome-wide uniqueness. For each candidate, 100 bp of GRCh38 sequence flanking each side of the junction were extracted and joined to form a 200 bp junction-spanning reference, together with the corresponding unspliced genomic decoy. Donor and acceptor dinucleotides recomputed from the genome sequence agreed with the motifs reported by Snaptron for 57 of 57 candidates carrying a reported motif (32 CT..AC on the minus strand, 25 GT..AG on the plus strand). Every junction-straddling 31-mer set was screened for uniqueness against GRCh38, and candidates whose straddling k-mers were not unique were removed. The screen was an exact genome-wide k-mer scan, which recovered 100% of its decoy controls. One negative control was removed by the screen; no sensitivity or specificity control was affected. Twelve of 72 candidates (10 negative and 2 sensitivity controls) were excluded from the k-mer analyses because their introns are shorter than the 200 bp decoy window. The screen tests, for each junction, the 16 junction-straddling 31-mers at offsets 77 to 92 of the 200 bp junction-spanning reference, which place between 8 and 23 bp on either side of the junction; it therefore evaluates genome-wide uniqueness down to the pre-specified 8 bp anchor, for exact matches only.

Junction counting. Two counting methods were applied. Alignment-based counting (Method 1) used HISAT2 2.2.3 against the prebuilt grch38_tran index, followed by SAMtools 1.24 and junction extraction with regtools at the pre-specified 8 bp anchor. The prebuilt index carries Ensembl release 84 splice-site priors on the GRCh38 primary assembly; contig names were translated explicitly, and the 351,372 GENCODE v26 splice sites were supplied at alignment time. Alignment-free counting (Method 2) streamed the FASTQ files and performed exact 31-mer matching against the junction-spanning references and their unspliced decoys, requiring each k-mer to straddle the junction with the pre-specified anchor on both sides and scanning both strands. This method was run for the four cancer libraries; the healthy-donor contrast library was counted by Method 1 only. Agreement between the two methods was assessed as a Spearman correlation on the primary library's candidate junction set and as agreement in gene-detection calls in each of the four cancer libraries (Section S3.2).

Read attrition and pooling. A read-attrition funnel was computed for each of the four cancer libraries from five quantities recorded in sequence: pairs sequenced, pairs after trimming, primary alignments, primary alignments with a spliced (CIGAR-N) record, and primary spliced alignments falling within a panel gene. The final funnel stage,

reads at the sensitivity-control junctions specifically, was taken directly from the Method 1 per-junction counts summed over the eight sensitivity-control genes. Per-run Method 1 junction counts were also summed across the four cancer libraries (100,539,319 read pairs combined). A separate cancer-versus-healthy-donor contrast compared the four cancer libraries against the one healthy-donor library without pooling. Both analyses are post hoc.

S3.1 Pre-specified outcome

The pre-specified sensitivity criterion is defined on a single library, SRR10822542 (plasma cfRNA, patient liver_cancer_3): detection of at least 6 of the 8 sensitivity-control genes by Method 1 (HISAT2, regtools junction extraction, 8 bp anchor). That library reached 3 of 8. The pre-specified criterion was therefore not met.

The three further cancer libraries and the healthy-donor library (Section S3.0) form a post-hoc extension and do not re-evaluate the pre-specified criterion.

Strandedness was inferred as RF (fr-firststrand) in all four cancer libraries.

S3.2 Per-library detection

Of the 4 cancer libraries, 2 detected all 8 control genes, 1 detected 5 of 8, and the primary library, on which the pre-specified criterion is defined, detected 3 of 8 (Supplementary Table S5, Supplementary Figure S5b). The one healthy-donor library reached 1 of 8. Method 1 and Method 2 returned identical gene-detection calls in each of the four cancer libraries, and on the primary library's candidate set the two methods correlate with Spearman rho = 0.975 ($p = 7.52 \times 10^{-39}$, n = 59 junctions); Method 2 was not run on the healthy-donor library (Supplementary Table S5). Both methods draw on the same fragment pool; their agreement establishes counting consistency, not independent confirmation of detection.

Supplementary Table S5. Sensitivity controls, per library; pre-specified criterion 6 of 8.

Library	Sample	Condition	Method 1	Method 2	Genes detected (Method 1)
SRR10822548	cancer_9	cancer	8 of 8	8 of 8	ALB, APOA1, FGA, FGB, FGG, HP, SERPINA1, TF
SRR10822572	cancer_33	cancer	8 of 8	8 of 8	ALB, APOA1, FGA, FGB, FGG, HP, SERPINA1, TF
SRR10822570	cancer_31	cancer	5 of 8	5 of 8	ALB, APOA1, FGA, HP, SERPINA1
SRR10822542	cancer_3	cancer	3 of 8	3 of 8	ALB, FGA, SERPINA1
SRR10822600	healthy_26	healthy	1 of 8	not run	SERPINA1

The primary library is the lowest of the four cancer libraries in read pairs after trimming, alignment rate, exonic base fraction and spliced-alignment fraction and the highest in globin fraction (Supplementary Table S2, Supplementary Figure S5c).

S3.3 Determinants of detection

Among the three sensitivity-control genes detected in all four cancer libraries, per-gene Method 1 maxima vary 13.4-fold for ALB (49 to 655 reads), 2.8-fold for SERPINA1 (208 to 590) and 11.1-fold for FGA (14 to 155), and each of the other five genes is undetected in at least one library; this variation exceeds the 2.2-fold range in sequencing depth across the same libraries (14.5 to 31.4 million read pairs after trimming, Supplementary Table S2).

The read-attrition funnel shows the same pattern at an earlier stage (Supplementary Figure S5a). Expressing the weakest library (SRR10822542) as a fraction of the four-library median at each stage gives 1.9-fold below median at reads sequenced, 2.4-fold at primary alignments, 3.1-fold at spliced primary alignments, 7.2-fold at spliced alignments within a panel gene, and 1.8-fold at reads reaching a sensitivity-control junction. The deficit widens through alignment and splicing and peaks at the panel-gene stage, before the sensitivity-control junctions are reached, exceeding the 1.9-fold deficit in reads sequenced. At the final stage the library contributes reads at three junctions rather than eight, and at those three its counts lie close to the median (0.69 for ALB, 0.67 for SERPINA1,

1.74 for FGA), a largest per-gene shortfall of 1.5-fold against 7.2-fold at the panel-gene stage (Supplementary Table S3). The loss in this library is predominantly in the number of junctions reaching detection, not in the depth at those it does reach.

Across the four cancer libraries, the number of sensitivity-control genes detected tracks the QC subsample's spliced-alignment fraction (Spearman rho = +0.949) more closely than it tracks the number of read pairs after trimming (rho = +0.316) (Supplementary Figure S5c). With n = 4, these are descriptive rank correlations; no p-value was computed.

rRNA fraction does not track detection outcome: the two libraries with rRNA fractions 5.8- to 8.0-fold higher than the other two (0.1534 and 0.1278 against 0.0221 and 0.0192) detect 5 and 8 sensitivity-control genes, against 3 and 8 (Supplementary Table S2, Supplementary Table S5).

Summed across the four cancer libraries (100,539,319 read pairs), Method 1 counts detect all 8 genes, which is by construction the union of the per-library calls and carries no information on depth. The comparison between individual libraries is informative: the deepest library, SRR10822570 (31,417,387 read pairs after trimming), detects 5 of 8 genes, whereas SRR10822548 (24,783,270 read pairs) detects 8 of 8, consistent with library composition rather than sequencing depth setting the detection ceiling in these four libraries.

The healthy-donor library (SRR10822600, 20,146,526 read pairs, deeper than the weakest cancer library at 14,520,533 read pairs) returns zero reads at every sensitivity-control locus except SERPINA1, where it reaches 10 reads against a cancer-library median of 310.0 (Supplementary Table S6). With one healthy-donor library, this is a descriptive observation, not a condition comparison.

Negative controls are detected at 35 to 44 of 45 junctions in the four cancer libraries, in the same rank order as the sensitivity controls, and at 30 of 45 in the healthy-donor library; the three specificity controls are absent from all five libraries (Supplementary Table S4).

With four cancer libraries selected by file size and one healthy-donor library, the check supports no general statement about plasma cfRNA in either direction. The post-hoc four-library extension is descriptive: a 2.2-fold range in depth accompanies a 6.2- to 11.9-fold difference in spliced alignments within panel genes relative to the primary library and a 2.8- to 13.4-fold range in per-gene Method 1 maxima among the genes detected in all four libraries.

Supplementary Table S6. Cancer libraries versus the healthy-donor library.

Gene	Cancer libraries detected	Cancer median of per-library maxima	Healthy-donor library maximum
ALB	4 of 4	179.5	0
SERPINA1	4 of 4	310.0	10
FGA	4 of 4	89.0	0
APOA1	3 of 4	6.5	0
HP	3 of 4	8.0	0
FGB	2 of 4	23.0	0
FGG	2 of 4	6.0	0
TF	2 of 4	13.5	0

Supplementary Section S4. Data provenance by table and section

The mapping below records the underlying data file from which each table and figure is drawn.

Item	Source file(s)
Main Table 1	cfh_tissue_state.tsv
Main Table 2	paired_normal_test.tsv

Item	Source file(s)
Main Table 3	false_absence_power.tsv, eligible_absence_evidence.tsv, false_absence_summary.txt
Main Table 4	set_cover_curves.tsv, set_cover_saturation.tsv, set_cover_validation.tsv, false_absence_supported_coverage.tsv, set_cover_summary.txt
Main Table 5	panel_selection_sensitivity.tsv, gene_panel.tsv, gene_coords.tsv
Main Table 6	recurrence_floor_sweep.tsv
Supplementary Table S1	threshold_sweep.tsv, threshold_sweep_summary.txt
Supplementary Table S2	per_library_qc.tsv
Supplementary Table S3	per_library_sensitivity.tsv (m1_max column)
Supplementary Table S4	per_library_summary.txt
Supplementary Table S5	per_library_sensitivity.tsv (Method 2 fields for SRR10822600 are zero-filled placeholders; Method 2 was not run on that library)
Supplementary Table S6	cancer_vs_healthy.tsv, per_library_summary.txt
Supplementary Table S7	filter_attrition.tsv, motif_case_sensitivity.tsv, recurrence_floor_sweep.tsv, panel_selection_sensitivity.tsv, gene_panel.tsv
Figure 1	filter_attrition.tsv via 51_split_figures.py
Figure 2	recurrence_spectrum_by_annotation.tsv, threshold_sweep.tsv
Figure 3	tert_expression.tsv, tert_carriers_canonical.tsv
Figure 4	chr4_junction_counts.tsv, cohort_depth.tsv
Figure 5	eligible_absence_evidence.tsv via 51_split_figures.py
Figure 6	set_cover_curves.tsv, set_cover_saturation.tsv, set_cover_validation.tsv, set_cover_per_patient.tsv
Supplementary Figure S1	motif_composition.tsv
Supplementary Figure S2	cfh_tissue_state.tsv, cfh_locus_summary.txt, cfh_tissue_state_summary.txt
Supplementary Figure S3	paired_normal_test.tsv
Supplementary Figure S4	false_absence_supported_coverage.tsv, normal_side_sweep.tsv via 51_split_figures.py
Supplementary Figure S5	detection_funnel.tsv, per_library_sensitivity.tsv, per_library_qc.tsv via 51_split_figures.py
Section 3.1, filtering cascade and pre-specified thresholds	filter_attrition.tsv, thresholds.json, motif_composition.tsv
Section 3.2, CFH to CFHR4 GTEx count (0 of 251)	junctions_gtexv2.tsv.gz (junction chr1:196,690,240–196,905,107, plus strand)
Supplementary Section S1	cfh_tissue_state.tsv, cfh_locus_summary.txt, cfh_carrier_status.tsv, cfh_paralogue_hits.tsv, chr4_junction_counts.tsv
Section 3.3, annotated/unannotated split	recurrence_spectrum.tsv, recurrence_spectrum_by_annotation.tsv, motif_case_sensitivity.tsv
Section 3.3, GNAS/RPL22 query	posthoc_gnas_rpl22_junctions.tsv, posthoc_positive_control_summary.txt
Section 3.3.1, TERT locus	tert_junctions.tsv, tert_junctions_canonical.tsv, tert_expression.tsv, tert_carriers_canonical.tsv, tert_summary.txt, tert_expression_summary.txt, tert_canonical_set_summary.txt

Item	Source file(s)
Section 3.3.2, UGT2B locus	chr4_junction_counts.tsv, ugt2b_locus_direction.tsv, ugt_flank_uniqueness.tsv, srav3h_chr4_junction.txt, cohort_depth.tsv, junction_splice_site_verification.tsv
Sections 3.3 to 3.6, eligible set (981)	junctions_tcgav2.tsv.gz, junctions_gtexv2.tsv.gz via 36_set_cover.py
Section 3.6, matched-normal burden of the 3314	junctions_tcgav2.tsv.gz, junctions_gtexv2.tsv.gz (recomputed directly; not summarised in a derived table)
Section 3.6, threshold and normal-side sweeps	threshold_sweep.tsv, threshold_sweep_summary.txt, normal_side_sweep.tsv
Supplementary Section S3	per_library_qc.tsv, per_library_sensitivity.tsv, per_library_summary.txt, pooled_cancer_sensitivity.tsv, cancer_vs_healthy.tsv, detection_funnel.tsv, detection_funnel_summary.txt, method_correlation.tsv
Main Table 3, rows for the GTEx and joint criteria; Section 3.4	reference_power.tsv via 62_reference_power.py
Main Table 7	normal_floor_full_sweep.tsv via 73_normal_floor_full_sweep.py (per-sample counts from 71_recount3_extract.py); normal_floor_sensitivity.tsv, normal_floor_gained_junctions.tsv via 60_normal_floor_sensitivity.py
Figure 5b	closed form and binomial distribution, no data file; 51_split_figures.py
Section 3.2, panel-wide reference concordance; Supplementary Figure S6; Supplementary Table S11	reference_concordance.tsv, reference_concordance_bins.tsv via 61_reference_concordance.py
Section 3.3, positive control; Supplementary Table S8	positive_control_junctions.tsv via 63_positive_control.py
Sections 3.3 and 3.3.1, tissue distribution; Supplementary Table S9	supported_junctions_gtex_tissues.tsv, supported_junctions_gtex_summary.tsv via 64_tissue_distribution.py
Section 3.3.2, UGT2B junction across TCGA projects, the SRA compilation and locus annotation; Supplementary Table S10	ugt2b_junction_tcga_projects.tsv, ugt2b_junction_sra.tsv, ugt2b_locus_annotation.tsv via 65_ugt2b_locus.py
Section 3.6, read floor by intron length; Supplementary Table S12	long_intron_read_floor.tsv, long_intron_class_counts.tsv via 66_long_intron_read_floor.py
Section 3.6, normal-side carriers by read floor; Supplementary Table S13	supported_normal_floor_sweep.tsv via 67_supported_normal_floor_sweep.py
Section 3.3, junctions matching the published GNAS and RPL22 descriptions	described_neojunction_candidates.tsv via 68_described_neojunctions.py
Section 3.3.2, copy number of the UGT2B junction interval; Supplementary Table S14	ugt2b_copy_number_samples.tsv, ugt2b_copy_number_summary.tsv via 69_ugt2b_copy_number.py; ugt2b28_copy_number_samples.tsv, ugt2b28_copy_number_summary.tsv, ugt2b28_copy_number.txt via 75_ugt2b28_copy_number.py
Section 2.8, splice sites of the UGT2B junction in GENCODE v26	junction_splice_site_gencode_v26.tsv, gencode_v26_splice_sites.txt via 70_gencode_v26_splice_sites.py
Sections 2.10 and 3.6, recount3 per-sample counts and their agreement with the Snaptron tables; recording minimum by intron length	recount3_validation.tsv, recount3_extract.txt via 71_recount3_extract.py

Item	Source file(s)
Section 3.2, genome-wide reference concordance; Supplementary Figure S7; Supplementary Table S15	reference_concordance_genome.tsv, reference_concordance_genome_bins.tsv via 72_reference_concordance_genome.py
Sections 2.11 and 3.5, nested and patient-grouped split-half evaluation; Supplementary Table S16	set_cover_nested.tsv, set_cover_nested_subsets.tsv via 74_set_cover_nested.py
Sections 2.10 and 3.7, genome-wide application of the pre-specified cascade; Table 8; Supplementary Table S17	genome_wide_cascade_stages.tsv, genome_wide_candidates.tsv, genome_wide_candidate_tcga_projects.tsv, genome_wide_candidate_gtex_tissues.tsv, genome_wide_cascade.txt via 76_genome_wide_cascade.py

Supplementary Section S5. Additional reference and control analyses

The analyses in this section are post hoc. Methods are given in main text Sections 2.9 to 2.11. Every table is derived from the deposited data file named in Supplementary Section S4.

S5.1 Positive-control junctions

Two established oncogenic junctions, EGFRvIII in glioblastoma [46] and MET exon 14 skipping in lung adenocarcinoma [14,15], were retrieved from the same compilations by exact-coordinate query and counted at the same read floors as the main screen (5 reads in tumour samples, 2 reads in normal samples). Both were recovered in the tumour type in which they are known to occur and were returned as absent from the corresponding normal references under the criteria of this study (no matched-normal carrier, and GTEx carriers within the 1% tolerance): EGFRvIII was carried by 0 of 5 glioblastoma solid-tissue normal samples and 0 of 2931 GTEx brain samples, and MET exon 14 skipping by 0 of 59 lung adenocarcinoma solid-tissue normal samples and by 1 of 655 GTEx lung samples at 2 or more reads (Supplementary Table S8). In other TCGA projects, EGFRvIII was carried at 5 or more reads by LGG 11 of 532; HNSC 1 of 502; STAD 1 of 416 tumours, and MET exon 14 skipping by LUSC 2 of 504; LGG 1 of 532. The EGFRvIII intron is 136 kb, and samples carrying fewer than 3 reads are therefore not recorded for it (Supplementary Section S5.5); the junction was not returned by the GTEx compilation in any tissue. The flanking junctions show that both loci are expressed in the normal references, and absence of the skip junctions there is consequently informative.

Supplementary Table S8. Positive-control junctions and their flanking junctions (post hoc). Carriers are samples with at least 1, 2 or 5 reads; the main-screen floors are 5 reads for tumour samples and 2 reads for normal samples.

Junction	Intron (GRCh38)	Group	Samples	≥ 1 read	≥ 2 reads	≥ 5 reads	Maximum reads
EGFRvIII: exon1-exon2 (flank)	chr7:55,019,366–55,142,285	TCGA-GBM tumours	170	151	151	137	2875
EGFRvIII: exon1-exon2 (flank)	chr7:55,019,366–55,142,285	TCGA-GBM solid tissue normals	5	1	1	0	3
EGFRvIII: exon1-exon2 (flank)	chr7:55,019,366–55,142,285	GTEx Brain	2931	587	587	206	28
EGFRvIII: exon1-exon2 (flank)	chr7:55,019,366–55,142,285	GTEx all	19,214	12,272	12,272	9695	176
EGFRvIII: exon7-exon8 (flank)	chr7:55,154,153–55,155,829	TCGA-GBM tumours	170	169	167	161	5891

Junction	Intron (GRCh38)	Group	Samples	≥ 1 read	≥ 2 reads	≥ 5 reads	Maximum reads
EGFRvIII: exon7-exon8 (flank)	chr7:55,154,153–55,155,829	TCGA-GBM solid tissue normals	5	4	3	2	6
EGFRvIII: exon7-exon8 (flank)	chr7:55,154,153–55,155,829	GTEx Brain	2931	2207	1604	536	37
EGFRvIII: exon7-exon8 (flank)	chr7:55,154,153–55,155,829	GTEx all	19,214	16,792	15,543	12,334	340
EGFRvIII: exon1-exon8 (EGFRvIII skip)	chr7:55,019,366–55,155,829	TCGA-GBM tumours	170	34	34	30	341
EGFRvIII: exon1-exon8 (EGFRvIII skip)	chr7:55,019,366–55,155,829	TCGA-GBM solid tissue normals	5	0	0	0	0
EGFRvIII: exon1-exon8 (EGFRvIII skip)	chr7:55,019,366–55,155,829	GTEx Brain	2931	0	0	0	0 (not returned)
EGFRvIII: exon1-exon8 (EGFRvIII skip)	chr7:55,019,366–55,155,829	GTEx all	19,214	0	0	0	0 (not returned)
MET exon 14: exon13-exon14 (flank)	chr7:116,771,655–116,771,848	TCGA-LUAD tumours	542	532	513	476	2714
MET exon 14: exon13-exon14 (flank)	chr7:116,771,655–116,771,848	TCGA-LUAD solid tissue normals	59	59	59	58	64
MET exon 14: exon13-exon14 (flank)	chr7:116,771,655–116,771,848	GTEx Lung	655	598	525	358	123
MET exon 14: exon13-exon14 (flank)	chr7:116,771,655–116,771,848	GTEx all	19,214	14,672	12,672	8518	292
MET exon 14: exon14-exon15 (flank)	chr7:116,771,990–116,774,880	TCGA-LUAD tumours	542	518	506	466	1985
MET exon 14: exon14-exon15 (flank)	chr7:116,771,990–116,774,880	TCGA-LUAD solid tissue normals	59	59	59	59	52
MET exon 14: exon14-exon15 (flank)	chr7:116,771,990–116,774,880	GTEx Lung	655	609	569	415	104
MET exon 14: exon14-exon15 (flank)	chr7:116,771,990–116,774,880	GTEx all	19,214	15,164	13,597	9885	276
MET exon 14: exon13-exon15 (exon 14 skip)	chr7:116,771,655–116,774,880	TCGA-LUAD tumours	542	23	17	15	337

Junction	Intron (GRCh38)	Group	Samples	≥ 1 read	≥ 2 reads	≥ 5 reads	Maximum reads
MET exon 14: exon13-exon15 (exon 14 skip)	chr7:116,771,655–116,774,880	TCGA-LUAD solid tissue normals	59	3	0	0	1
MET exon 14: exon13-exon15 (exon 14 skip)	chr7:116,771,655–116,774,880	GTEx Lung	655	4	1	0	2
MET exon 14: exon13-exon15 (exon 14 skip)	chr7:116,771,655–116,774,880	GTEx all	19,214	25	4	0	3

The neojunctions reported at GNAS and RPL22 [18] are published without coordinates, as alternative 3' splice-site events that remove two and six nucleotides respectively. The junctions retrieved for the two loci were searched for a junction that shares its donor with a canonical junction carried by at least 112 of 374 tumours and whose acceptor is displaced by exactly 2 nt (GNAS) or 6 nt (RPL22). One candidate was found in GNAS, chr20:58,910,402–58,910,684 (unannotated): carried at 2 or more reads by 69 of 374 tumours (3 at 5 reads), by 3 of 50 matched normal samples and by 27 of 251 GTEx liver samples. Two candidates were found in RPL22, chr1:6,193,049–6,197,651 (15 tumours, 1 matched normal and 7 GTEx liver samples at 2 or more reads) and chr1:6,197,751–6,199,561 (8 tumours, 1 matched normal and 4 GTEx liver samples at 2 or more reads). None meets the absence criteria.

S5.2 Tissue distribution of the eight absence-supported junctions

The eight junctions were retrieved from the GTEx compilation and their carriers tabulated across all 19,214 samples (Supplementary Table S9). None is confined to tumour tissue when tissues other than liver are considered. The five TERT junctions are carried at 2 or more reads by 734 to 1915 GTEx samples, most frequently in testis, and by 11 to 45 of 852 whole-blood samples. The two UGT1A junctions are carried predominantly by small intestine, colon and stomach, and the UGT2B readthrough by 11 samples in five tissues; none of these three is carried by a whole-blood sample. The two UGT1A junctions and the UGT2B readthrough have introns longer than 100 kb, and for them counts at 1 and 2 reads equal counts at 3 reads (Supplementary Section S5.5).

Supplementary Table S9. Tissue distribution of the eight absence-supported junctions in GTEx (post hoc). The GTEx samples labelled Bone Marrow are the K-562 chronic myeloid leukaemia cell line. GTEx carriers are samples with at least 2 reads among 19,214; liver is of 251 samples and whole blood of 852.

Junction	Locus	Tumours ≥ 5 reads (of 374)	GTEx carriers	Tissues with a carrier	Three tissues with the highest carrier fraction	Liver, ≥ 1 / ≥ 2 reads	Whole blood, ≥ 1 / ≥ 2 / ≥ 5 reads
chr5:1254506–1255286	TERT	52	1177	21	Testis 368/410; Bone Marrow 90/204; Small Intestine 43/193	1 / 0	86 / 19 / 2
chr5:1255412–1258597	TERT	69	1915	22	Testis 392/410; Bone Marrow 157/204; Small Intestine 97/193	5 / 0	150 / 45 / 4
chr5:1258660–1260473	TERT	71	1756	20	Testis 389/410; Bone Marrow 149/204; Small Intestine 86/193	2 / 0	144 / 38 / 5

Junction	Locus	Tumours ≥ 5 reads (of 374)	GTEx carriers	Tissues with a carrier	Three tissues with the highest carrier fraction	Liver, ≥ 1 / ≥ 2 reads	Whole blood, ≥ 1 / ≥ 2 / ≥ 5 reads
chr5:1260601–1264403	TERT	57	734	17	Testis 263/410; Bone Marrow 127/204; Spleen 49/255	0 / 0	54 / 11 / 1
chr5:1266536–1268519	TERT	69	1021	18	Testis 331/410; Bone Marrow 139/204; Small Intestine 50/193	4 / 0	62 / 12 / 1
chr2:233618563–233767033	UGT1A	44	871	13	Small Intestine 127/193; Bladder 9/21; Colon 298/822	0 / 0	0 / 0 / 0
chr2:233637378–233767033	UGT1A	61	1267	16	Small Intestine 145/193; Stomach 161/384; Colon 330/822	0 / 0	0 / 0 / 0
chr4:69005136–69204429	UGT2B11 to UGT2B27P	88	11	5	Breast 5/482; Liver 1/251; Thyroid 2/706	1 / 1	0 / 0 / 0

S5.3 The UGT2B11 to UGT2B27P junction outside the liver cohorts

Across TCGA, the junction (chr4:69,005,136–69,204,429, minus strand) is carried by 336 samples, each with at least 3 reads, which is the minimum recorded for an intron of this length (Supplementary Section S5.5). Carriers are concentrated in breast and liver: 161 of 1142 breast tumours and 33 of 114 breast solid-tissue normal samples, and 114 of 374 liver tumours and 0 of 50 liver solid-tissue normal samples (Supplementary Table S10a; projects without a carrier are omitted). The junction is therefore present in normal breast tissue. In the srav3h compilation it is carried by 1023 of 316,447 samples from 215 studies, 658 of them at 5 or more reads. That compilation has no curated tissue annotation. A case-insensitive keyword search of the free-text sample and study fields assigns 321 carriers to liver-related samples (liver, hepat, HCC, HepG2, Huh) and 1 further carrier to blood-related samples, leaving 701 unassigned; the two studies contributing most carriers are of prostate cancer (203 and 75 carriers).

Supplementary Table S10a. Carriers of the UGT2B11 to UGT2B27P junction by TCGA project and sample type (post hoc). Tumour samples are those with a sample-type code below 10 and normal samples those with a code of 10 to 19 in the TCGA barcode.

Project	Tumour samples	Tumour carriers, ≥ 3 reads	Tumour carriers, ≥ 5 reads	Normal samples	Normal carriers, ≥ 3 reads	Normal carriers, ≥ 5 reads
BRCA	1142	161	135	114	33	21
LIHC	374	114	88	50	0	0
LUAD	542	8	5	59	0	0
STAD	416	6	5	37	0	0
CHOL	36	4	2	9	0	0
PAAD	179	4	3	4	0	0
ESCA	185	1	0	13	0	0
LUSC	504	1	1	51	0	0
PRAD	506	1	0	52	0	0

Project	Tumour samples	Tumour carriers, ≥ 3 reads	Tumour carriers, ≥ 5 reads	Normal samples	Normal carriers, ≥ 3 reads	Normal carriers, ≥ 5 reads
SARC	263	1	0	2	0	0
HNSC	504	0	0	44	1	0
THCA	513	0	0	59	1	0

Both splice sites lie within annotated segmental duplications (Supplementary Table S10b). The acceptor lies in six duplications and the donor in five, with 90.2% to 95.9% identity to their partners; one pair consists of the segment containing the acceptor and the segment containing the donor (92.3% identity). The 100 bp flanks used for the uniqueness test of main text Section 3.3.2 lie in the same duplications. The Database of Genomic Variants [47] records 18 structural variants overlapping the acceptor, 26 overlapping the donor and 105 overlapping the junction interval (deletion 34, duplication 20, loss 13, gain 11, alu insertion 8, insertion 6, sequence alteration 5, gain+loss 4, line1 insertion 1, alu deletion 1, complex 1, inversion 1). One alternate-haplotype sequence, chr4_GL000257v2_alt (chr4:68,306,359–69,010,457 on the primary assembly), covers the acceptor and not the donor; no fix patch lies in the region. Of the UGT2B genes (positions from Ensembl release 116 [45]), UGT2B7 and UGT2B26P lie inside the junction interval, UGT2B27P and UGT2B11 overlap its two ends, UGT2B17, UGT2B15 and UGT2B10 lie 378, 334 and 169 kb below it, and UGT2B28 and UGT2B4 lie 76 and 274 kb above it. These annotations establish that the region is structurally variable and paralogous.

Copy number of the junction interval was examined in open-access Affymetrix SNP 6.0 segments for TCGA-LIHC (Genomic Data Commons, GRCh38: 379 tumour, 306 blood-derived normal and 83 solid-tissue normal DNA samples; and, for tumour DNA, the Xena hub segments on hg19 that retain germline copy-number probes). The interval lies within segments of about 1 Mb, and an estimated 114 probes fall inside it. As a positive check, the UGT2B17 germline deletion is visible in the same data: 107 of 306 blood-derived normal samples (35.0%) have a log2 ratio below -0.3 at UGT2B17 and 73 (23.9%) below -1.0. The common UGT2B28 deletion [57] is also visible: 77 of 306 blood-derived normal samples (25.2%) have a log2 ratio below -0.3 at UGT2B28 and 5 (1.6%) below -1.0, on focal segments of median 104 kb (interquartile range 101 to 108 kb) that end a median 57.5 kb from the junction interval; one blood-derived sample, from a non-carrier, carries a single loss segment spanning both (458 kb). Germline loss of UGT2B28 or UGT2B17 is not significantly associated with carrier status (UGT2B28: 25 of 90 against 51 of 214, P = 0.47; UGT2B17: 25 of 90 against 81 of 214, P = 0.11). In tumour DNA, UGT2B17 loss is less frequent in carriers (GDC: 28 of 113 against 108 of 256, P = 0.0015; Xena: 30 of 112 against 106 of 252, P = 0.0068; uncorrected), whereas loss of UGT2B28 and of the junction interval is not (Supplementary Table S14). At the junction interval, by contrast, the blood-derived normal samples form a single mode around zero (266 of 306 within 0.1), with 2 below -0.3 and none above 0.3. Carriers and non-carriers of the junction did not differ significantly in the copy number of the interval in tumour DNA (median log2 ratio -0.042 against -0.053, P = 0.63), in blood-derived normal DNA (0.007 against 0.005, P = 0.19) or in solid-tissue normal DNA (0.015 against 0.008, P = 0.20), nor in the frequency of segment breakpoints inside the interval (tumour DNA, GDC 9.7% against 10.5%, P = 1.00; Xena 7.1% against 3.2%, P = 0.10) (Supplementary Table S14). In tumour DNA, 90 of 379 (23.7%; GDC) and 93 of 372 (25.0%; Xena) samples show a log2 ratio below -0.3 at the interval, at similar frequencies in carriers and non-carriers (Supplementary Table S14). A germline or somatic deletion joining the two genes is therefore not supported.

Supplementary Table S10b. Segmental duplications (UCSC Genome Browser [48], track genomicSuperDups, GRCh38) containing each splice site of the UGT2B11 to UGT2B27P junction.

Splice site	Duplication containing the site	Partner locus	Identity
acceptor, chr4:69,005,136	chr4:68,968,479–69,020,349	chr4:69,096,536–69,156,138	90.2%
acceptor, chr4:69,005,136	chr4:68,997,728–69,023,443	chr4:69,405,870–69,433,456	92.2%
acceptor, chr4:69,005,136	chr4:68,998,981–69,023,443	chr4:69,277,534–69,301,670	91.9%
acceptor, chr4:69,005,136	chr4:69,003,716–69,023,443	chr4:69,386,521–69,403,218	92.5%
acceptor, chr4:69,005,136	chr4:69,004,421–69,023,443	chr4:69,200,001–69,217,694	92.3%
acceptor, chr4:69,005,136	chr4:69,004,577–69,023,443	chr4:68,812,930–68,831,154	95.9%
donor, chr4:69,204,429	chr4:69,169,963–69,236,140	chr4:69,255,967–69,318,840	95.3%

Splice site	Duplication containing the site	Partner locus	Identity
donor, chr4:69,204,429	chr4:69,187,177–69,220,118	chr4:69,403,437–69,432,223	94.9%
donor, chr4:69,204,429	chr4:69,199,080–69,235,784	chr4:69,364,943–69,403,422	95.1%
donor, chr4:69,204,429	chr4:69,200,001–69,217,694	chr4:69,004,421–69,023,443	92.3%
donor, chr4:69,204,429	chr4:69,200,153–69,218,812	chr4:68,811,816–68,831,151	92.1%

Supplementary Table S14. Copy number of the interval between the splice sites of the UGT2B11 to UGT2B27P junction, by junction-carrier status of the tumour at 3 or more reads (post hoc; exploratory, uncorrected). Log2 ratios are length-weighted means of SNP-array segments; tests are Mann–Whitney U for medians and Fisher exact for proportions. Comparisons use one DNA sample per patient, preferring the sample matching the RNA tumour sample (GDC tumour DNA: 379 samples from 375 patients; Xena tumour DNA: 372 samples from 370 patients; blood-derived and solid-tissue normal DNA: one sample per patient), and are restricted to patients with an RNA tumour sample, from which carrier status is defined; this excludes 6, 6, 2 and 3 patients respectively and gives denominators of 369 (GDC tumour), 364 (Xena tumour), 304 (blood-derived normal) and 80 (solid-tissue normal) patients. The last three rows of each DNA type give the same comparison at UGT2B28 and UGT2B17 (75_ugt2b28_copy_number.py).

DNA	Measure	Carriers	Non-carriers	P
Tumour, GDC (GRCh38)	Interval log2 ratio, median (IQR)	-0.042 (-0.264 to 0.038); n = 113	-0.053 (-0.277 to 0.032); n = 256	0.63
Tumour, GDC (GRCh38)	Interval minus flanks, median (IQR)	0.000 (-0.018 to 0.124); n = 113	0.000 (0.000 to 0.000); n = 256	0.43
Tumour, GDC (GRCh38)	Interval log2 ratio below -0.3	26 of 113 (23.0%)	61 of 256 (23.8%)	0.90
Tumour, GDC (GRCh38)	Interval log2 ratio above +0.3	4 of 113 (3.5%)	7 of 256 (2.7%)	0.74
Tumour, GDC (GRCh38)	Segment breakpoint inside the interval	11 of 113 (9.7%)	27 of 256 (10.5%)	1.00
Tumour, GDC (GRCh38)	Segment breakpoint within 20 kb of a splice site	5 of 113 (4.4%)	13 of 256 (5.1%)	1.00
Tumour, Xena hub (hg19)	Interval log2 ratio, median (IQR)	-0.048 (-0.293 to 0.021); n = 112	-0.059 (-0.298 to 0.020); n = 252	0.80
Tumour, Xena hub (hg19)	Interval minus flanks, median (IQR)	0.000 (0.000 to 0.119); n = 112	0.000 (0.000 to 0.000); n = 252	0.87
Tumour, Xena hub (hg19)	Interval log2 ratio below -0.3	28 of 112 (25.0%)	63 of 252 (25.0%)	1.00
Tumour, Xena hub (hg19)	Interval log2 ratio above +0.3	4 of 112 (3.6%)	7 of 252 (2.8%)	0.74
Tumour, Xena hub (hg19)	Segment breakpoint inside the interval	8 of 112 (7.1%)	8 of 252 (3.2%)	0.10
Tumour, Xena hub (hg19)	Segment breakpoint within 20 kb of a splice site	1 of 112 (0.9%)	5 of 252 (2.0%)	0.67
Blood-derived normal, GDC	Interval log2 ratio, median (IQR)	0.007 (0.001 to 0.057); n = 90	0.005 (-0.002 to 0.037); n = 214	0.19
Blood-derived normal, GDC	Interval minus flanks, median (IQR)	0.000 (0.000 to 0.119); n = 90	0.000 (0.000 to 0.051); n = 214	0.99
Blood-derived normal, GDC	Interval log2 ratio below -0.3	0 of 90 (0.0%)	2 of 214 (0.9%)	1.00

DNA	Measure	Carriers	Non-carriers	P
Blood-derived normal, GDC	Interval log2 ratio above +0.3	0 of 90 (0.0%)	0 of 214 (0.0%)	1.00
Solid-tissue normal, GDC	Interval log2 ratio, median (IQR)	0.015 (0.006 to 0.036); n = 22	0.008 (0.000 to 0.030); n = 58	0.20
Solid-tissue normal, GDC	Interval minus flanks, median (IQR)	0.000 (0.000 to 0.117); n = 22	0.000 (0.000 to 0.000); n = 58	0.12
Solid-tissue normal, GDC	Interval log2 ratio below -0.3	0 of 22 (0.0%)	0 of 58 (0.0%)	1.00
Solid-tissue normal, GDC	Interval log2 ratio above +0.3	0 of 22 (0.0%)	0 of 58 (0.0%)	1.00
Tumour, GDC (GRCh38)	UGT2B28 log2 ratio, median (IQR)	-0.125 (-0.450 to 0.087); n = 113	-0.085 (-0.469 to 0.055); n = 256	0.89
Tumour, GDC (GRCh38)	UGT2B28 log2 ratio below -0.3	43 of 113 (38.1%)	87 of 256 (34.0%)	0.48
Tumour, GDC (GRCh38)	UGT2B17 log2 ratio below -0.3	28 of 113 (24.8%)	108 of 256 (42.2%)	0.0015
Tumour, Xena hub (hg19)	UGT2B28 log2 ratio, median (IQR)	-0.181 (-0.517 to 0.032); n = 112	-0.119 (-0.487 to 0.009); n = 252	0.93
Tumour, Xena hub (hg19)	UGT2B28 log2 ratio below -0.3	48 of 112 (42.9%)	95 of 252 (37.7%)	0.36
Tumour, Xena hub (hg19)	UGT2B17 log2 ratio below -0.3	30 of 112 (26.8%)	106 of 252 (42.1%)	0.0068
Blood-derived normal, GDC	UGT2B28 log2 ratio, median (IQR)	0.003 (-0.454 to 0.106); n = 90	0.003 (-0.220 to 0.022); n = 214	0.87
Blood-derived normal, GDC	UGT2B28 log2 ratio below -0.3	25 of 90 (27.8%)	51 of 214 (23.8%)	0.47
Blood-derived normal, GDC	UGT2B17 log2 ratio below -0.3	25 of 90 (27.8%)	81 of 214 (37.9%)	0.11
Solid-tissue normal, GDC	UGT2B28 log2 ratio, median (IQR)	0.006 (-0.548 to 0.016); n = 22	0.007 (-0.004 to 0.083); n = 58	0.32
Solid-tissue normal, GDC	UGT2B28 log2 ratio below -0.3	8 of 22 (36.4%)	11 of 58 (19.0%)	0.14
Solid-tissue normal, GDC	UGT2B17 log2 ratio below -0.3	2 of 22 (9.1%)	9 of 58 (15.5%)	0.72

S5.4 Concordance of the two normal references

Detection of each junction, defined as at least 2 reads in at least one sample, was compared between the 50 matched normal samples and the 251 GTEx liver samples (Supplementary Table S11, Supplementary Figure S6). Agreement is high for annotated junctions and low for unannotated ones, and discordance at a frequency of 10% or more occurs in both directions. The comparison was repeated genome-wide (Supplementary Table S15, Supplementary Figure S7).

Supplementary Table S11. Junction detection in matched normal tissue (n = 50) and GTEx liver (n = 251) (post hoc). Spearman rho is the correlation of detection fractions over junctions detected in at least one reference. The last four count columns give junctions detected in at least 10% or 50% of one reference with no carrier in the other.

Stratum	Junctions	Matched normal only	GTEx only	Both	Neither	≥ 10% of matched normal, no GTEx carrier	≥ 10% of GTEx, no matched-normal carrier	≥ 50% of matched normal, no GTEx carrier	≥ 50% of GTEx, no matched-normal carrier	Spearman rho
All retrieved junctions, all	1,263,305	3123	25,321	11,666	1,223,195	108	617	10	67	0.500
All retrieved junctions, annotated	7918	58	1172	4796	1892	0	99	0	9	0.935
All retrieved junctions, unannotated	1,255,387	3065	24,149	6870	1,221,303	108	518	10	58	0.238
Canonical motif, all	807,067	2574	16,697	10,130	777,666	95	410	10	51	0.556
Canonical motif, annotated	6577	45	888	4348	1296	0	69	0	7	0.934
Canonical motif, unannotated	800,490	2529	15,809	5782	776,370	95	341	10	44	0.267
Evidence floor, all	12,783	315	2496	9011	961	73	514	10	60	0.839
Evidence floor, annotated	4967	12	362	4536	57	0	94	0	8	0.916
Evidence floor, unannotated	7816	303	2134	4475	904	73	420	10	52	0.585
Evidence floor, upper-case motif rule, all	11,069	277	2086	7980	726	69	392	10	51	0.857
Evidence floor, upper-case motif rule, annotated	4472	9	268	4152	43	0	69	0	7	0.909
Evidence floor, upper-case motif rule, unannotated	6597	268	1818	3828	683	69	323	10	44	0.602

The genome-wide comparison used the recount3 project-level junction matrices of TCGA-LIHC and GTEx liver [28], from which the tcgav2 and gtexv2 compilations are built. Carrier counts recomputed from these matrices were identical to the Snaptron-derived counts for every panel junction (776,855 junctions of the tcgav2 and 770,232 of the gtexv2 tables; 117,448 and 112,161 of them are present in the project matrices, and the remainder have no carrier in either; recount3_validation.tsv). Junctions on chromosomes 1 to 22, X, Y and M were included. The evidence floor takes the motif from the TCGA-LIHC project, which on the panel gives 12,487 rather than 12,783 junctions, because junctions absent from the TCGA-LIHC project matrix have no motif in it; recount3 motifs carry no case, and the upper-case motif rule is therefore not applied genome-wide. Genome-wide, 335,637 junctions pass the evidence floor and detection fractions correlate with Spearman rho = 0.898 (panel, same definition: 0.847). Of the 208,489 junctions detected in at least 10% of GTEx samples, at most 1074.5 would be expected to show no matched-normal carrier by chance if each were carried by at least 10% of matched normal tissue ($0.9^{50} = 0.0052$), against 12,154 observed; for the converse direction the expectation is below 10^{-6}, against 245 observed.

Supplementary Table S15. Junction detection in matched normal tissue (n = 50) and GTEx liver (n = 251), genome-wide (post hoc; recount3 project-level junction matrices). All rows are junctions passing the evidence floor; columns as in Supplementary Table S11. The evidence floor takes the motif from the TCGA-LIHC project; the panel rows apply the same definition to the panel junctions for comparison. The last row also admits junctions present only in GTEx liver, with their GTEx motif.

Scope, annotation	Junctions	Matched normal only	GTEx only	Both	Neither	≥ 10% of matched normal, no GTEx carrier	≥ 10% of GTEx, no matched-normal carrier	≥ 50% of matched normal, no GTEx carrier	≥ 50% of GTEx, no matched-normal carrier	Spearman rho
Panel, all	12,487	315	2200	9011	961	73	440	10	39	0.847
Panel, annotated	4963	12	358	4536	57	0	91	0	8	0.916
Panel, unannotated	7524	303	1842	4475	904	73	349	10	31	0.600
Genome, all	335,637	2590	64,062	238,915	30,070	245	12,154	21	1043	0.898
Genome, annotated	258,128	803	36,148	209,884	11,293	37	7066	6	610	0.919
Genome, unannotated	77,509	1787	27,914	29,031	18,777	208	5088	15	433	0.466
Genome, with GTEx-only, all	342,809	2590	71,234	238,915	30,070	245	13,672	21	1291	0.895

S5.5 Minimum recorded read count by intron length

Per-sample junction counts in both compilations have a minimum that depends on intron length (Supplementary Table S12a). In the recount3 project-level matrices the rule holds for every junction: among the 45,755 TCGA-LIHC and 40,317 GTEx liver junctions with introns longer than 50 kb, no per-sample count below 2 (introns of 50 to 100 kb), 3 (100 to 200 kb) or 4 reads (over 200 kb) is recorded (recount3_extract.txt). The exact-coordinate queries of Supplementary Table S12a show the same minima. The pattern matches the default junction filter of the STAR aligner (parameter outSJfilterIntronMaxVsReadN, STAR manual) [58], which requires at least 2, 3 and 4 supporting reads for introns longer than 50, 100 and 200 kb and applies to junctions not supplied as annotation; the recount3 workflow runs STAR without annotation for junction calling [28]. A normal-side floor of 2 reads is consequently a floor of 3 or 4 reads for such junctions, and the threshold sweep cannot distinguish thresholds below those values. The junctions concerned are counted in Supplementary Table S12b: 320 of the 981 eligible junctions and three of the eight absence-supported junctions have introns longer than 100 kb. The tumour floor of 5 reads is unaffected.

Supplementary Table S12a. Minimum per-sample read count recorded for junctions of increasing intron length (exact-coordinate queries; post hoc).

Junction	Coordinates	Compilation	Intron length (bp)	Carrier samples	Minimum reads	Samples with 1 read	2 reads	3 reads	4 reads
A2M	chr12:9067840–9068182	gtexv2	343	19,001	1	133	158	139	157
A2M	chr12:9067840–9068182	tcgav2	343	11,256	1	22	18	17	7
TERT	chr5:1258660–1260473	gtexv2	1814	3042	1	1286	601	331	231
TERT	chr5:1258660–1260473	tcgav2	1814	4037	1	1581	778	457	254
chr11, 20 kb	chr11:2114971–2135366	gtexv2	20,396	88	1	76	8	2	2
chr11, 20 kb	chr11:2114971–2135366	tcgav2	20,396	473	1	94	47	46	26
chr9, 24 kb	chr9:21971209–21994820	gtexv2	23,612	3221	1	2048	616	251	127

Junction	Coordinates	Compilation	Intron length (bp)	Carrier samples	Minimum reads	Samples with 1 read	2 reads	3 reads	4 reads
chr9, 24 kb	chr9:21971209–21994820	tcgav2	23,612	4690	1	1662	742	458	314
chr6, 64 kb	chr6:29945282–30009534	gtexv2	64,253	18,907	2	0	25	5	15
chr6, 64 kb	chr6:29945282–30009534	tcgav2	64,253	11,297	2	0	25	25	24
chr4, 69 kb	chr4:57040934–57109876	gtexv2	68,943	19,012	2	0	46	37	39
chr4, 69 kb	chr4:57040934–57109876	tcgav2	68,943	11,348	2	0	1	1	0
UGT1A10 to UGT1A1	chr2:233637378–233767033	gtexv2	129,656	1267	3	0	0	91	77
UGT1A10 to UGT1A1	chr2:233637378–233767033	tcgav2	129,656	2075	3	0	0	156	123
UGT1A8 to UGT1A1	chr2:233618563–233767033	gtexv2	148,471	871	3	0	0	137	67
UGT1A8 to UGT1A1	chr2:233618563–233767033	tcgav2	148,471	1687	3	0	0	157	112
UGT2B11 to UGT2B27P	chr4:69005136–69204429	gtexv2	199,294	11	3	0	0	3	1
UGT2B11 to UGT2B27P	chr4:69005136–69204429	tcgav2	199,294	336	3	0	0	50	26
chr6, 207 kb	chr6:29801779–30009144	tcgav2	207,366	479	4	0	0	0	146
CFH to CFHR4	chr1:196690240–196905107	tcgav2	214,868	278	4	0	0	0	11
chr11, 255 kb	chr11:1873879–2129269	gtexv2	255,391	1	4	0	0	0	1
chr11, 255 kb	chr11:1873879–2129269	tcgav2	255,391	122	4	0	0	0	31
chr14, 272 kb	chr14:105469702–105741892	gtexv2	272,191	176	4	0	0	0	44
chr14, 272 kb	chr14:105469702–105741892	tcgav2	272,191	1424	4	0	0	0	263
chr6, 207 kb	chr6:29801779–30009144	gtexv2	not returned						
CFH to CFHR4	chr1:196690240–196905107	gtexv2	not returned						

Supplementary Table S12b. Junctions by intron-length class and the minimum read count recorded in that class.

Set	Up to 50 kb (from 1 read)	50 to 100 kb (from 2 reads)	100 to 200 kb (from 3 reads)	Over 200 kb (from 4 reads)	Total
all retrieved junctions	1,183,223	40,434	24,165	15,483	1,263,305
canonical, recurrent (≥ 112 of 374)	3012	52	68	31	3163
eligible set	569	92	151	169	981
eligible, ≥ 44 of 374	5	0	3	0	8
eligible, ≥ 15 of 374	19	0	3	3	25

S5.6 Normal-side read floor

The normal-side read floor analysis is reported in main text Table 7. With the matched-normal floor raised from 2 to 5 reads and the GTEx floor held at 2 reads, 314 junctions enter the eligible set. One of them reaches 44 of 374 tumours: chr6:29,829,933–29,945,058 (plus strand, unannotated), carried by 44 tumours, by 4 of 50 matched normal samples at 2 or more reads and by none at 5 or more reads, and by no GTEx liver sample.

For the 25 eligible junctions that reach the informative prevalence of the joint reference, per-sample counts were retrieved by exact-coordinate query and carriers counted at floors of 1, 2, 3 and 5 reads (Supplementary Table S13). Both absence criteria are retained by all 25 junctions at floors of 2, 3 and 5 reads and by 15 at a floor of 1 read, including 6 of the 8 supported by the matched-normal criterion; for the six with introns longer than 100 kb, three of them among the eight, floors below the recording minimum cannot be evaluated.

Supplementary Table S13. Normal-side carriers of the 25 eligible junctions reaching 15 of 374 tumours, at read floors of 1, 2, 3 and 5 (exact-coordinate queries; post hoc). For introns longer than 100 kb and 200 kb, samples with fewer than 3 and 4 reads are not recorded.

Junction	Intron length (bp)	Tumours, ≥ 5 reads (of 374)	Matched normal carriers at ≥ 1 / ≥ 2 / ≥ 3 / ≥ 5 reads (of 50)	GTEx liver carriers at ≥ 1 / ≥ 2 / ≥ 3 / ≥ 5 reads (of 251)
chr4:69005136–69204429:−	199,294	88	0 / 0 / 0 / 0	1 / 1 / 1 / 1
chr5:1258660–1260473:−	1814	71	0 / 0 / 0 / 0	2 / 0 / 0 / 0
chr5:1255412–1258597:−	3186	69	0 / 0 / 0 / 0	5 / 0 / 0 / 0
chr5:1266536–1268519:−	1984	69	0 / 0 / 0 / 0	4 / 0 / 0 / 0
chr2:233637378–233767033:+	129,656	61	0 / 0 / 0 / 0	0 / 0 / 0 / 0
chr5:1260601–1264403:−	3803	57	0 / 0 / 0 / 0	0 / 0 / 0 / 0
chr5:1254506–1255286:−	781	52	0 / 0 / 0 / 0	1 / 0 / 0 / 0
chr2:233618563–233767033:+	148,471	44	0 / 0 / 0 / 0	0 / 0 / 0 / 0
chr5:1264593–1266463:−	1871	41	0 / 0 / 0 / 0	3 / 1 / 0 / 0
chr5:1268634–1271118:−	2485	40	0 / 0 / 0 / 0	0 / 0 / 0 / 0
chr5:1280339–1282428:−	2090	38	0 / 0 / 0 / 0	0 / 0 / 0 / 0
chr5:1278797–1279290:−	494	37	1 / 0 / 0 / 0	5 / 0 / 0 / 0
chr5:1272281–1278640:−	6360	35	1 / 0 / 0 / 0	1 / 0 / 0 / 0
chr5:1271205–1272184:−	980	32	0 / 0 / 0 / 0	1 / 0 / 0 / 0
chr5:1279471–1280157:−	687	31	0 / 0 / 0 / 0	0 / 0 / 0 / 0

Junction	Intron length (bp)	Tumours, ≥ 5 reads (of 374)	Matched normal carriers at ≥ 1 / ≥ 2 / ≥ 3 / ≥ 5 reads (of 50)	GTEx liver carriers at ≥ 1 / ≥ 2 / ≥ 3 / ≥ 5 reads (of 251)
chr11:2140300–2140856:−	557	30	3 / 0 / 0 / 0	11 / 1 / 0 / 0
chr5:1268634–1278640:−	10,007	30	1 / 0 / 0 / 0	2 / 0 / 0 / 0
chr11:2114971–2135366:−	20,396	29	0 / 0 / 0 / 0	8 / 1 / 1 / 0
chr11:1873879–2129269:−	255,391	24	0 / 0 / 0 / 0	0 / 0 / 0 / 0
chr5:1254665–1255286:−	622	21	0 / 0 / 0 / 0	0 / 0 / 0 / 0
chr14:105469702–105741892:−	272,191	20	0 / 0 / 0 / 0	0 / 0 / 0 / 0
chr3:133754672–133756830:+	2159	20	4 / 0 / 0 / 0	16 / 2 / 0 / 0
chr6:29801779–30009144:+	207,366	18	0 / 0 / 0 / 0	0 / 0 / 0 / 0
chr9:21971209–21994820:−	23,612	17	2 / 0 / 0 / 0	6 / 2 / 1 / 0
chr5:1282625–1293312:−	10,688	15	0 / 0 / 0 / 0	2 / 0 / 0 / 0

S5.7 Nested and patient-grouped split-half evaluation

The split-half evaluation of main text Section 3.5 was repeated from per-sample counts of the recount3 matrices, which reproduce the panels and held-out coverage of main text Table 4 exactly (variant a). The 374 tumour samples come from 371 patients; in variants b and d, all samples of a patient fall into the same half, and both halves keep 187 samples. In variants c and d, the candidate pool is restricted to eligible junctions detected in at least one training tumour, and the eight-junction subset is re-derived from training-half prevalence with the rule of main text Section 3.4, which requires at least 22 of 187 tumours. The re-derived subset differs from the full-cohort eight in 39 of 50 splits (variant c), with a median of 8 members (range 6 to 15). Across variants and panel sizes, held-out coverage differs from variant a by less than 1 percentage point (Supplementary Table S16); the optimism attributable to full-cohort selection of the subset is 0.03 percentage points at a budget of 20 targets.

Supplementary Table S16. Held-out tumour coverage, mean (SD) in %, of greedy panels by panel size, over 50 split-half partitions with the seed of main text Section 3.5 (post hoc). a, splits by sample, pool and subset defined on all 374 tumours (the evaluation of main text Table 4); b, splits by patient; c, nested: pool and eight-junction subset re-derived within each training half; d, nested and split by patient. Subset (8): the eight-junction subset of main text Section 3.4.

Pool	Variant	1	2	3	5	10	20	50
Eligible set (981)	a	23.2 (2.8)	35.7 (3.1)	44.3 (3.5)	53.5 (3.4)	59.9 (3.3)	63.9 (4.0)	67.5 (3.4)
Eligible set (981)	b	22.7 (3.0)	36.6 (2.4)	43.8 (3.2)	53.6 (2.6)	59.3 (2.9)	63.4 (3.3)	67.1 (2.9)
Eligible set (981)	c	23.2 (2.8)	35.7 (3.1)	44.3 (3.5)	53.5 (3.4)	59.9 (3.3)	63.9 (4.0)	67.5 (3.4)
Eligible set (981)	d	22.7 (3.0)	36.6 (2.4)	43.8 (3.2)	53.6 (2.6)	59.3 (2.9)	63.4 (3.3)	67.1 (2.9)
Subset (8)	a	23.2 (2.8)	35.7 (3.1)	45.7 (3.0)	48.2 (3.1)	48.5 (3.2)	48.5 (3.2)	48.5 (3.2)
Subset (8)	b	22.7 (3.0)	36.6 (2.4)	45.4 (2.5)	47.9 (2.4)	48.4 (2.4)	48.4 (2.4)	48.4 (2.4)
Subset (8)	c	23.2 (2.8)	35.7 (3.1)	45.6 (3.2)	48.2 (3.1)	48.5 (3.1)	48.5 (3.1)	48.5 (3.1)

Pool	Variant	1	2	3	5	10	20	50
Subset (8)	d	22.7 (3.0)	36.6 (2.4)	45.4 (2.5)	47.9 (2.4)	48.4 (2.4)	48.4 (2.4)	48.4 (2.4)

S5.8 The pre-specified cascade applied genome-wide

The pre-specified cascade of main text Section 2.4 was applied to every junction on chromosomes 1 to 22, X, Y and M of the recount3 project-level matrices of TCGA-LIHC and GTEx liver, with unchanged thresholds and without a motif filter, as for the panel (main text Section 3.1). Applied to the 1,263,305 panel junctions, the same computation gives 3,314, 1 and 0 junctions at the three steps, and the 3314 junctions meeting the recurrence criterion are identical to those of the Snaptron-derived tables. Genome-wide, 108,205 junctions meet the recurrence criterion (107,618 with a GT..AG or CT..AC dinucleotide pair irrespective of letter case, 106,750 annotated), 55 also meet the GTEx criterion and 18 also meet the matched-normal criterion (Supplementary Table S17). Tumour carriers recounted from the per-sample matrix agree with the group counts for all 18. 7 of the 18 carry a lower-case dinucleotide at one or both splice sites in Snaptron, that is, a splice site within soft-masked repeat sequence (main text Section 2.4). Four have introns of 106 to 128 kb, for which counts below 3 reads are not recorded (Supplementary Section S5.5); none of the four has a recorded count in any matched normal sample. In 5 of the 18, one or two matched normal samples carry a single read, below the 2-read signal floor. Locus expression was computed over all other junctions on the same strand within the span of the overlapping GENCODE v26 genes; for the chromosome 2 junction, which overlaps no gene, it was computed within 10 kb of the junction, and at the SSX locus the span of the two longest junctions includes SSX4 and the intervening pseudogenes. The GTEx samples labelled bone marrow are the K-562 chronic myeloid leukaemia cell line (204 samples) and are reported separately from whole blood (852 samples).

Supplementary Table S17. Junctions meeting the three pre-specified criteria genome-wide (post hoc; recount3 project-level junction matrices and exact-coordinate queries of tcgav2 and gtexv2). Gene: GENCODE v26 genes on the junction strand, pseudogenes listed only where no other gene overlaps. Motif as returned by Snaptron, lower case marking soft-masked sequence. Matched normal carriers at 1 or more reads and GTEx liver carriers at 2 or more reads. Other TCGA tumours: tumour samples of TCGA projects other than TCGA-LIHC carrying 5 or more reads. Locus reads: mean reads per sample over the other same-strand junctions of the locus, in matched normal tissue and GTEx liver, as a percentage of the tumour value.

Junction	Intron length (bp)	Gene	Annotated; motif	Tumours, ≥ 5 reads (of 374)	Matched normal ≥ 1 read (of 50) / GTEx liver ≥ 2 reads (of 251)	Other TCGA tumours, ≥ 5 reads	GTEx testis / whole blood / K-562, ≥ 2 reads (of 410 / 852 / 204)	Locus reads, matched normal / GTEx liver (% of tumour)
chr10:17,399,203–17,408,062 (+)	8860	ST8SIA6-AS1	yes; gt..AG	171	2 / 2	1019	390 / 7 / 196	0.9 / 0.7
chr2:81,548,748–81,570,321 (+)	21,574	none	yes; GT..ag	154	0 / 1	1641	288 / 0 / 197	2.6 / 1.0
chr22:12,603,534–12,604,669 (+)	1136	FRG1GP	yes; GT..AG	116	1 / 2	886	106 / 2 / 202	36.3 / 5.5
chr3:27,821,725–27,824,649 (+)	2925	LINC01980	yes; gt..AG	115	2 / 0	1828	68 / 1 / 188	0.0 / 1.3
chr3:27,824,749–27,860,086 (+)	35,338	LINC01980	yes; GT..AG	133	1 / 1	1953	193 / 0 / 194	0.0 / 1.3
chr3:178,859,624–178,860,293 (-)	670	KCNMB2-AS1	yes; ct..ac	124	1 / 0	3057	7 / 0 / 198	2.0 / 4.5
chr4:46,735,242–46,844,959 (-)	109,718	COX7B2	yes; CT..AC	138	0 / 1	562	396 / 1 / 109	0.0 / 0.8
chr4:46,845,015–46,909,159 (-)	64,145	COX7B2	yes; CT..ac	133	0 / 1	507	398 / 1 / 161	0.0 / 0.8
chr7:119,800,875–119,907,292 (-)	106,418	LVCAT5	yes; CT..ac	116	0 / 0	299	142 / 1 / 202	0.0 / 0.2
chr8:83,404,070–83,407,691 (+)	3622	LINC01419	yes; gt..ag	131	0 / 0	234	120 / 0 / 151	0.0 / 0.0
chrX:48,257,311–48,257,745 (+)	435	SSX1	yes; GT..AG	113	0 / 0	226	396 / 4 / 202	0.2 / 0.1
chrX:48,257,861–48,258,535 (+)	675	SSX1	yes; GT..AG	115	0 / 1	245	396 / 4 / 202	0.2 / 0.1
chrX:48,258,632–48,261,765 (+)	3134	SSX1	yes; GT..AG	121	0 / 0	251	398 / 3 / 203	0.2 / 0.1

Junction	Intron length (bp)	Gene	Annotated; motif	Tumours, ≥ 5 reads (of 374)	Matched normal ≥ 1 read (of 50) / GTEx liver ≥ 2 reads (of 251)	Other TCGA tumours, ≥ 5 reads	GTEx testis / whole blood / K-562, ≥ 2 reads (of 410 / 852 / 204)	Locus reads, matched normal / GTEx liver (% of tumour)
chrX:48,261,816–48,263,781 (+)	1966	SSX1	yes; GT..AG	125	0 / 0	830	397 / 4 / 202	0.2 / 0.1
chrX:48,261,816–48,324,064 (+)	62,249	SSX1	no; GT..AG	113	0 / 0	527	383 / 2 / 202	0.2 / 0.1
chrX:48,261,816–48,389,345 (+)	127,530	SSX1, SSX4	no; GT..AG	113	0 / 0	527	363 / 0 / 202	0.1 / 0.0
chrX:48,263,918–48,266,286 (+)	2369	SSX1	yes; GT..AG	123	0 / 0	845	396 / 3 / 202	0.2 / 0.1
chrX:48,263,918–48,391,868 (+)	127,951	SSX1, SSX4	no; GT..AG	119	0 / 0	738	386 / 2 / 202	0.1 / 0.0

Supplementary Tables

Supplementary Table S1. Threshold sweep of junctions detected in at least 1 tumour (post hoc). Counts are computed on the case-insensitive canonical set (Supplementary Section S2).

Read threshold	Annotation	Junctions (at least 1 tumour)	Median recurrence	Maximum recurrence	Reaching 112
3	unannotated	3929	1	114	1
3	annotated	273	1	97	0
5 (pre-specified)	unannotated	1178	1	88	0
5 (pre-specified)	annotated	114	2	71	0
10	unannotated	380	1	53	0
10	annotated	56	2	46	0
20	unannotated	162	1	26	0
20	annotated	32	7.5	34	0

Supplementary Table S2. Library QC, four cancer libraries.

Metric	SRR10822542 (cancer_3)	SRR10822548 (cancer_9)	SRR10822570 (cancer_31)	SRR10822572 (cancer_33)
Read pairs after trimming	14,520,533	24,783,270	31,417,387	29,818,129
Read length R1/R2 (bp)	132/129	142/135	132/126	133/129
Duplication rate	0.423	0.419	0.536	0.544
Alignment rate, 1M-read QC subsample	69.39%	91.41%	85.53%	91.05%
rRNA fraction	0.0221	0.0192	0.1534	0.1278
Globin fraction	0.0077	0.0032	0.0005	0.0006
Exonic base fraction	0.4995	0.8076	0.8604	0.7560
Strandedness (inferred)	RF	RF	RF	RF

Supplementary Table S3. Per-gene Method 1 maxima, four cancer libraries.

Gene	SRR10822542	SRR10822548	SRR10822570	SRR10822572	Detected in
ALB	124	655	49	235	4 of 4
SERPINA1	208	220	590	400	4 of 4
FGA	155	53	14	125	4 of 4
APOA1	0	5	8	36	3 of 4
HP	0	12	4	50	3 of 4
FGB	0	46	0	82	2 of 4
FGG	0	12	0	32	2 of 4
TF	0	65	0	27	2 of 4

Supplementary Table S4. Negative and specificity controls, per library.

Library	Negative controls detected	Median reads among detected	Specificity controls detected
SRR10822542 (cancer_3)	35 of 45	628.0	0 of 3
SRR10822548 (cancer_9)	44 of 45	1932.5	0 of 3
SRR10822570 (cancer_31)	42 of 45	975.5	0 of 3
SRR10822572 (cancer_33)	44 of 45	1532.5	0 of 3
SRR10822600 (healthy_26)	30 of 45	44.0	0 of 3

Supplementary Figures

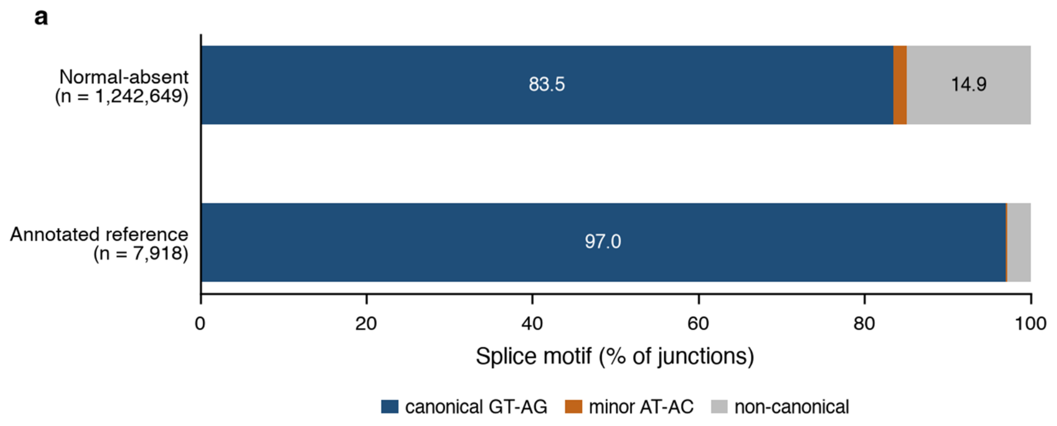

Supplementary Figure S1. Of the normal-absent junctions, 14.9% carry non-canonical splice motifs, against 2.8% of annotated junctions. Splice-motif composition of the normal-absent set against the annotated GENCODE v26 junctions of the same genes, from motif_composition.tsv. Motifs are the donor and acceptor dinucleotides reported by Snaptron read on the plus strand; canonical means GT-AG or its minus-strand representation CT-AC. Percentages are of junctions within each set. The annotated set serves as an internal reference for genuine splicing in these data. Post-hoc analysis. The normal-absent set is 83.5% canonical and 1.6% minor-spliceosome, against 97.0% canonical for the annotated reference (n = 7918). Motifs (matched without regard to letter case) and annotation are taken from either compilation; the excess of non-canonical motifs is consistent with a non-splicing component.

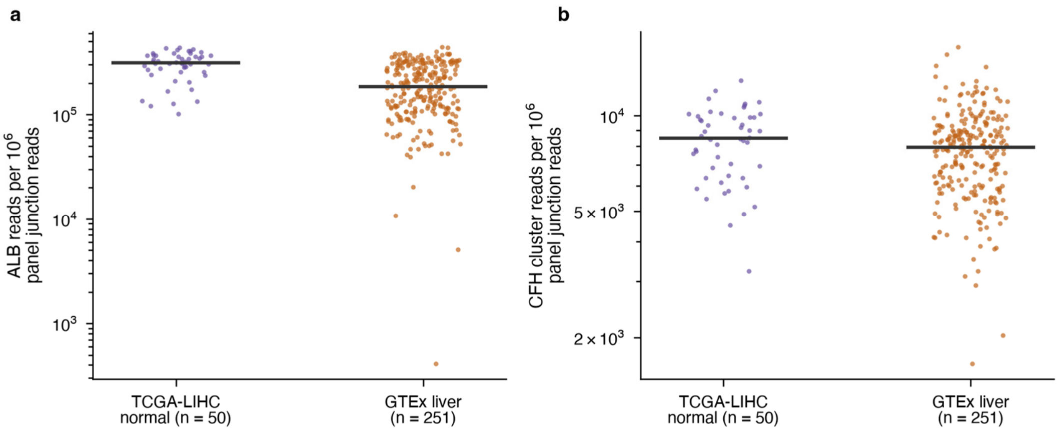

Supplementary Figure S2. TCGA adjacent normal and GTEx liver are not interchangeable as a normal reference. **a**, Depth-normalised ALB locus junction coverage in TCGA-LIHC matched normal against GTEx liver, from cfh_tissue_state.tsv, one point per sample with group medians as horizontal bars. **b**, The same comparison for the CFH gene cluster (CFH, CFHR1, CFHR2, CFHR3), which did not differ significantly between the cohorts. Post-hoc analysis. ALB is 1.70-fold higher in TCGA-LIHC normal than in GTEx liver (Mann–Whitney $p = 1.57 \times 10^{-9}$) whereas the CFH cluster differs by 1.07-fold ($p = 0.107$). A junction at the CFH locus that passed the GTEx criterion was carried by none of 251 GTEx liver samples and by 47 of 50 matched normals; the mechanism is undetermined (Supplementary Section S1).

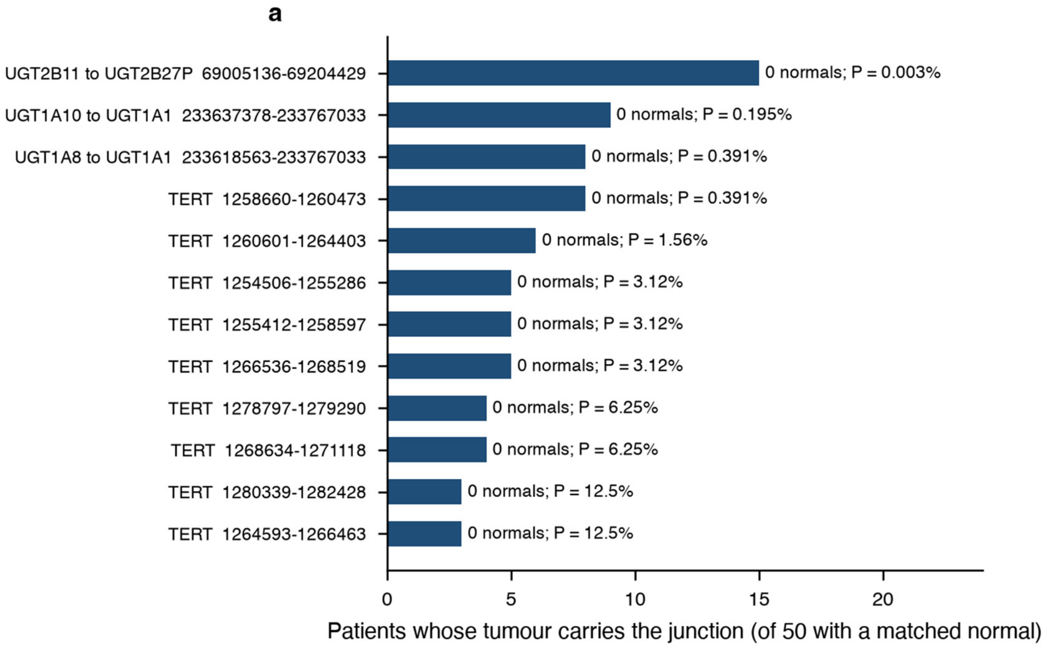

Supplementary Figure S3. Paired tumour carriers of the 12 most recurrent canonical, normal-absent junctions among the 50 patients with a matched normal. Paired matched-normal comparison from paired_normal_test.tsv. For each of the 12 canonical, normal-absent junctions with the highest recurrence, bars give the number of patients whose own tumour carries the junction among the 50 with a matched normal; the annotation gives the number of those same patients carrying it in their own normal and the value 0.5^m, where m is the number of paired tumour carriers, which is the probability of zero carriers among m normals under an illustrative null in which the junction is present in half of normals. Samples are paired on the TCGA patient barcode. Gene pairs are named in transcriptional order. Post-hoc analysis. The 12 junctions were selected for having no carrier at 2 or more reads among these same 50 matched normals; the count of 0 own-normal carriers is therefore fixed by the selection and is not an independent observation, and the 0.5^m values are illustrative arithmetic, not tests. The junctions have 3 to 15 paired tumour carriers; four have at least 8 (0.5^m at most 0.0039) and four have at most 4 (0.5^m at least 0.0625). Carrier counts are per patient; the junction carried by 88 tumour samples is carried by 87 patients.

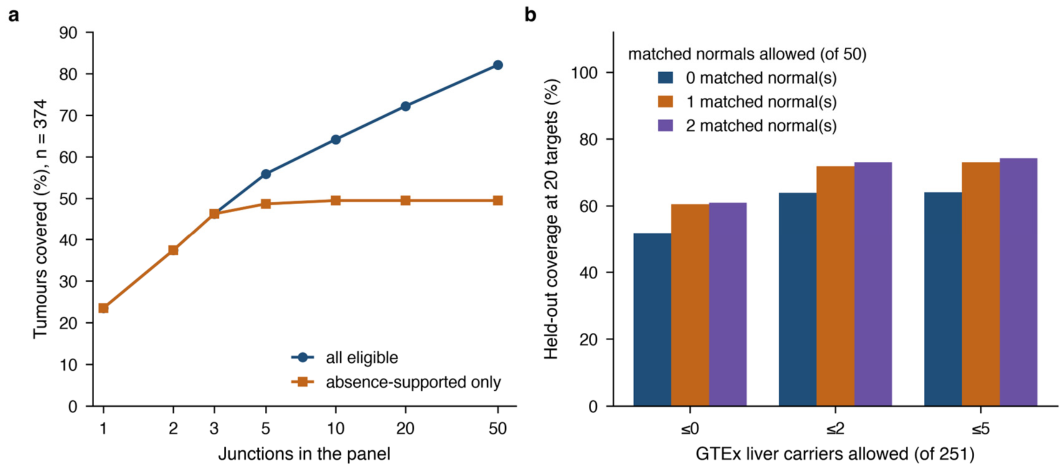

Supplementary Figure S4. The coverage a multi-target panel reaches depends on which junctions are allowed into it, and on the amount of normal-tissue signal the eligibility criterion tolerates. a, Greedy coverage on the full cohort before and after removing junctions whose absence from normal tissue is not statistically supported, from false_absence_supported_coverage.tsv, under the rule of main text Section 3.4. The two curves are identical for the first three targets and diverge thereafter; the supported subset saturates while the full eligible set continues to rise. **b**, Held-out coverage of a 20-target panel across the normal-side criterion grid, from normal_side_sweep.tsv, using the split-half procedure of main text Figure 6b. Coverage rises monotonically as the criterion is relaxed. Post-hoc analysis; thresholds.json is unchanged.

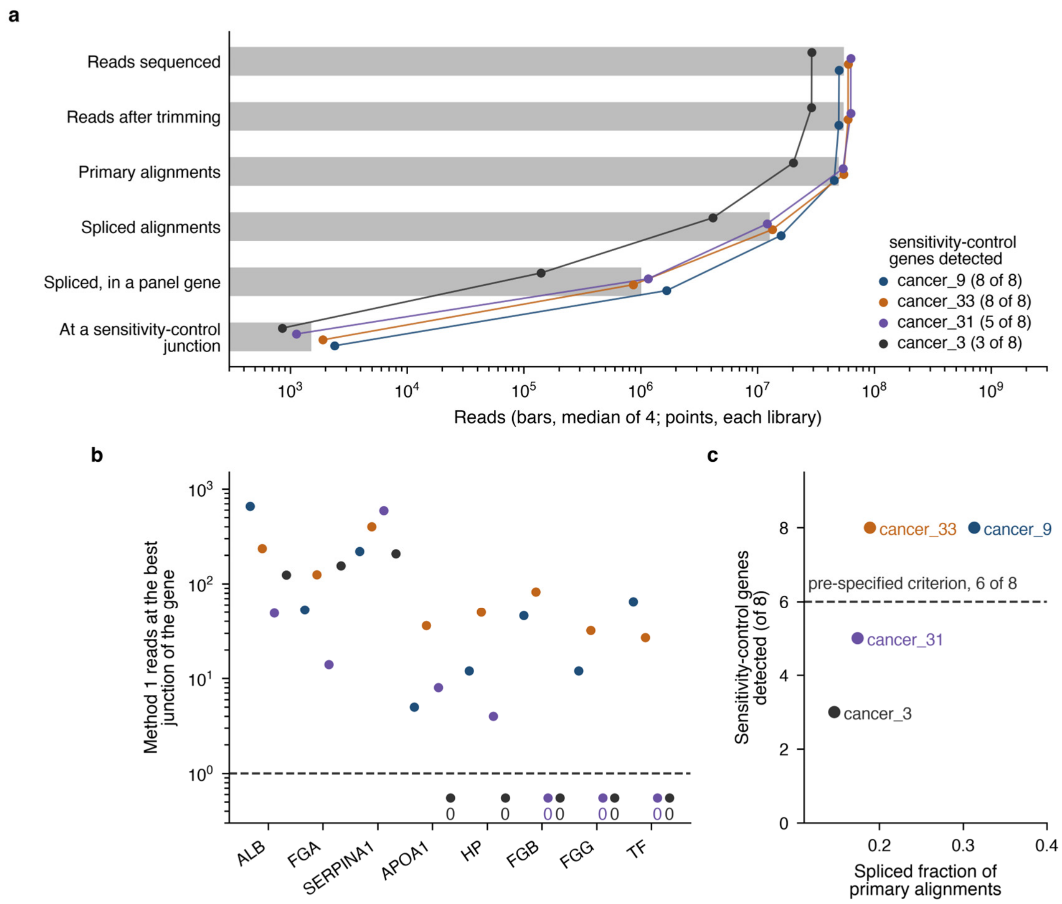

Supplementary Figure S5. Control junctions are recovered from all four cancer libraries, two of which detect all 8 control genes while the designated library does not, and detection tracks the spliced fraction of the library more closely than its read count. a, Read attrition from sequenced reads to sensitivity-control junctions across the four plasma cell-free RNA libraries, from detection_funnel.tsv. Bars are four-library medians and points are individual libraries. Every stage is expressed in reads rather than read pairs; the funnel is therefore monotonic, and stages from primary alignments onward count primary alignments only. **b**, Method 1 reads at the best junction of each of the eight pre-specified sensitivity-control genes, from per_library_sensitivity.tsv. The axis is logarithmic; genes with no reads are therefore drawn below the axis floor and labelled 0; the dashed line is one junction-spanning read. Colours are the same libraries as in **a**. **c**, Sensitivity-control genes detected against the spliced fraction of primary alignments, one point per library, with the pre-specified criterion of 6 of 8 drawn. With n = 4, the rank correlations are descriptive; no trend was fitted and no p-value was computed. Post-hoc analysis, except for the primary library, on which the pre-specified criterion is defined.

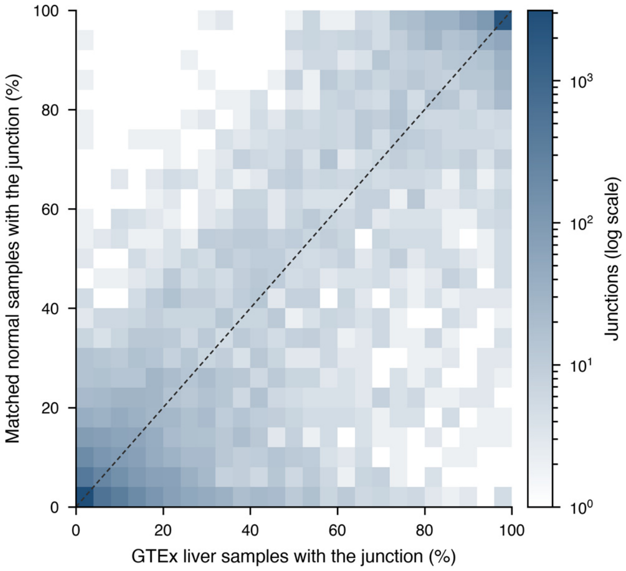

Supplementary Figure S6. The two normal references agree on common junctions and disagree on a minority. Detection fraction of each junction passing the evidence floor (n = 12,783) in the 50 matched normal samples against the 251 GTEx liver samples, at 2 or more reads, binned in two dimensions with a logarithmic colour scale; the dashed line is equality. Spearman rho = 0.839 over the 11,822 junctions detected in at least one reference. Junctions along the axes are detected in one reference only: 73 are carried by at least 10% of matched normal samples and by no GTEx sample, and 514 show the converse. From reference_concordance_bins.tsv. Post-hoc analysis.

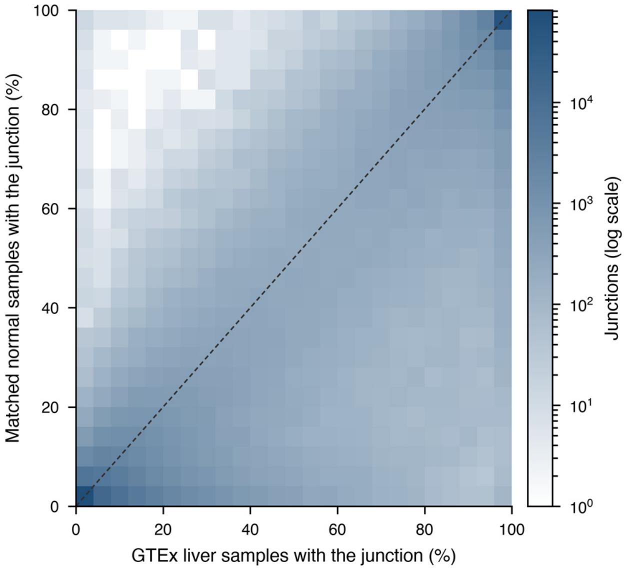

Supplementary Figure S7. The pattern of agreement between the two normal references holds genome-wide. Detection fraction of each junction passing the evidence floor genome-wide (n = 335,637; recount3 project-level junction matrices) in the 50 matched normal samples against the 251 GTEx liver samples, at 2 or more reads, binned in two dimensions with a logarithmic colour scale; the dashed line is equality. Spearman rho = 0.898 over the 305,567 junctions detected in at least one reference. Post-hoc analysis; data from reference_concordance_genome_bins.tsv.
